# Supplementary material for: Efficiency stagnation in global steel production urges joint supply- and demand-side mitigation efforts
Source: Nat Commun. 2021 Apr 6;12:2066. doi: 10.1038/s41467-021-22245-6 (PMC8024266; doi:10.1038/s41467-021-22245-6)
Supplement: Supplementary file 1 — Supplementary Information [file 41467_2021_22245_MOESM1_ESM.pdf]

SUPPLEMENTARY INFORMATION:

Efficiency Stagnation in Global Steel Production Urges Joint Supply- and Demand-side Mitigation Efforts

Peng Wang [1,2], Morten Ryberg [3*], Yi Yang [1,4,5], Kuishuang Feng [6,7], Sami Kara [2*], Michael Hauschild [3], and Wei-Qiang Chen [1,8*]

[1] Key Lab of Urban Environment and Health, Institute of Urban Environment, Chinese Academy of Sciences, Xiamen, China

[2] Sustainability in Manufacturing and Life Cycle Engineering Research Group, School of Mechanical and Manufacturing Engineering, The University of New South Wales, Sydney, Australia

[3] Quantitative Sustainability Assessment Group, Sustainability Division, Department of Technology, Management and Economics, Technical University of Denmark, Kgs. Lyngby, Denmark

[4] Key Laboratory of the Three Gorges Reservoir Region's Eco-Environment, Ministry of Education, Chongqing University, Chongqing 400045, China

[5] Environmental Studies Program, Dartmouth College, Hanover, New Hampshire, USA

[6] Institute of Blue and Green Development, Shandong University, Weihai, China

[7] Department of Geographical Sciences, University of Maryland, College Park, MD, USA

[8] University of Chinese Academy of Sciences, Beijing, China

*Corresponding authors: M. R. (moryb@dtu.dk), S. K. (s.kara@unsw.edu.au), and W-Q C. (wqchen@iue.ac.cn).

TABLE OF CONTENTS:

S1. Overview 3

S1.1 System description 4

S1.2 Process technologies and their historical development 5

S2. Quantitative Method 9

S2.1 Material Flow Quantification 9

S2.2 GHG Emission Calculation 18

S2.3 Uncertainty analysis 30

S3. Results and their uncertainties 42

S3.1 Material Flow Results 42

S3.2 GHG Emission Results 45

S3.3 Results evaluation 48

S3.4 GHG intensity results 50

S4. Additional results and figures 51

S4.1 The share of GHG emission from steel production from 1990 to 2015 51

S4.2 Primary and secondary production and emission data 52

S4.3 Regional material and environmental performance analysis 56

S4.4 Settings of future scenarios 58

S4.5 Historical measures to improve steel production efficiency 60

S4.6 Breakthrough low-carbon steel production technologies 62

S4.7 Summary of material efficiency strategies related to steel use 72

Supplementary References 75

S1. Overview

Our analysis is performed based on the procedures given in Fig.S1.1, which include three major steps. In Section S1, we first clarify our system boundary, and explore the historical development of the studied processes (and technologies) through the compressive investigation of various technical reports and publications. Secondly, in the entire Section S2, we present detailed procedures for our material flow analysis and environmental impact analysis. This allows for generating results related to material stocks and flows throughout each studied process along the global steel cycle from 1900 to 2015, as well as the trend in greenhouse gas (GHG) emission of each studied process during 1900-2015. By combining these two sets of results, we obtain the historical trend of total GHG emissions from global steel production in the past 115 years, and perform uncertainty analysis of the results (the method is described in Section S2.3 and the results are presented in Section S3). Furthermore, in Step 3 (to enrich the implications of our results), we further deepen our analysis of the global level by looking at regional aspects. This is done by separating the World into 8 regions (i.e. Europe, North America, Developed Asia and Oceania, China, India, Developing Asia and Middle East, Latin America and Caribbean, Africa). Here, we further quantify the regional material stocks and flows since 1995, and examine their GHG emission intensity performance. Meanwhile, we also investigate different types of low-carbon technologies related to steel production and perform a scenario analysis until 2050 to provide recommendations on the required mitigation strategies for achieving climate change targets.

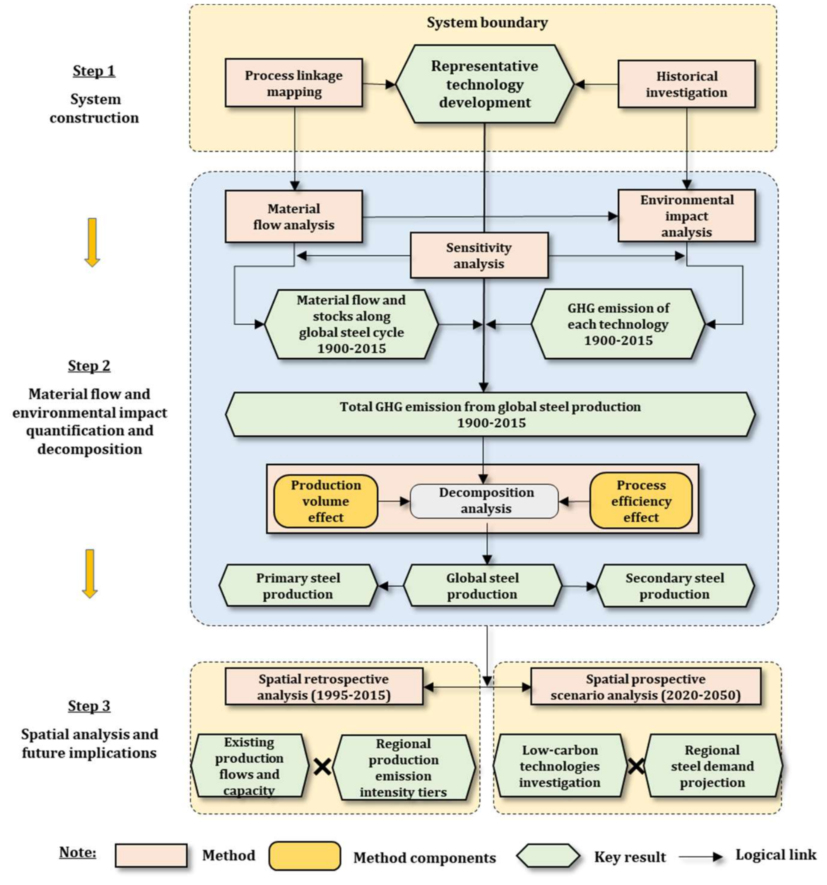

Fig.S1.1 Schematic diagram of our analytical methods

S1.1 System description

The global steel life cycle system is constructed in Fig. S1.2, which consists of 5 main stages (i.e. Mining, Material Preparation, Ironmaking, Steelmaking, and Steel finishing) in the material production system (as the main scope for further emission calculation), and the remaining three life cycle stages (i.e. Fabrication, In-use and End-of-life). The main features of this structure are summarized as follows: Firstly, the material production stages from mining to steelmaking are constructed based on the guidance from World steel association [1]. Furthermore, The linkage from steelmaking, finishing, to fabrication is constructed based on [2]. Thirdly, this study follows the treatment in [3] for the following stages like Fabrication, In-use and End-of-life, which are split and calculated in four end-use sectors (i.e. Construction, Vehicles, Machinery, and Daily goods). Finally, for the first time, several older technologies (i.e. Pudding, Bessemer, and Crucible) are included in our structure for the material flow and environmental impact analysis.

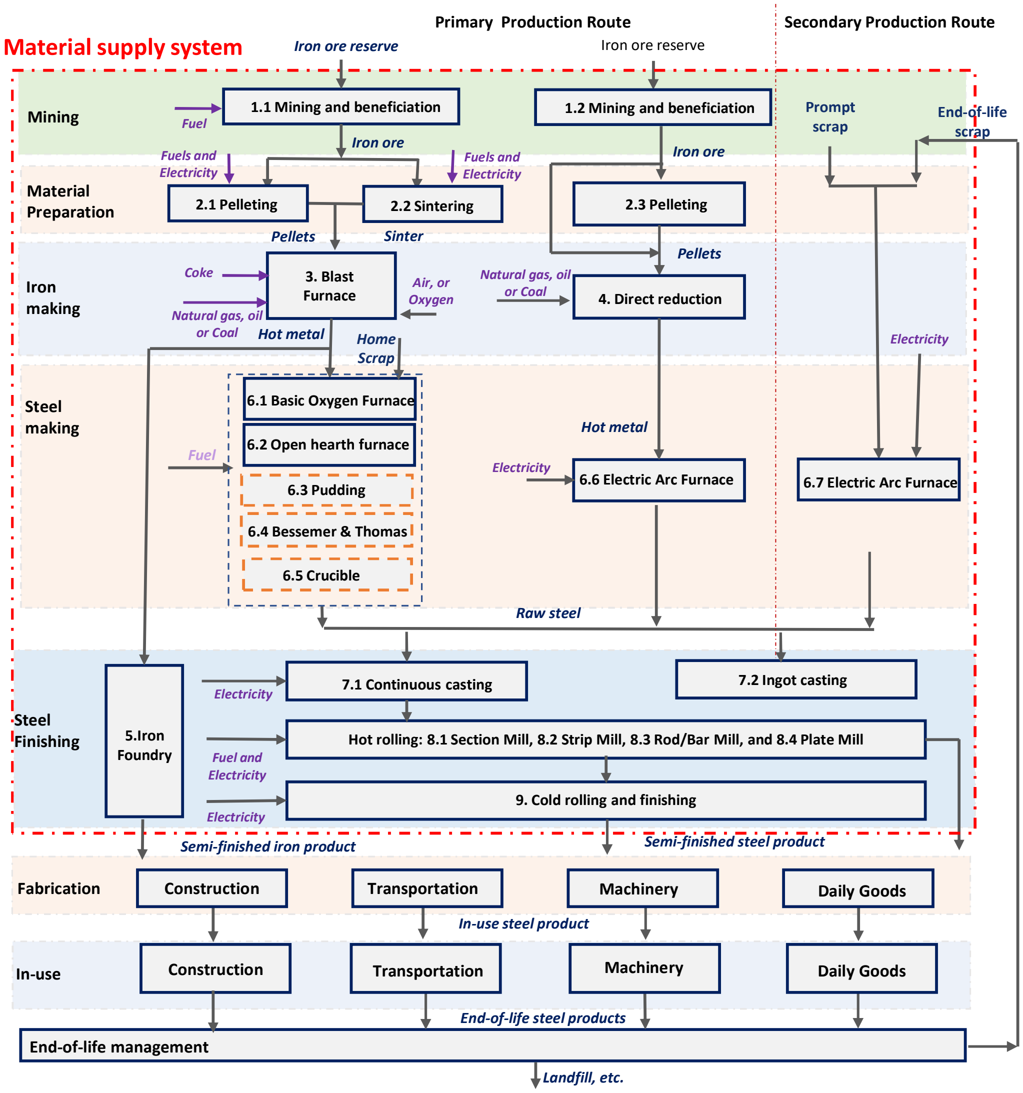

Fig. S1.2 Schematic description of anthropogenic steel cycle

S1.2 Process technologies and their historical development

The total environmental impact of steel production is a sum of the impact from each production process as shown in Fig. S1.2, which consists of around 19 types of processes (i.e. Mining, Sintering and Pelletizing in ore preparation, Blast furnace and Direct reduction in ironmaking, Pudding, Bessemer and Thomas, Open hearth Furnace, Basic oxygen furnaces, Crucible, and Electric arc furnace for steelmaking, Iron foundry, Continuous casting and Ingot casting in steel casting stage, Section mill, Rod and Bar mill, Plate mill, and Hot strip mill in the hot rolling process, and Cold rolling and finishing process). The brief description and the historical application of each process and their related technology are given as follows:

(1) Mining and beneficiation (period: 1900-2015)

The mining stage includes two processes: firstly, it is to extract iron ore from lithosphere through open-pit or underground mining technology. If the ore grade is high enough, then it would be sold directly. Otherwise, in the second place, it would be treated in the beneficiation process to be purified as marketable iron ore. Tailings would be generated as resource losses in this stage. The quantitative model of its yield rate can be found in the Page 4 of reference [4]. In this study, this stage is treated as a whole process, and the studied period of this process is from 1900 to 2015.

(2) Ore preparation (period: 1900-2015 for sintering; 1950-2015 for pelletizing)

Ore preparation is conducted for iron ore agglomeration to produce iron ore for ironmaking. There are four main techniques in history: briquetting, nodulizing, sintering, and pelletizing [5]. Herein, the sintering and pelletizing are considered in this study as there are processes of major importance for modern iron production after 1900 [6]. The original of sintering and pelletizing can be traced back to the 1880s and the 1940s, respectively [6]. Hence, the application period of sintering and pelleting in this study is assumed to be 1900-2015 and 1950-2015, respectively. Notably, according to [1], the ore products from sintering and pelletizing can be fed into the blast furnace process, as shown in Fig. S1.2. By contrast, the direct reduction requires the product from pelletizing or the ore from mining.

(3) Blast Furnace (period: 1900-2015)

Blast Furnace (BF) is the main enabled_technology for ironmaking to produce pig iron in a huge furnace. The modern blast furnace was applied since the 18th century [7]. Hence, the application period of the blast furnace in this study is assumed to be 1900-2015. During this period, blast furnace has experienced significant changes by impressive innovations in its capacity, auxiliary systems, and productivities [8]. Meanwhile, as shown in Fig. S1.3 [9], blast furnace dominates (i.e. around 40%) the total energy use and emission of the entire steel production. Due to its importance, the historical trend of key features (i.e. pig iron production, yield rate, energy efficiency, etc.) of BF has been widely investigated in various publications like [10–14]. Consequently, given the large portion of the blast furnace in total emission and activities results, high accuracy can be guaranteed the total impact and material flow analysis in the steel industry from 1900 to 2015.

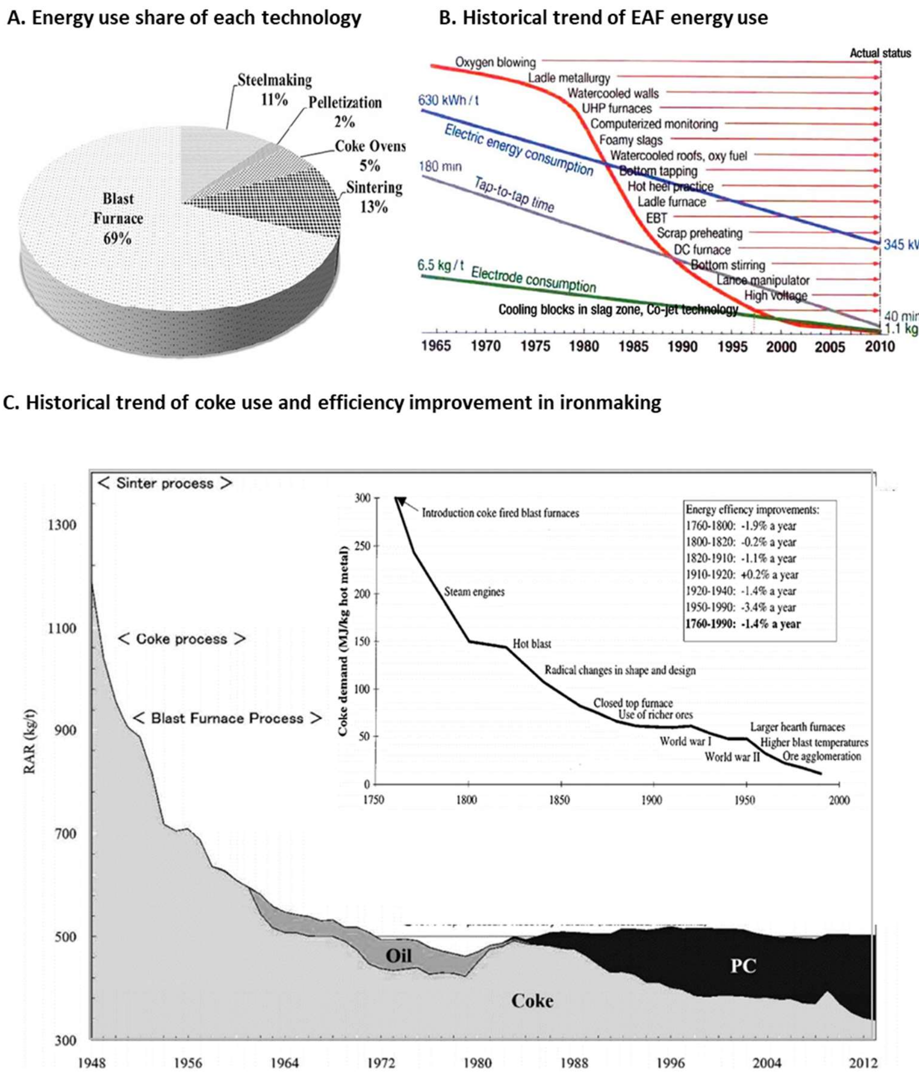

Fig. S1.3 Key indicators of energy use in steel production (A. The share of carbon emission from different technologies [15]; B. The improvement of energy use and other key inputs in EAF [16]; C. The improvement of coke use [10] and energy use [17] in ironmaking process)

(4) Direct reduction process (period: 1970-2015)

Apart from blast furnace technology, the direct reduction is an alternative route to steelmaking, in which the ore is maintained entirely in the solid-state. Commercially, there are three main enabled techniques to produce direct reduction iron (DRI), namely MIDREX[1], HYL/Energiron[2], and Coal-based Rotary Kiln. Meanwhile, the detailed descriptions for those techniques can be found in [18]. Due to its limited share compared to BF, those DR techniques are treated as one process in this study. The production data of DR can be found in World Direct Reduction Statistics [19], which traced the production data since 1970. Accordingly, the application period of direct reduction is assumed to be 1970-2015.

---

[1] Midrex is a gas-based shaft furnace process that converts iron oxides – in the form of pellets or lump ore – into direct reduction iron (DRI).

[2] HYL process is designed for the conversion of iron ore (pellet/lump ore) into metallic iron, by the use of reducing gases in a solid-gas moving bed reactor.

(5) Ore-based Steelmaking process (period: 1900 to 1925 for Pudding, 1900-1975 for Bessemer and Thomas, 1900-2015 for OHF, 1900-1921 for BOF, 1900-1921 for Crucible, and 1900-2015 for EAF)

A significant technical improvement was shown in the steelmaking process, and around six major technologies (i.e. Puddling, Open hearth furnace, Bessemer, Thomas, Crucible, Electric Furnace, and Basic Oxygen furnace) have been applied during the period from 1900 to 2015. The historical share of each technology can be found in Fig. S1.4 [20].

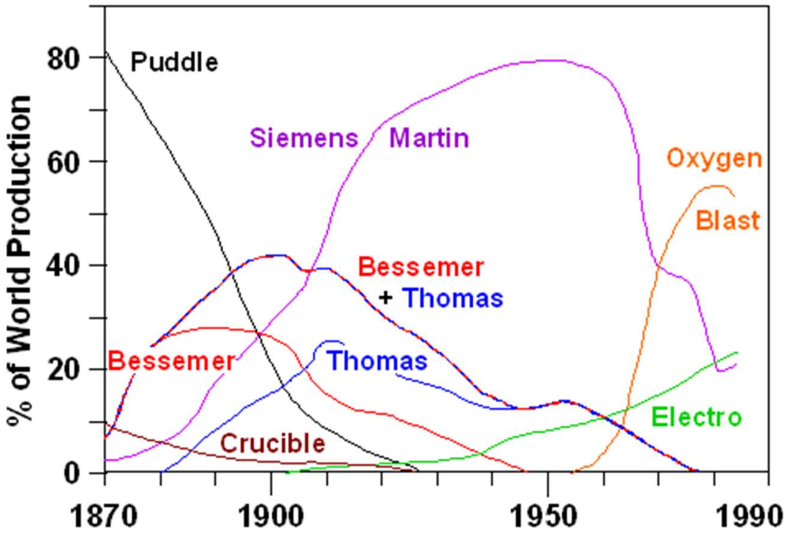

Fig.S1.4 Historical share of steelmaking processes (from [20])

In general, there are three major processes: open-hearth, converter steelmaking, and scrap-based technology. For the open-hearth method, Puddling process was first used to produce puddle iron and mild steel [21]. Meanwhile, the Crucible is one of the oldest processes for melting purpose and fed with wrought iron and coke [22]. Then the open-hearth furnaces (OHF) (i.e. Siemens-Martin process, appeared in 1865) gradually replaced the Puddling process and dominated the entire steelmaking stage [23]. For convert steelmaking, the Bessemer process was one of the most fuel-saving innovations to make inexpensive steel in the early period [24]. However, the Bessemer (and Thomas) process had a bad flexibility and control in steel quality [25], which was gradually replaced by open hearth technology. Then the development of Basic oxygen furnaces (BOF) in convert steelmaking become to dominate the steel making process due to the development of a method to separate oxygen from nitrogen on an industrial scale since the 1960s [26]. For the scrap-based technology, Electric arc furnace (EAF) is the major technique to produce steel at present, which was firstly introduced in the late nineteenth century [10]. Accordingly, the application period of Pudding, Bessemer and Thomas, OHF, BOF, Crucible, and EAF is assumed to be 1900-1925, 1900-1975, 1900-2015, 1955-2015, 1900-1921, and 1900-2015, respectively.

(6) Iron Foundry process (period: 1900 to 2015)

Iron Foundry (IF) is assumed as one stage to include a series of process from iron melting, molding, casting, etc. to produce the cast iron products. It has a long history and still is applied at present [2]. Hence, its application period is assumed to be 1900-2015 in this study.

(7) Steel casting process (period: 1955-2015 for Continuous casting, and 1900-2015 for Ingot casting)

Steel castings are used to deliver required strength or shock resistance to crude steel for subsequent rolling in the finishing mills. There are two main techniques in this stage: ingot casting and continuous casting (with liquid steel for castings). Continuous casting (CC) was introduced in the 1950s and has occupied this stage by 96.2% in 2015. Before the introduction of Continuous casting, the non-continuous casting was the primary technology to produce the cast steel product [27]. The division of these two techniques from 1955 to 2015 is obtained from [27] and world steel yearbook [28].

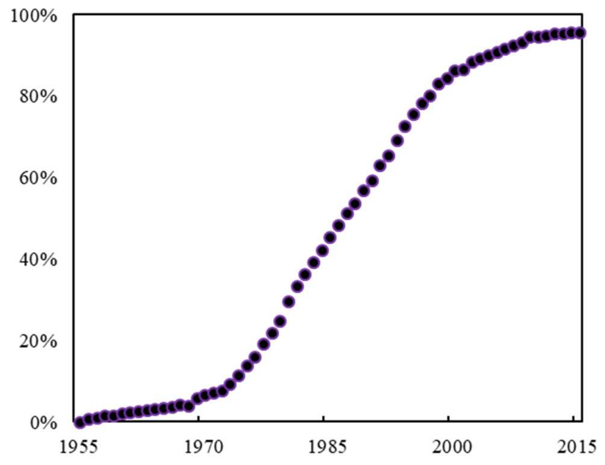

Fig. S1.5 The historical share of continuous casting in the steel cast stage (based on [27,28])

(8) Steel finishing process (period: 1900-2015)

The steel finishing stage is to produce final steel products with various required shapes and properties, which includes a series of process like reheating, forming, shaping, drilling, welding, galvanizing. The rolling and finishing processes have been applied for a long history. For instance, the cold and hot rolling processes can be traced back to the 14th and 17th century, respectively. Hence, the application periods of those processes are all assumed to be 1900-2015 in this study. Due to the lack of statistical data regarding the production from different processes, this study assumes the history of finishing process would follow the contemporary structure as mapped in [2]. Basically, the steel from ingot casting would enter hot rolling mills or become the cast steel for direct use. Meanwhile, the steel from continuous casting would then enter hot rolling mills for reheating and rolling. The hot rolling mills include Section mill, Rod and Bar mill, Plate mill, and Hot strip mill. For simplicity, the cold rolling and finishing stage is treated as one process to produce final cold rolling (CR) products in this study.

S2. Quantitative Method

This section gives the detailed step for the material flow analysis and its associated emission calculation.

S2.1 Material Flow Quantification

This part aims to quantify the stocks, flows, losses within an anthropogenic cycle of steel annually from 1900 to 2015. The part begins with the anthropogenic cycle construction, which follows the basic life cycle stages (i.e. production, manufacturing, in-use, and end-of-life). Several sub-stages are given for each stage. Especially, in the production stage, more detailed sub-stages are given according to the production. For the rest three stages (i.e. manufacturing, in-use and end-of-life stages), their stocks, inflows, and outflows can be obtained based on four major groups of steel products (i.e. construction, transportation, machinery, and durable daily goods). Based on the method of dynamic material flow, the inflow and outflow for each production technology are obtained, and the entire anthropogenic cycle of steel can be quantified.

(1) Production activities data sets

As shown in Figure S2.1, there are 22 processes (in the blocks) in the material production system. Material flow analysis is applied as the primary approach to obtain the mass inflow, outflow, or loss for each process, which all are converted to iron (ferrous) content based on the ratio in the reference [29]. The detailed calculation for each process can be found in Table S2.1. In general, the mass balance principle is applied to determine the resource efficiency, outflow, inflow and losses of each unit process, and the relationship of those four parameters is shown in Fig. S2.1. Given two parameters, the other two parameters for a unit process can be obtained based on the principle of mass balance. Notably, for the application of the mass balance approach, some input parameters should be given exogenously through four approaches (i.e. statistical data, technology analysis, or mass flow allocation), which are marked in Table S2.1. Herein, the technical analysis refers to a literature investigation on the technical features (e.g. yield rate, energy use, energy sources) of the studied technology based on the related technical reports, patent documents, or publications at that time.

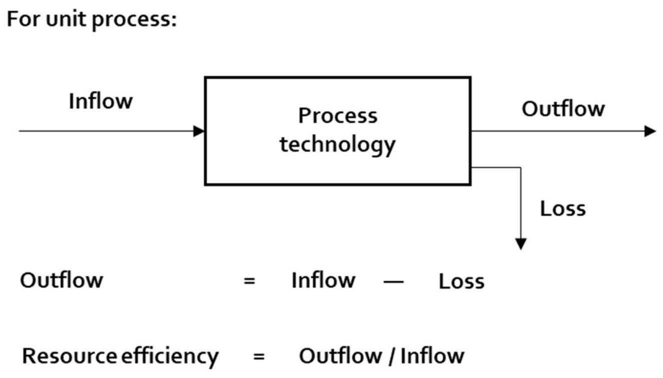

Fig. S2.1 Key parameters of unit process

Table S2.1 Calculation information for each flow in the material production system

Process	Period	Parameter	Equation	Method	Description and reference
1. Total Mining (MI)	1900-2015	MI. Inflow	MI. Outflow / MI.RE	Mass balance	Historical extracted resource
		MI. Resource efficiency (RE)	Exogenously given	Technical Analysis	Based on the relationship of RE with ore grade, which is further explained in Fig.S2.2.
		MI. Outflow	Exogenously given	Statistics based	Fe content: 0.625. The data for 1900-1904 and 1905-2015 is from [30], and USGS [13], respectively.
		MI. Loss	MI. Inflow- MI. Outflow	Mass balance	Iron ore tailing, waste rock.
2.1 Ore Preparation-Sintering	1900-2015	OP1. Inflow	OP1. Outflow × OP1. RE	Mass balance	Mass balance equation
		OP1. RE	Same with BF. RE	Mass balance	Assumed to be same with BF.RE
		OP1. Outflow	OP1.share × BF. Inflow	Statistics based	Data for OP1.share from [12]
		OP1. Loss	OP1. Inflow - OP1. Outflow	Mass balance	Mass balance equation
2.2 Ore Preparation-pelleting	1950-2015	OP1. Inflow	OP2. Outflow × OP2. RE	Mass balance	Mass balance equation
		OP1. RE	Same with BF. RE	Mass balance	Assumed to be same with BF.RE
		OP1. Outflow	(1-OP1.share) × BF. Inflow + DR. Inflow	Mass balance	Based on the inflow from 4.BF
		OP1. Loss	OP2. Inflow – OP2. Outflow	Mass balance	Mass balance equation
3. Direct Reduction (DR)	1970-2015	DR. Inflow	DR. Outflow/ DR. RE	Mass balance	Mass balance equation
		DR. RE	Exogenously given	Technical Analysis	The resource efficiency for Midrex [31], HYL/Energiron [32], and Coal-based Rotary Kiln [33] is 90.66%, 94.3%, and 91.7%, respectively. Those current level is applied for the historical trend.
		DR. Outflow	Exogenously given	Statistics based	Fe content: 0.835; Determined by parameters of four processes from [19]
		DR Loss	DR. Inflow- DR. Outflow	Mass balance	Mass balance
4. Blast Furnace (BF)	1900-2015	BF. Inflow	BF. Outflow / BF. RE	Mass balance	Mass balance
		BF. RE	(BF.outflow /(MI.outflow - DR. Inflow))^0.5	Statistics based	Assumed to be same with sintering and pelletizing, based on pig iron outflow and mining production
		BF. Outflow	Exogenously given	Statistics based	Fe content: 0.94, Pig iron production data is from [13,28].
		BF Loss	BF. Inflow- BF. Outflow	Mass balance	Mass balance
5. Iron Foundry	1900-2015	IF. Inflow	BF. Outflow × IF. Share	Mass balance	Mass balance

Process	Period	Parameter	Equation	Method	Description and reference
and Cast		IF. RE	IF. Inflow / IF. Outflow	Mass balance	Mass balance
		IF. Outflow	Exogenously given	Statistics based	Production data from Census of World Casting Production.
		IF Loss	IF. Inflow - IF. Outflow	Mass balance	Mass balance
6.Steelmaking1. Puddling process	1900-1925	SM1. Inflow	SM 1. Outflow/ SM 1. RE	Mass balance	Mass balance
		SM 1. RE	Exogenously given	Technical Analysis	Constant data [21] for this period
		SM 1. Outflow	Exogenously given	Statistics based	Data is from [23,34]
		SM1. Loss	SM1. Inflow- SM1. Outflow	Mass balance	Mass balance
6.Steelmaking2. OHF	1900-2015	SM2. Inflow	(1- OHF. scrap rate) × (SM2. Outflow / SM2. RE)	Mass balance	Mass balance
		SM2. Scrap	OHF. scrap rate × (SM2. Outflow / SM2. RE)	Mass balance	The ratio of scrap in total inflow is assumed to be 45% for the period after 1955 based on USGS mineral yearbook
		SM2. RE	Exogenously given	Technical Analysis	Based on China's case in 1966 [35] and global case in 2008 [36]
		SM2. Outflow	Exogenously given	Statistics based	Data is from the world steel yearbook and [23,34].
		SM2. Loss	SM2. Inflow + SM2. scrap - SM2. Outflow	Mass balance	Mass balance
6. Steelmaking3. Bessemer & Thomas	1900-1975	SM3. Inflow	0.95 × (SM3. Outflow / SM3. RE)	Mass balance	Mass balance
		SM3. Scrap	0.05 × (SM3. Outflow / SM3. RE)	Mass balance	The ratio of scrap in total inflow is assumed to be 5% based on USGS mineral yearbook
		SM3. RE	Exogenously given	Technical Analysis	The resource efficiency of Bessemer process was similar with open hearth furnace [37]
		SM3. Outflow	Exogenously given	Statistics based	Data is from the world steel yearbook and [23,34].
		SM3. Loss	SM3. Inflow + SM3. Scrap – SM3. Outflow	Mass balance	Mass balance
6. Steelmaking4. BOF	1955-2015	SM4. Inflow	(1- BOF. scrap rate) × (SM4. Outflow / SM4. RE)	Mass balance	Mass balance
		SM4. Scrap	BOF. scrap rate × (SM4. Outflow / SM4. RE)	Mass balance	The ratio of scrap in total inflow is based on mass balance and the BF output
		SM4. RE	Exogenously given	Technical Analysis	Based on [38]
		SM4. Outflow	Exogenously given	Statistics based	Data is from [23,28,34].
		SM4. Loss	SM4. Inflow + SM4. Scrap - SM4. Outflow	Mass balance	Mass balance

Process	Period	Parameter	Equation	Method	Description and reference
6.Steelmaking5. EAF	1900-2015	SM5. Inflow	SM5. Outflow/ SM5. RE	Mass balance	From scrap supply
		SM5. RE	Exogenously given	Technical Analysis	See Table S3
		SM5. Outflow	Exogenously given	Statistics based	Data is from [23,28,34].
		SM5. Loss	SM5. Inflow- SM5. Outflow	Mass balance	Mass balance
6. Steelmaking7. Crucible	1900-1920	SM7. Inflow	SM7. Outflow/ SM7. RE	Mass balance	From scrap supply
		SM7. RE	Exogenously given	Technical Analysis	It increased from 77% in 1950 (same with OHF) to 88.9% in 2008 [2]
		SM7. Outflow	Exogenously given	Statistics based	Data is from [23,34].
		SM7. Loss	SM7. Inflow- SM7. Outflow	Mass balance	Mass balance
**Share**	1900-2015	IC. share	Exogenously given	Statistics based	Data from [27,28]
		CC. share	Exogenously given	Statistics based	Data from [27,28]
7. IC	1900-2015	IC. Inflow	Total steel × IC. share	Mass balance	Mass balance
		IC. RE	Exogenously given	Technical Analysis	Follow the same trend with CC. RE but its amount is 13% less [39]
		IC. Outflow	IC. Inflow× IC. RE	Mass balance	Mass balance
		IC. Loss	IC. Inflow - IC. Outflow	Mass balance	Mass balance
8. CC	1955-2015	CC. Inflow	Total steel × CC. share	Mass balance	Mass balance
		CC. RE	Exogenously given	Technical Analysis	Data from [40]
		CC. Outflow	CC. Inflow× CC. RE	Mass balance	Mass balance
		CC. Loss	CC. Inflow - CC. Outflow	Mass balance	Mass balance
Cast steel	1900-2015	IC. Outflow × Cast steel. share			Cast steel. share is assumed to be 12.1% [2]
9.1 Section Mill	1900-2015	SeM. Inflow	IC. Outflow × ISeM. share + CC. Outflow × CSeM. share	Mass balance	Statistics based for SeM. share, which is further explained.
		SeM. RE	Exogenously given	Technical Analysis	Data from [2]
		SeM. Outflow	SeM. Inflow× SeM. RE	Mass balance	Mass balance
		SeM. Loss	SeM. Inflow - SeM. Outflow	Mass balance	Mass balance
9.2 Rod and Bar Mill	1900-2015	RbM. Inflow	IC. Outflow × IRbM. share + CC. Outflow × CRbM. share	Mass balance	Statistics based for SeM. share, which is further explained.

Process	Period	Parameter	Equation	Method	Description and reference
		RbM. RE	Exogenously given	Technical Analysis	Data from [2]
		RbM. Outflow	RbM. Inflow× RbM. RE	Mass balance	Mass balance
		RbM M. Loss	RbM. Inflow - RbM. Outflow	Mass balance	Mass balance
9.3 Plate Mill	1900-2015	PtM. Inflow	IC. Outflow × IPtM. share + CC. Outflow × CPtM. share	Mass balance	Follow the allocation form [2], which is further explained.
		SeM. RE	Exogenously given	Technical Analysis	See Table S3
		SeM. Outflow	PtM. Inflow × PtM. RE	Mass balance	Mass balance
		SeM. Loss	PtM. Inflow - PtM.Outflow	Mass balance	Mass balance
9.4 Strip Mill	1900-2015	StM. Inflow	IC. Outflow × IStM. share + CC. Outflow × CStM. share	Mass balance	Follow the allocation form [2] , which is further explained.
		StM. RE	Exogenously given	Technical Analysis	Data from [2]
		StM. Outflow	StM. Inflow× StM. RE	Mass balance	Mass balance
		StM. Loss	StM. Inflow - StM. Outflow	Mass balance	Mass balance
9.5 Cold rolling and finishing	1900-2015	CRF. Inflow	RdM. Outflow× RdM. share+ StM. Outflow× StM. share	Mass balance	Mass balance
		CRF. RE	Exogenously given	Technical Analysis	Data from [2]
		CRF. Outflow	CRF. Inflow× CRF. RE	Mass balance	Mass balance
		CRF. Loss	CRF. Inflow - CRF.Outflow	Mass balance	Mass balance

Note: the world steel yearbook data [28] represents all yearbook from 1978 to 2017 in the official website of world steel association.

As listed in Table S2.1, the data sources for mining RE and mass allocation in rolling and finishing stage should be further clarified as follows:

(a) Mining RE. There is no statistical data regarding the RE of mining. Herein, its historical trend is estimated by the following steps: Firstly, as shown in Fig. S2.2A, this study collected the RE data for different countries in 2000 from [4], and its ore grade data from USGS mineral yearbook. It is found that there is a high correlation between the ore grade and RE. The second step is to obtain the historical ore grade trend, which is also a lack of statistical data. Hence, based on the investigation from [41] for the period from 1905 to 1925 and study [42] for the period from 2000 to 2015. The estimated trend of ore grade is shown in Fig. S2.2C. Finally, as shown in Fig. S2.2D, the historical trend of RE can be obtained based on the ore grade trend and its relationship with RE.

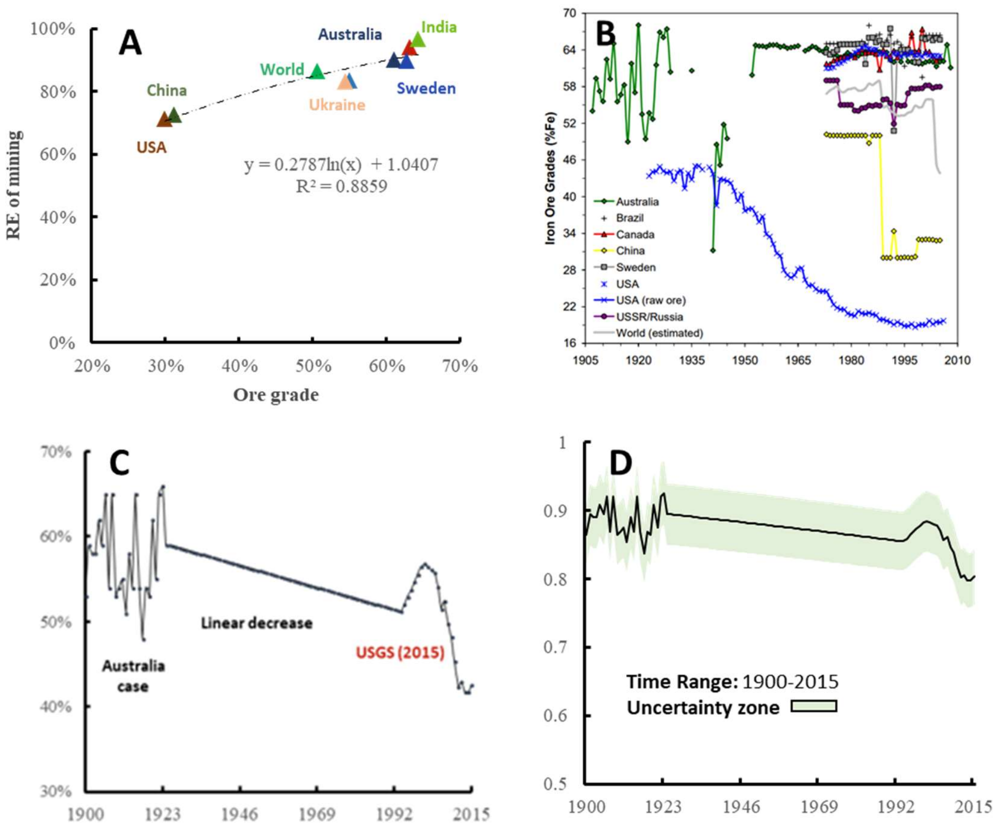

Fig. S2.2 Data for RE mining estimation (A. Relationship of RE and ore grade, B. Historical data of global iron ore grade [41], C. Historical change of global iron ore grade, and D. the estimated change of mining RE between 1900 and 2015. Shaded green area indicate an uncertainty range of ±7%)

(b) Steel product mass allocation. This study quantify the steel flows from casting to end-use sectors based on the studies from [2], the relationship of each flow is mapped in Fig.S2.3A (the red figure represents the material flow as noted in Fig. S2.3A). This study assumed all the cold rolling and finishing stage as one process to produce final cold rolling (CR) products, and the production flow from ingot casting (i.e. AA) is allocated to five downstream processes (i.e. 12.1% into cast steel, 6% into section mill, 36.1% into rod/bar mill, 8% into plate mill, and 16.9% into strip mill). For the share of outflow from Rod/Bar Mill and Strip Mill to the CR process, it is based on the historical data from world steel yearbook from 1984 to 2015 and United States case [43] from 1942 to 1984. Meanwhile, the allocation of final steel products (i.e. A-caste steel, B-shapes, C-bars, D-cold-rolling products, E-plates, F-coil and strip, G-cast iron) to four end-use applications (i.e. construction, vehicles, machinery, daily goods) is quantified based on the allocation matrix in [2], which is marked as red in the left box of Fig.S2.3A (i.e. inflow to construction=0.99*N+0.6*C+0.36*D+0.06*E+0.62*F+0.4*G).

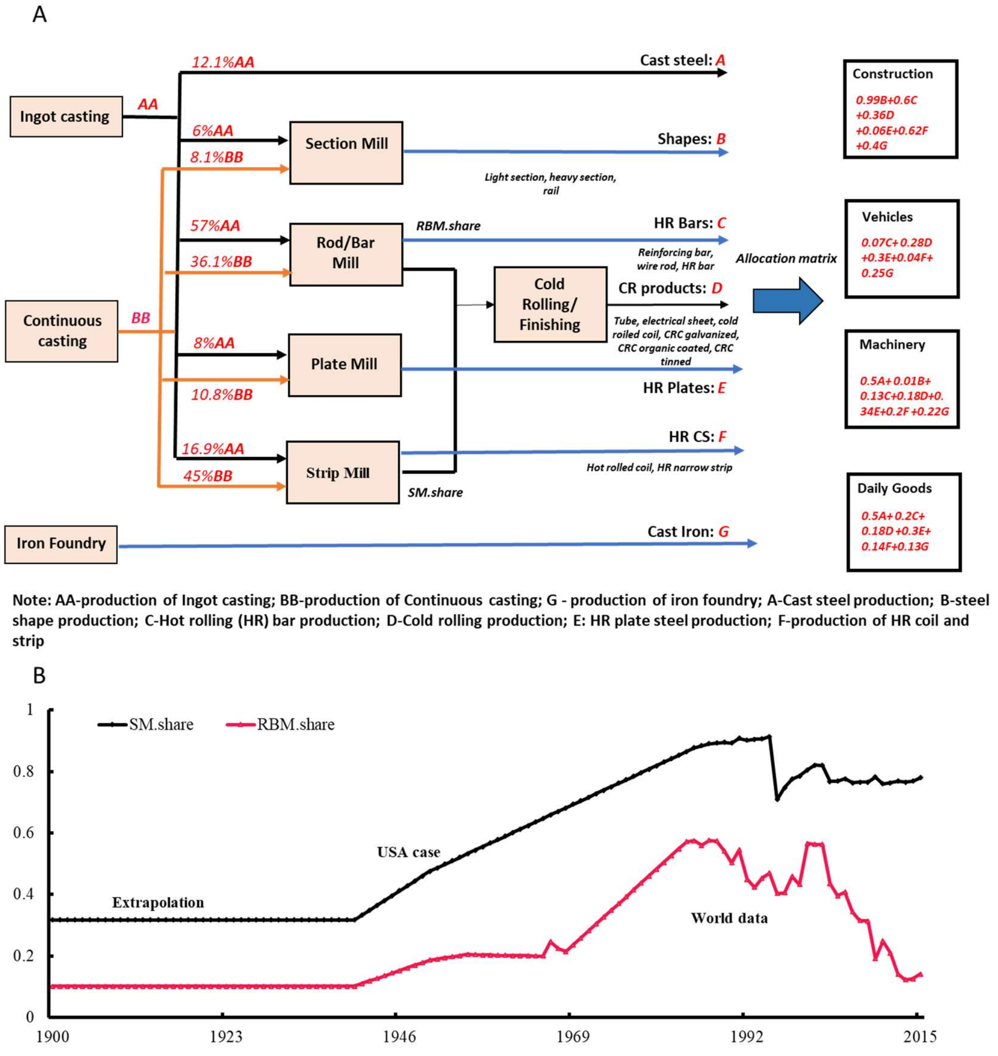

Fig. S2.3 Steel allocation model (A: Mass allocation from rolling to finishing [2]; B. The historical change of mass share to cold rolling)

(2) Fabrication, In-use and End-of-life stage

Those three stages are treated herein as they are more application-specific rather than material-specific as in the material supply system. In link with other steel MFA studies [3], the end-use applications are mainly divided into four sectors: Construction, Vehicles, Machinery, and Daily goods. The corresponding parameters, like market share, lifetime, and resource efficiency, are specific to each other.

(a) Fabrication. The product fabrication stage transfers the raw materials from either primary or secondary production into different products for use. For the material in sector *n*, the inflow is determined by the allocation model from [2]. Meanwhile, the fabrication rate (FR) are assumed to follow the current level from [2], which is shown in Table S2.2. The scrap from fabrication is named promote scrap, which is assumed to be fully recycled and treated in the secondary production stage.

Table S2.2 Parameters to estimate stocks and flows in Fabrication, In-use and End-of-life stage

	Construction	Transportation	Machinery	Metal goods
Market share	Determined by the allocation model			
Fabrication rate-2008	93% (±20%)	73% (±20%)	83% (±20%)	77% (±20%)
Lifetime (year)	62.5(±26%)	16.65(±20%)	25(±20%)	12.5(±20%)

(b) In-use Stage. The products from fabrication stage finally enter its corresponding in-use stage to provide services as in-use stock. The inflow into the in-use stock equals the final products from fabrication stage. As for the outflow, it follows the lifetime distribution scheme [3]:

$$\mathrm{Outflow}(T,p) = \frac{\mathrm{Stock}(T_0)}{T} + \int_{T_0}^{T} \mathrm{Inflow}(t) \times f(t,\tau,\sigma)dt \quad \text{S1}$$

$$f(t,\tau,\sigma) = \frac{1}{\sigma\sqrt{2\pi}} \times exp(\frac{-(t-T_0-\tau)^2}{2\sigma^2}) \quad \text{S2}$$

where, $f(t,\tau,\sigma)$ is the probability density of the lifetime distribution function; t is the quantification time step; τ is the lifetime of this product sector; σ is the standard deviation of lifetime; T is the end of the studied period; T_0 is the starting time. The $Stock(T_0)$ is the initial in-use stock in the year 1900 based on the study from [44]. The lifetime distribution could be Normal, Delta or Weibull distribution, and the normal distribution and its lifetime is adopted from [3,29] in this study.

The specific parameters for fabrication rate are obtained from [3,29]. Meanwhile, 10% of outflow in the construction sector is assumed to be accumulated as obsolete stock, which refers to the products which are out of service and not accessible for recycling [3,29,45]. The basic settings for those parameters are shown in Table S3.

(c) Scrap, Recycling and Losses. Three types of scrap (i.e. home, new and old scrap) are generated from the anthropogenic iron cycle. As shown in Fig. S2.4, this study follows the conventional definitions of those scraps [46]: home scrap refers to scrap generated from casting, or foundry production, which can be internally recycled inside the material product sites. New scrap (or prompt scrap) refers to the scrap generated from during the fabrication of metal products, which is transferred to the scrap market for further recycling. Old scrap (or EoL scrap, postconsumer scrap) refers to the scrap generated from the products, which enters to the end of their service life.

The recycling stage includes a series of processes, e.g. collection, sorting, and separation. According to the [46], the end-of-life recycling rate (EoL-RR) equals to the recycled Old-scrap(*c2* in Figure S2.4) divided by the old scrap generation. Herein, the old scrap generation equals to the outflow in the equation S1, and the new scrap recycling rate is assumed to be same with EoL RR. Based on mass balance, the recycled new and old scrap can be obtained. Furthermore, this study also quantifies the annual resource loss from each life cycle stage, as shown in Fig. S2.4.

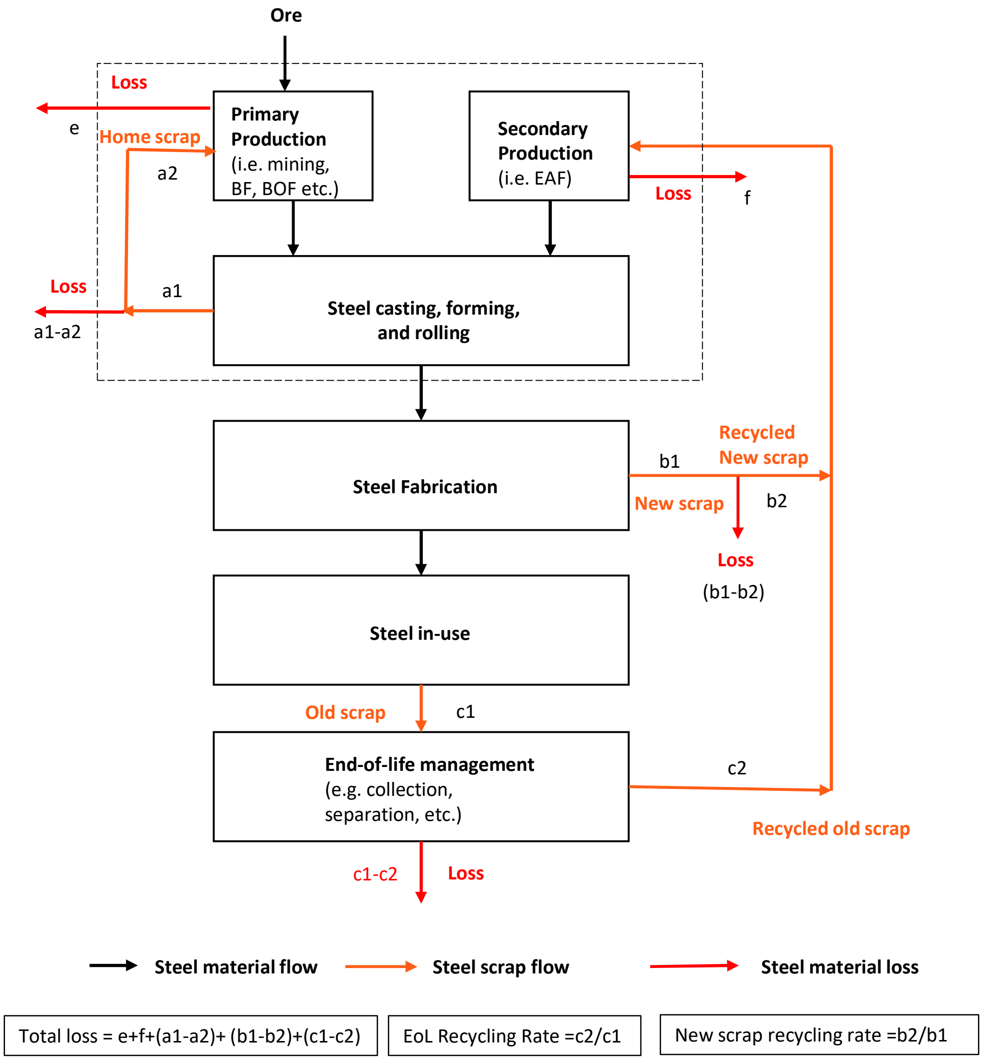

Fig. S2.4 Scrap and resource losses during steel life cycle

S2.2 GHG Emission Calculation

A bottom-up approach was chosen for quantifying emissions of greenhouse gases (GHG) from steel production. The benefit of the bottom-up approach is that it allows for disaggregating steel production into different steel production processes. (e.g., mining, blast furnace, cold rolling, etc.). This allows for modelling and expressing the technological development of the different processes between 1900 and 2015. Such specific differentiation cannot be obtained using top-down approaches, such as environmentally extended input-output databases.

The bottom-up approach was modelled using the life-cycle assessment (LCA) software SimaPro version 9.0.0.35. General activities in the steel processing sector were modelled using information from the Ecoinvent life-cycle inventory database [47] coupled with specific process information as described in Section S2.1 to obtain a comprehensive overview of the processes involved in steel processing between 1900 and 2015.

The coupling with information from Section S2.1 is particularly important for processes, which are not used anymore and, not covered in the ecoinvent database that primarily covers processes that are currently part of the steel processing industry. Because the information given in Section S2.1 is generally for specific inputs and outputs of the process (e.g. electricity or coke use), process specific details, such as direct carbon emissions associated with running the process, were not available (e.g. CO_2 emissions as a result of carbon removal from the iron ore and generation of residual waste for treatment). For this, we used the available process information from ecoinvent [47] and extrapolated this to processes with a similar function in the steel processing value chain, in order to construct more complete unit processes for all activities involved in steel processing which include both direct and indirect emissions and resource uses.

A full presentation of the life-cycle inventory used for modelling the 19 steel processing activities is given in Supplementary Data 1. This includes all direct emissions and resources uses for each process as well as all process inputs required for the functioning of the steel processing process, such as electricity generation, coke production, oxygen production, etc.

Herein, several essential information regarding this historical calculation is introduced as follows:

S2.2.1 Historical trajectory of energy efficiency

The statistical data related to the annual energy use of each studied production processes are not available. This study estimated the annual energy inventory of each technology based on the typical value and their trajectories in the history, which are obtained in two steps: firstly, this study collected the trend of energy use from various published studies and presented in the Fig. S2.5, where the key reference is summarized in Table S2.3. Notably, the BF and EAF have continuous inventory data from 1900, and 1965, to 2010, respectively. Secondly, the data for the rest of the time and processes is estimated based on the typical energy use estimates [10–14,48–52] and the trajectory in Fig. S2.5c.

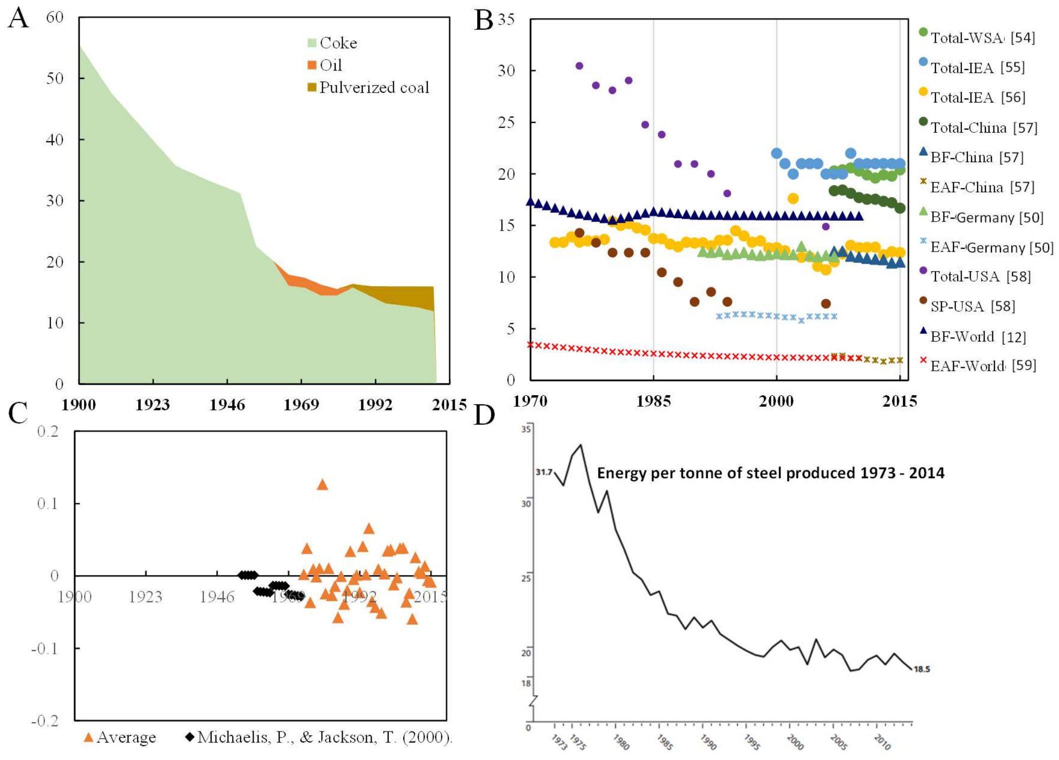

Fig. S2.5 The energy use trend of steel production from published studies (Note: Unit: GJ/t; **A**: The annual energy inventory of blast furnace from 1900 to 2010; **B**. The annual energy use trend of total steel production, EAF, and BOF from published studies; The references for the series in this figure are given in the squared brackets and from [53], [54], [55], [56], [50], [57], [12] and [58]. C. The annual change of total energy efficiency of steel production, in which the average refers to the data from publications in B; and D. Energy use per tonne of steel produced from 1973 to 2014[59])

Table S2.3 List of key reference for energy input of key production processes

Production technology	Period	Reference and note
Mining	1900-2015	See section S2.1(a)
Sintering	1900-2015	Benchmark value [60]<br>The historical trend follows the average energy efficiency trend [38,50]
Pelleting. EI	1950-2015	Benchmark value from a steel plant in China [61]<br>The historical trend follows the average energy efficiency trend [38]
DRI. EI	1980-2015	Midrex [31], HYL/Energiron [32], and Coal-based Rotary Kiln [33]
BF. EI	1900-2015	The historical trend was investigated in [8,50]
PD. EI	1900-1925	The benchmark value is from [21]<br>This historical trend (25 years) remains at this level
OHF. EI	1900-2015	The benchmark value is from [62]<br>The historical trend was investigated in [38]
BM. EI	1900-1975	The benchmark value is from [63]<br>The historical trend follows the OHF case [38]
BOF. EI	1950-2015	The benchmark value and historical trend was investigated in [38,50]
IF. EI	1900-2015	The benchmark value is from [64]<br>The historical trend follows the OHF case [38]
CB. EI	1900-1920	The benchmark value is from [10]. This historical trend (20 years) remains at this level
EAF. EI	1900-2015	The benchmark value and historical change was investigated in [10,50]
IC. EI	1900-2015	The benchmark value and historical trend was investigated in [38,48]
CC. EI	1950-2015	The benchmark value and historical trend was investigated in [48,65]
SeM. EI	1900-2015	Benchmark value from [48]<br>The historical trend follows the average energy efficiency trend [38,50]
PtM. EI	1900-2015	Benchmark value from [48]<br>The historical trend follows the average energy efficiency trend [38,50]
StM. EI	1900-2015	Benchmark value from [48]<br>The historical trend follows the average energy efficiency trend [38,50]
RbM. EI	1900-2015	Benchmark value from<br>The historical trend follows the average energy efficiency trend [38,50]
CdM. EI	1900-2015	Benchmark value from<br>The historical trend follows the average energy efficiency trend [38,50]

S2.2.2 System boundary of LCA for each technology

In this section, the system boundary for of each studied technology is illustrated in Fig.S2.6-S2.15. The system boundaries indicate the main processes as well as inputs and outputs from auxiliary processes. Hereby, Fig.S2.6-S2.15 provide an overview of the processes covered and used for quantifying the technology specific GHG emissions for each studied technology. The system boundaries are divided into a foreground and a background system. The foreground system includes technology specific processes for which specific modelling choices have been made about the process, such as historical development of the technology. This is presented in Section S2.1 and S2.2. The background system includes more generic unit processes from ecoinvent 3.1 that are used as inputs for processes in the foreground system. Please see Supporting Material 2 for a full overview of the life-cycle inventory and processes used for modelling each of the 19 studied processes covered in this study.

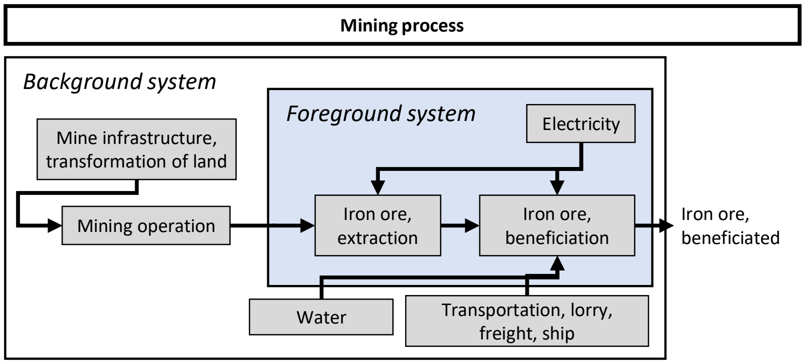

Fig. S2.6 System boundary for GHG emission quantification of mining technology (process hereafter)

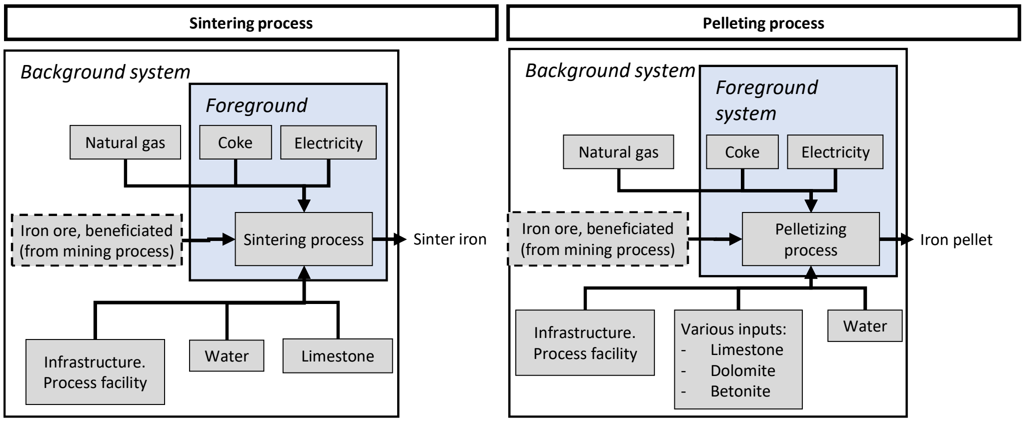

Fig. S2.7 System boundary for GHG emission quantification of sintering process and pelleting process.
(Upstream processes in the steel life-cycle are indicated with dotted line borders. GHG emissions pertaining to such upstream processes is taken into account in the system boundary for that particular process and, thus, not included in the GHG emission quantification for subsequent downstream processes.)

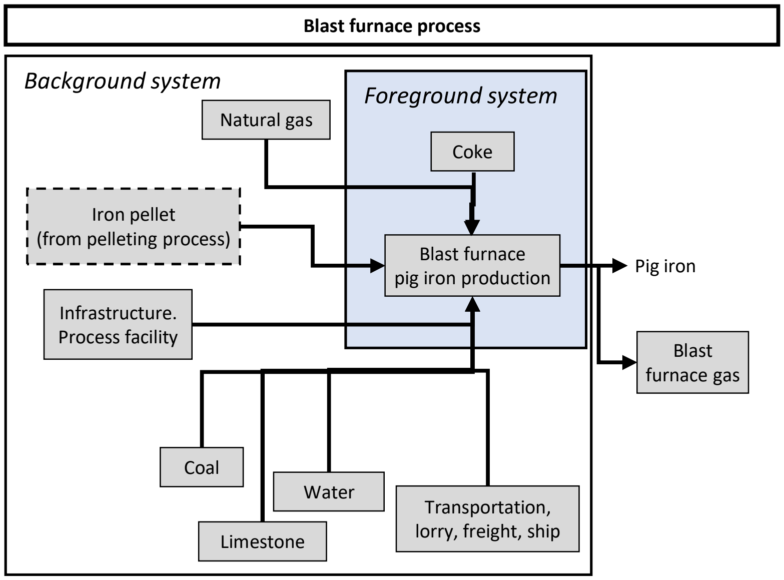

Fig. S2.8 System boundary for GHG emission quantification of blast furnace process. (Upstream processes in the steel life-cycle are indicated with dotted line borders. GHG emissions pertaining to such upstream processes is taken into account in the system boundary for that particular process and, thus, not included in the GHG emission quantification for subsequent downstream processes.)

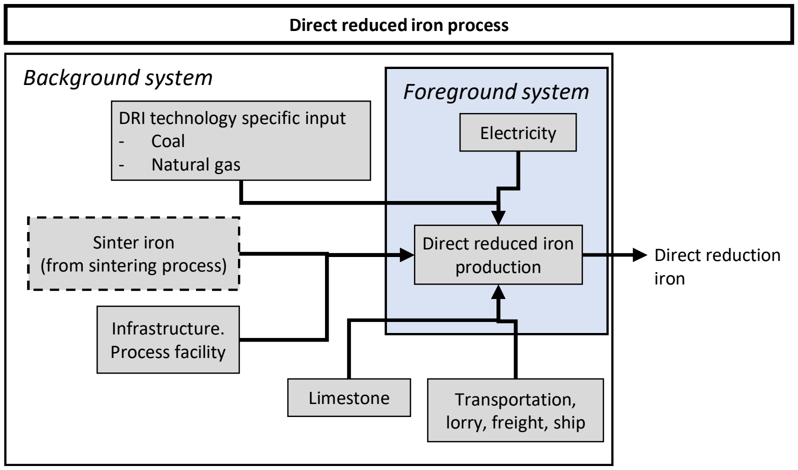

Fig. S2.9 System boundary for GHG emission quantification of Direct Reduction Iron (DRI) production process. (Upstream processes in the steel life-cycle are indicated with dotted line borders. GHG emissions pertaining to such upstream processes is taken into account in the system boundary for that particular process and, thus, not included in the GHG emission quantification for subsequent downstream processes.)

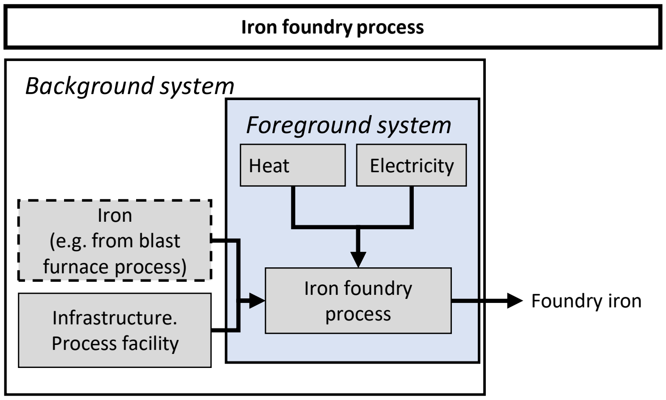

Fig. S2.10 System boundary for GHG emission quantification of iron foundry process. Upstream processes in the steel life-cycle are indicated with dotted line borders. GHG emissions pertaining to such upstream processes is taken into account in the system boundary for that particular process and, thus, not included in the GHG emission quantification for subsequent downstream processes.

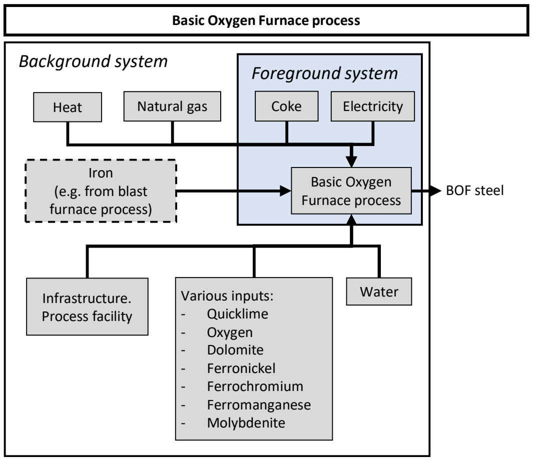

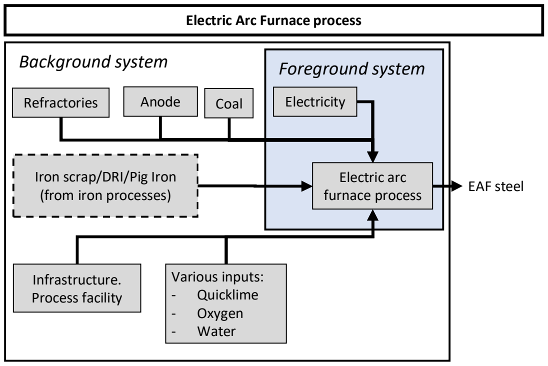

Fig. S2.11 System boundary for GHG emission quantification of Basic Oxygen Furnace (BOF) process and Electric Arc Furnace (EAF) process (Upstream processes in the steel life-cycle are indicated with dotted line borders. GHG emissions pertaining to such upstream processes is taken into account in the system boundary for that particular process and, thus, not included in the GHG emission quantification for subsequent downstream processes.)

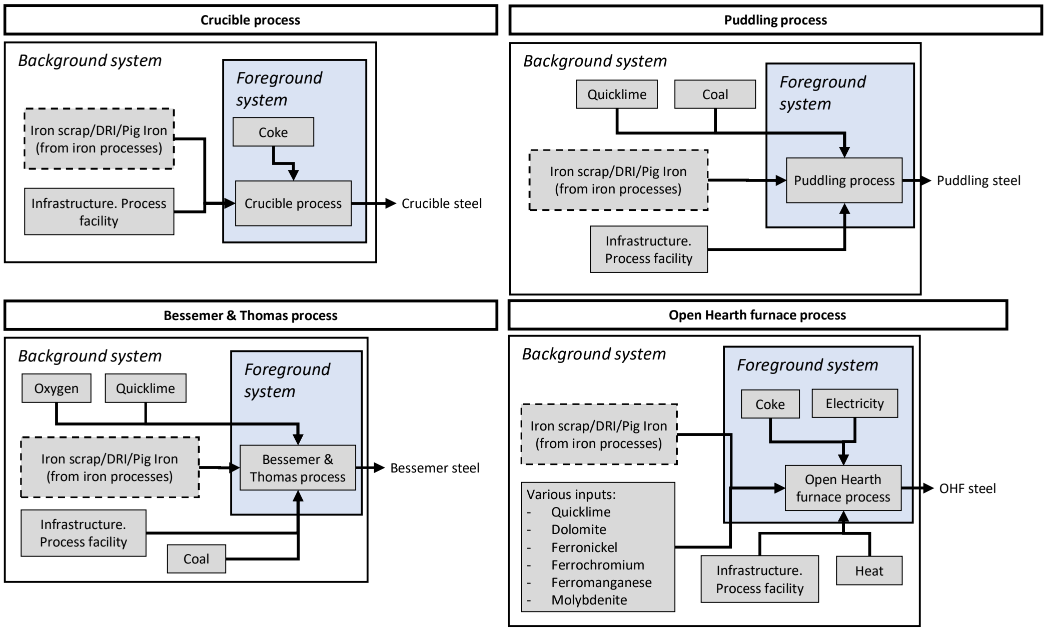

Fig. S2.12 System boundary for GHG emission quantification of Crucible process, Puddling process, Bessemer & Thomas process and Open hearth furnace process. (Upstream processes in the steel life-cycle are indicated with dotted line borders. GHG emissions pertaining to such upstream processes is taken into account in the system boundary for that particular process and, thus, not included in the GHG emission quantification for subsequent downstream processes.)

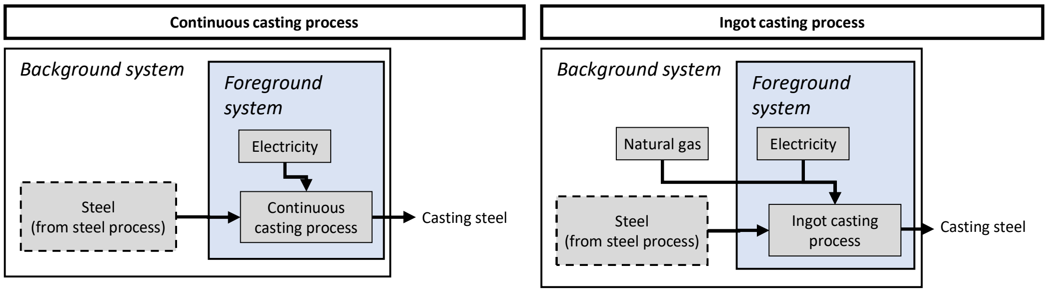

Fig. S2.13 System boundary for GHG emission quantification of Continuous Casting and Ingot casting process. (Upstream processes in the steel life-cycle are indicated with dotted line borders. GHG emissions pertaining to such upstream processes is taken into account in the system boundary for that particular process and, thus, not included in the GHG emission quantification for subsequent downstream processes.)

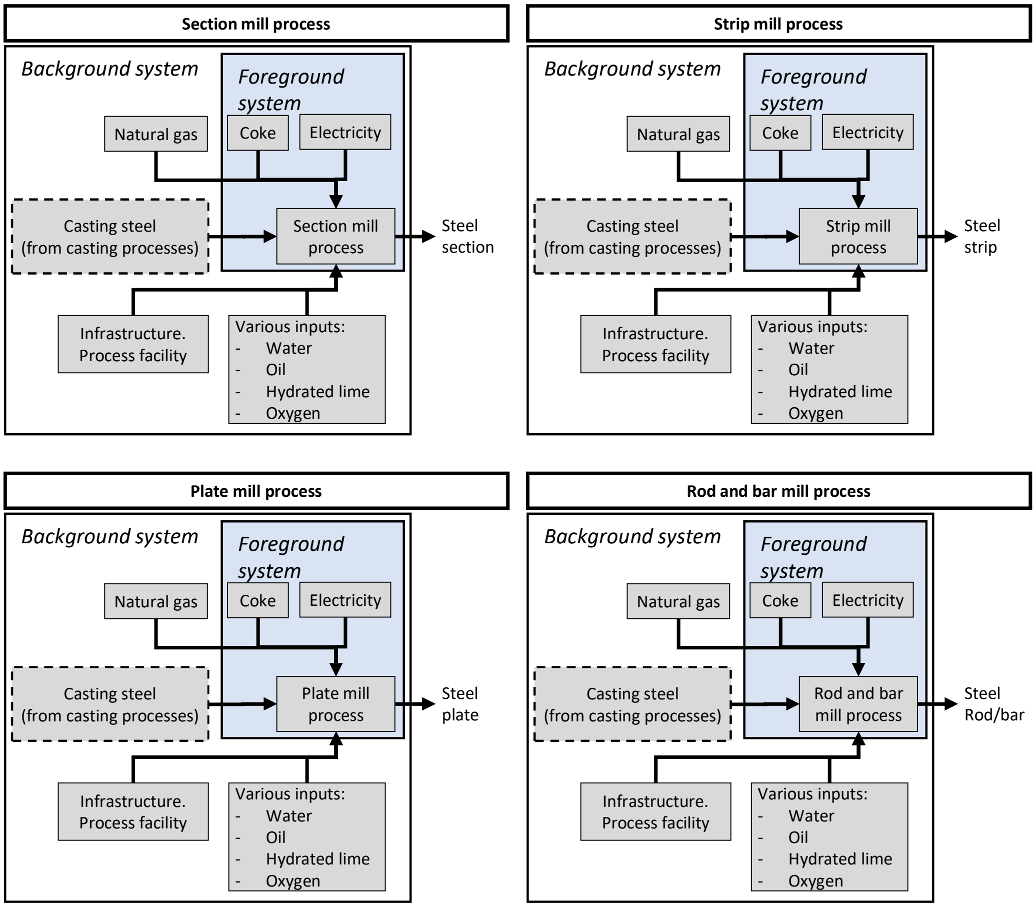

Fig. S2.14 System boundary for GHG emission quantification of Section mill process, Strip mill process, Plate mill process and Rod and bar mill process. (Upstream processes in the steel life-cycle are indicated with dotted line borders. GHG emissions pertaining to such upstream processes is taken into account in the system boundary for that particular process and, thus, not included in the GHG emission quantification for subsequent downstream processes.)

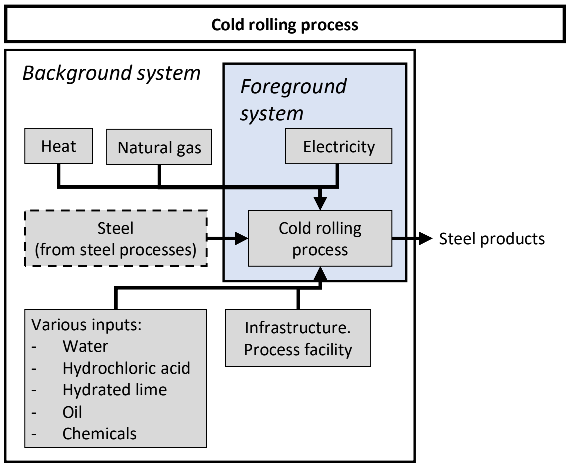

Fig. S2.15 System boundary for GHG emission quantification of Cold casting process. (Upstream processes in the steel life-cycle are indicated with dotted line borders. GHG emissions pertaining to such upstream processes is taken into account in the system boundary for that particular process and, thus, not included in the GHG emission quantification for subsequent downstream processes.)

S2.2.3 GHG emission calculation

Based on the technology and time differentiated inventory of GHG emissions, the total GHG intensity for each steel production technology was estimated according to Eq. S3.

$$E_i(t) = \sum_x E_{x,i}(t) \times GWP100_x \tag{S3}$$

Where E_i is the GHG emission intensity of steel production technology i [kg CO_2-eq / kg output] at year t (see Figure S2.16). $E_{x,i}$ is kg emission of GHG x per kg output from steel production technology i at time t. $GWP100_x$ is the global warming potential [kg CO_2-eq / kg GHG_x emitted] for GHG x [66] (see Table S2.4). The development in total GHG intensity [kg CO_2-eq / process output] over time for each steel processing process is shown in Figure S2.6.

The total GHG emission per steel production technology per year was estimated as:

$$mGHG_i(t) = E_i(t) \times m_i(t) \tag{S4}$$

Where $mGHG_i(t)$ is the total emission of CO_2-eq in year t from steel production technology i. $m_i(t)$ is the total output from steel production technology i at time t estimated using the dynamic MFA model. The sum of all CO_2-eq emissions from all steel production processes in year t gave the total emission of CO_2-eq from steel production in year t:

$$mGHG(t) = \sum_i mGHG_i(t) \tag{S5}$$

Table S2.4 List of GHGs included the assessment and their GWP100

Greenhouse gas	GWP100 [kg CO_2-eq / kg GHG][66]
Carbon dioxide, fossil	1
Methane, fossil	28
Dinitrogen monoxide	265
Sulfur hexafluoride	23507
Methane, chlorodifluoro-, HCFC-22	1765
Carbon dioxide, land transformation	1
Methane, biogenic	25.25
Methane, tetrafluoro-, CFC-14	6626
Ethane, 1,2-dichloro-1,1,2,2-tetrafluoro-, CFC-114	8592
Methane, bromotrifluoro-, Halon 1301	6292
Ethane, hexafluoro-, HFC-116	11123
Methane, tetrachloro-, CFC-10	1728
Ethane, 1,1,2-trichloro-1,2,2-trifluoro-, CFC-113	5824
Methane, bromochlorodifluoro-, Halon 1211	1746
Ethane, 1,1,1,2-tetrafluoro-, HFC-134a	1301
Ethane, 1,1-difluoro-, HFC-152a	138
Ethane, 2-chloro-1,1,1,2-tetrafluoro-, HCFC-124	527
Methane	28
Methane, dichlorodifluoro-, CFC-12	10239
Methane, monochloro-, R-40	12.18
Ethane, 1,1,1-trichloro-, HCFC-140	160
Methane, dichloro-, HCC-30	8.92
Chloroform	16.4
Ethane, 1,2-dichloro-	8.98E-01
Methane, trifluoro-, HFC-23	12398
Methane, trichlorofluoro-, CFC-11	4663
Methane, dichlorofluoro-, HCFC-21	148
Nitrogen fluoride	16070
Methane, bromo-, Halon 1001	2.35

S2.2.3 GHG emission intensity

Fig. S2.6 gives the emission intensity of each studied technology from 1900 to 2015. The uncertainty assessment will be introduced in Section S2.3. GHG emissions from steel processing processes generally decrease over time as the technology is improved, as is reflected in total GHG intensity for steel manufacturing. However, increases in GHG emissions were observed for mining, direct iron reduction, and electric arc furnace (EAF). The increase for mining is due to the increasing need for energy to extract the iron ore from the mines. Changes in GHG intensity for other processes is either due to change in the performance of the technology, e.g., via increase energy or material efficiency, or because the energy grid mix needed for the process changes over time. For instance, a relatively large share of the global electricity mix came from hydropower (22%) in 1980 [67]. In 2000 to 2015, the share of electricity from hydropower was only about 16%, and a proportionally larger share of electricity came from fossil fuels [68]. For this reason, several processes also show an increase in GHG emission intensity from around 2009. This increase is due to an increased share of electricity and heat coming from coal and natural gas. The historical development in electricity and heat generation is shown in Table S2.5 and Table S2.6, respectively.

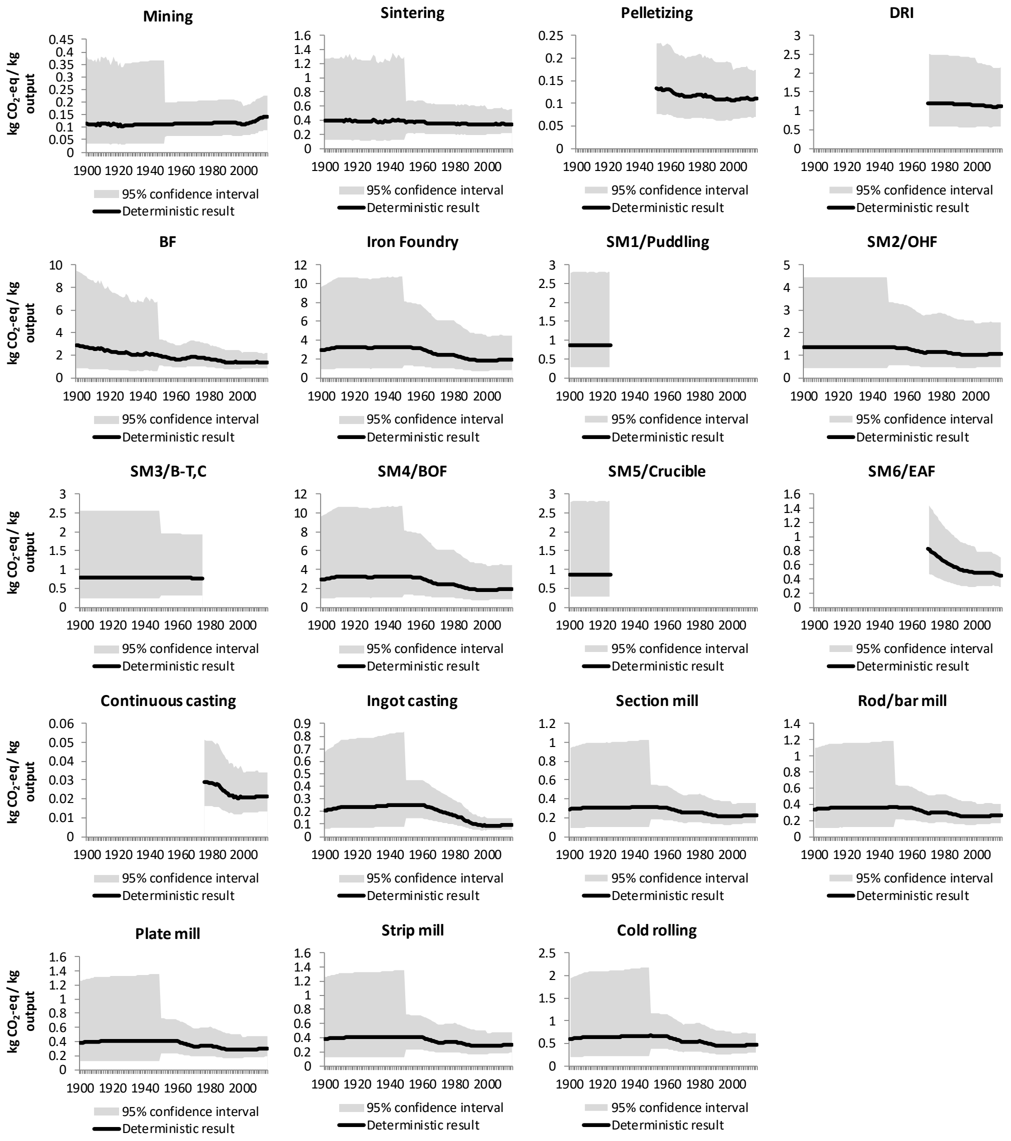

Fig. S2.16. GHG intensity for steel manufacturing processes included in the assessment. Data are presented as the deterministic results and the shaded areas indicate the 95% confidence interval of the estimates.(Note Intensities include direct process emissions and indirect emissions from energy and material inputs necessary for running the process, except emissions related to other steel manufacturing processes also shown in the figure)

Table S2.5 Development over time in distribution of energy carriers for electricity generation. See Supplementary Data 1 for more information about unit processes used to represent electricity generation technology.

Global Electricity mix	1900	1910	1920	1930	1940	1950	1960	1970	1980	1990	2000	2010	2015
	11							69		68			
Oil	0.015	0.025	0.049	0.089	0.118	0.195	0.273	0.214	0.188	0.114	0.080	0.046	0.041
Coal	0.473	0.554	0.547	0.510	0.514	0.451	0.380	0.395	0.392	0.371	0.386	0.402	0.392
Natural gas	0.005	0.009	0.013	0.030	0.039	0.075	0.110	0.123	0.106	0.147	0.177	0.224	0.228
Nuclear	0.000	0.000	0.000	0.000	0.000	0.000	0.000	0.024	0.093	0.169	0.167	0.128	0.106
Biofuel	0.505	0.409	0.387	0.364	0.321	0.268	0.219	0.002	0.003	0.009	0.007	0.013	0.018
Waste incineration	0.000	0.000	0.000	0.000	0.000	0.000	0.000	0.000	0.000	0.002	0.003	0.004	0.004
Hydropower	0.001	0.002	0.004	0.007	0.009	0.012	0.017	0.240	0.217	0.184	0.174	0.164	0.164
Geothermal	0.000	0.000	0.000	0.000	0.000	0.000	0.000	0.000	0.004	0.003	0.003	0.003	0.003
Wind	0.000	0.000	0.000	0.000	0.000	0.000	0.000	0.000	0.000	0.000	0.002	0.016	0.034
Solar thermal	0.000	0.000	0.000	0.000	0.000	0.000	0.000	0.000	0.000	0.000	0.000	0.000	0.000
Photovoltaic	0.000	0.000	0.000	0.000	0.000	0.000	0.000	0.000	0.000	0.000	0.000	0.002	0.010

Table S2.6 Development over time in distribution of energy carriers for heat generation

Global Heat mix	1900	1910	1920	1930	1940	1950	1960	1970	1980	1990	2000	2010	2015
	70									68			
Oil	0.000	0.000	0.000	0.000	0.000	0.050	0.600	0.550	0.350	0.161	0.095	0.058	0.045
Coal	0.980	0.980	0.980	0.980	0.980	0.900	0.300	0.200	0.262	0.306	0.356	0.400	0.448
Natural gas	0.000	0.010	0.010	0.010	0.010	0.050	0.100	0.250	0.370	0.512	0.512	0.482	0.431
Nuclear	0.000	0.000	0.000	0.000	0.000	0.000	0.000	0.000	0.003	0.003	0.002	0.002	0.002
Biofuel	0.020	0.010	0.010	0.010	0.010	0.000	0.000	0.000	0.010	0.011	0.018	0.032	0.040
Waste incineration	0.000	0.000	0.000	0.000	0.000	0.000	0.000	0.000	0.005	0.005	0.016	0.025	0.031
Hydropower	0.000	0.000	0.000	0.000	0.000	0.000	0.000	0.000	0.000	0.000	0.000	0.000	0.000
Geothermal	0.000	0.000	0.000	0.000	0.000	0.000	0.000	0.000	0.000	0.001	0.002	0.002	0.003
Wind	0.000	0.000	0.000	0.000	0.000	0.000	0.000	0.000	0.000	0.000	0.000	0.000	0.000
Solar thermal	0.000	0.000	0.000	0.000	0.000	0.000	0.000	0.000	0.000	0.000	0.000	0.000	0.000
Photovoltaic	0.000	0.000	0.000	0.000	0.000	0.000	0.000	0.000	0.000	0.000	0.000	0.000	0.000

S2.3 Uncertainty analysis

The data sources and uncertainty levels of those exogenously given input parameters are listed in Table S2.7 for material flow quantification and Table 2.8 for greenhouse gas emission quantification.

Table S2.7 Input parameters and their data sources for material flow analysis

Input parameter	Period	Pedigree Matrix Data quality indicator scores	Geometric standard deviation	Reference and note
Mining. outflow	1900-2015	(1,1,1,1,1,1)	1.00	The data for 1900-1904 and 1905-2015 is from [30], and USGS [13], respectively.
Mining. RE	1900-2015	(2,1,1,1,1,2)	1.07	Check S2.1
DRI. outflow	1970-2015	(1,1,1,1,1,1)	1.00	Determined by parameters of four technologies from [19]
DRI. RE	1970-2015	(3,1,1,1,1,1)	1.10	The resource efficiency for Midrex [31], HYL/Energiron [32], and Coal-based Rotary Kiln [33] is 90.66%, 94.3%, and 91.7%, respectively. Those current level is applied for the historical trend.
BF. outflow	1900-2015	(1,1,1,1,1,1)	1.00	From [13,28]
PD. outflow	1900-1925	(1,2,1,1,1,1)	1.02	Data is from [23,34]
PD. RE	1900-1925	(3,3,1,1,1,1)	1.11	Constant data [21] for this period
OHF. outflow	1900-1949	(1,2,1,1,1,1)	1.02	Data is from [23,34].
	1950-2015	(1,1,1,1,1,1)	1.00	Data is from the [28] and [23,34].
OHF. RE	1900-2015	(3,3,1,1,1,1)	1.11	Based on China's case in 1966 [35] and global case in 2008 [36]
OHF. scrap rate	1900-2015	(1,3,1,1,1,1)	1.07	USGS mineral yearbook for steel scrap allocation
BOF. outflow	1950-2015	(1,1,1,1,1,1)	1.00	Data is from the [28] and [23]
BOF. RE	1950-2015	(1,3,2,1,1,1)	1.07	Based on [38]
BOF. scrap rate	1950-2015	(1,3,1,1,1,1)	1.07	Based on mass balance of iron and BOF output
Bessemer. outflow	1900-1949	(1,2,1,1,1,1)	1.02	Data is from [23,34].
	1950-1975	(1,1,1,1,1,1)	1.00	Data is from the [28] and [23,34].
Bessemer. RE	1900-1975	(3,3,1,1,1,2)	1.11	The resource efficiency of Bessemer process was similar with open hearth furnace [37]
Bessemer. scrap rate	1900-1975	(1,3,1,1,1,1)	1.07	USGS mineral yearbook for steel scrap allocation
IF. Share	1900-1984	(2,1,1,1,1,2)	1.07	Linear decrease from 25% (based on mass balance) to the rate in 1984
	1985-2015	(2,1,1,1,1,1)	1.02	Production data from Census of World Casting Production
Iron Foundry.RE	1900-2015	(3,4,3,2,1,2)	1.19	Linear growth from 66% [71] in 1900 to 81% in 2008 [2]
Crucible. outflow	1900-1921	(1,2,1,1,1,1)	1.02	Data is from [23,34].
Crucible. RE	1900-1921	(3,3,1,1,1,2)	1.11	Constant data [21] for this period
EAF. outflow	1900-1949	(1,2,1,1,1,1)	1.02	Data is from [23,34].
	1950-2015	(1,1,1,1,1,1)	1.00	Data is from the [28] and [23,34].
EAF. RE	1900-2015	(3,3,1,1,1,3)	1.12	It increased from 77% in 1950 (same with OHF) to 88.9% in 2008 [2]
CC. share	1900-2015	(1,1,1,1,1,1)	1.00	Data from [27,28]
CC. RE	1955-2015	(1,3,1,1,1,1)	1.05	Data from [40]
IC. RE	1900-2015	(1,3,1,1,1,2)	1.07	Follow the same trend with CC. RE but its amount is 13% less [39].
IC-allocated share: Cast steel. share, ISeM. share, IRbM. share, IPtM. share, and IStM. share	1900-2015	(1,3,1,1,1,2)	1.07	Share is based on [2]
CC-allocated share: CSeM. share, CRbM. share, CPtM. share, and CStM. share	1900-2015	(1,3,1,1,1,2)	1.07	Share is based on [2]
SeM. RE	1900-2015	(1,3,1,1,1,2)	1.09	Data from [2]
RbM. RE	1900-2015	(1,3,1,1,1,2)	1.09	Data from [2]
PtM. RE	1900-2015	(1,3,1,1,1,2)	1.09	Data from [2]
StM. RE	1900-2015	(1,3,1,1,1,2)	1.09	Data from [2]
RBM. share	1900-2015	(1,2,1,1,1,1)	1.02	Based on world steel yearbook data
SM. share	1900-2015	(1,2,1,1,1,1)	1.02	Based on world steel yearbook data
CR.RE	1900-2015	(1,3,1,1,1,2)	1.09	Data from [2]

Table S2.8 Uncertainty related to GHG intensity of each steel production technology

Year	Steel production process	Pedigree matrix data quality indicator					Data quality indicators	GSD²
		Reliability	Completeness	Temporal correlation	Geographical correlation	Further technological correlation		
1900-1949	Mining	All data quality indicators set to 5 due to a general lack of certainty about each of the processes before 1950					5,5,5,5,5	3.29
	Sintering						5,5,5,5,5	3.29
	Pelletizing						5,5,5,5,5	3.29
	DRI						5,5,5,5,5	3.29
	BF						5,5,5,5,5	3.29
	Iron Foundry						5,5,5,5,5	3.29
	SM1/Puddling						5,5,5,5,5	3.29
	SM2/OHF						5,5,5,5,5	3.29
	SM3/B-T,C						5,5,5,5,5	3.29
	SM4/BOF						5,5,5,5,5	3.29
	SM5/Crucible						5,5,5,5,5	3.29
	SM6/EAF						5,5,5,5,5	3.29
	Continuous casting						5,5,5,5,5	3.29
	Ingot casting						5,5,5,5,5	3.29
	Section mill						5,5,5,5,5	3.29
	Rod/bar mill						5,5,5,5,5	3.29
	Plate mill						5,5,5,5,5	3.29
	Strip mill						5,5,5,5,5	3.29
	Cold rolling						5,5,5,5,5	3.29
1950-1969	Mining	3; Non-verified data partly based on qualified estimates	5; Representativeness unknown or data from a small number of sites and from shorter periods (Ciroth et al., 2016)	5; estimates refer to data more than 15 years old (Ciroth et al., 2016)	2; global average estimates are used	2; Data from processes and materials under study (i.e. identical technology) but from different enterprises	3,5,5,2,2	1.77
	Sintering	3; Non-verified data partly based on qualified estimates	5; Representativeness unknown or data from a small number of sites and from shorter periods (Ciroth et al., 2016)	5; estimates refer to data more than 15 years old (Ciroth et al., 2016)	2; global average estimates are used	2; Data from processes and materials under study (i.e. identical technology) but from different enterprises	3,5,5,2,2	1.77
	Pelletizing	3; Non-verified data partly based on qualified estimates	5; Representativeness unknown or data from a small number of sites and from shorter periods (Ciroth et al., 2016)	5; estimates refer to data more than 15 years old (Ciroth et al., 2016)	2; global average estimates are used	2; Data from processes and materials under study (i.e. identical technology) but from different enterprises	3,5,5,2,2	1.77
	DRI	3; Non-verified data partly based on qualified estimates	5; Representativeness unknown or data from a small number of sites and from shorter periods (Ciroth et al., 2016)	5; estimates refer to data more than 15 years old (Ciroth et al., 2016)	2; global average estimates are used	3; Data from processes and materials under study from different technology	3,5,5,2,3	2.10

Year	Steel production process	Pedigree matrix data quality indicator					Data quality indicators	GSD²
		Reliability	Completeness	Temporal correlation	Geographical correlation	Further technological correlation		
	BF	3; Non-verified data partly based on qualified estimates	5; Representativeness unknown or data from a small number of sites and from shorter periods (Ciroth et al., 2016)	5; estimates refer to data more than 15 years old (Ciroth et al., 2016)	2; global average estimates are used	2; Data from processes and materials under study (i.e. identical technology) but from different enterprises	3,5,5,2,2	1.77
	Iron Foundry	3; Non-verified data partly based on qualified estimates	5; Representativeness unknown or data from a small number of sites and from shorter periods (Ciroth et al., 2016)	5; estimates refer to data more than 15 years old (Ciroth et al., 2016)	2; global average estimates are used	4; Data on related processes or materials	3,5,5,2,4	2.49
	SM1/Puddling	3; Non-verified data partly based on qualified estimates	5; Representativeness unknown or data from a small number of sites and from shorter periods (Ciroth et al., 2016)	5; estimates refer to data more than 15 years old (Ciroth et al., 2016)	2; global average estimates are used	4; Data on related processes or materials	3,5,5,2,4	2.49
	SM2/OHF	3; Non-verified data partly based on qualified estimates	5; Representativeness unknown or data from a small number of sites and from shorter periods (Ciroth et al., 2016)	5; estimates refer to data more than 15 years old (Ciroth et al., 2016)	2; global average estimates are used	4;Data on related processes or materials	3,5,5,2,4	2.49
	SM3/B-T,C	3; Non-verified data partly based on qualified estimates	5; Representativeness unknown or data from a small number of sites and from shorter periods (Ciroth et al., 2016)	5; estimates refer to data more than 15 years old (Ciroth et al., 2016)	2; global average estimates are used	4; Data on related processes or materials	3,5,5,2,4	2.49
	SM4/BOF	3; Non-verified data partly based on qualified estimates	5; Representativeness unknown or data from a small number of sites and from shorter periods (Ciroth et al., 2016)	5; estimates refer to data more than 15 years old (Ciroth et al., 2016)	2; global average estimates are used	2; Data from processes and materials under study (i.e. identical technology) but from different enterprises	3,5,5,2,2	1.77
	SM5/Crucible	3; Non-verified data partly based on qualified estimates	5; Representativeness unknown or data from a small number of sites and from shorter periods (Ciroth et al., 2016)	5; estimates refer to data more than 15 years old (Ciroth et al., 2016)	2; global average estimates are used	4; Data on related processes or materials	3,5,5,2,4	2.49
	SM6/EAF	3; Non-verified data partly based on qualified estimates	5; Representativeness unknown or data from a small number of sites and from shorter periods (Ciroth et al., 2016)	5; estimates refer to data more than 15 years old (Ciroth et al., 2016)	2; global average estimates are used	2; Data from processes and materials under study (i.e. identical technology) but from different enterprises	3,5,5,2,2	1.77
	Continuous casting	3; Non-verified data partly based on qualified estimates	5; Representativeness unknown or data from a small number of sites and from shorter periods (Ciroth et al., 2016)	5; estimates refer to data more than 15 years old (Ciroth et al., 2016)	2; global average estimates are used	2; Data from processes and materials under study (i.e. identical technology) but from different enterprises	3,5,5,2,2	1.77
	Ingot casting	3; Non-verified data partly based on qualified estimates	5; Representativeness unknown or data from a small number of sites and from shorter periods (Ciroth et al., 2016)	5; estimates refer to data more than 15 years old (Ciroth et al., 2016)	2; global average estimates are used	2; Data from processes and materials under study (i.e. identical technology) but from different enterprises	3,5,5,2,2	1.77

Year	Steel production process	Pedigree matrix data quality indicator					Data quality indicators	GSD[2]
		Reliability	Completeness	Temporal correlation	Geographical correlation	Further technological correlation		
	Section mill	3; Non-verified data partly based on qualified estimates	5; Representativeness unknown or data from a small number of sites and from shorter periods (Ciroth et al., 2016)	5; estimates refer to data more than 15 years old (Ciroth et al., 2016)	2; global average estimates are used	2; Data from processes and materials under study (i.e. identical technology) but from different enterprises	3,5,5,2,2	1.77
	Rod/bar mill	3; Non-verified data partly based on qualified estimates	5; Representativeness unknown or data from a small number of sites and from shorter periods (Ciroth et al., 2016)	5; estimates refer to data more than 15 years old (Ciroth et al., 2016)	2; global average estimates are used	2; Data from processes and materials under study (i.e. identical technology) but from different enterprises	3,5,5,2,2	1.77
	Plate mill	3; Non-verified data partly based on qualified estimates	5; Representativeness unknown or data from a small number of sites and from shorter periods (Ciroth et al., 2016)	5; estimates refer to data more than 15 years old (Ciroth et al., 2016)	2; global average estimates are used	2; Data from processes and materials under study (i.e. identical technology) but from different enterprises	3,5,5,2,2	1.77
	Strip mill	3; Non-verified data partly based on qualified estimates	5; Representativeness unknown or data from a small number of sites and from shorter periods (Ciroth et al., 2016)	5; estimates refer to data more than 15 years old (Ciroth et al., 2016)	2; global average estimates are used	2; Data from processes and materials under study (i.e. identical technology) but from different enterprises	3,5,5,2,2	1.77
	Cold rolling	3; Non-verified data partly based on qualified estimates	5; Representativeness unknown or data from a small number of sites and from shorter periods (Ciroth et al., 2016)	5; estimates refer to data more than 15 years old (Ciroth et al., 2016)	2; global average estimates are used	2; Data from processes and materials under study (i.e. identical technology) but from different enterprises	3,5,5,2,2	1.77
1970-1989	Mining	3; Non-verified data partly based on qualified estimates	5; Representativeness unknown or data from a small number of sites and from shorter periods (Ciroth et al., 2016)	5; estimates refer to data more than 15 years old (Ciroth et al., 2016)	2; global average estimates are used	2; Data from processes and materials under study (i.e. identical technology) but from different enterprises	3,5,5,2,2	1.77
	Sintering	3; Non-verified data partly based on qualified estimates	5; Representativeness unknown or data from a small number of sites and from shorter periods (Ciroth et al., 2016)	5; estimates refer to data more than 15 years old (Ciroth et al., 2016)	2; global average estimates are used	2; Data from processes and materials under study (i.e. identical technology) but from different enterprises	3,5,5,2,2	1.77
	Pelletizing	3; Non-verified data partly based on qualified estimates	5; Representativeness unknown or data from a small number of sites and from shorter periods (Ciroth et al., 2016)	5; estimates refer to data more than 15 years old (Ciroth et al., 2016)	2; global average estimates are used	2; Data from processes and materials under study (i.e. identical technology) but from different enterprises	3,5,5,2,2	1.77
	DRI	3; Non-verified data partly based on qualified estimates	5; Representativeness unknown or data from a small number of sites and from shorter periods (Ciroth et al., 2016)	5; estimates refer to data more than 15 years old (Ciroth et al., 2016)	2; global average estimates are used	3; Data from processes and materials under study from different technology	3,5,5,2,3	2.10
	BF	3; Non-verified data partly based on qualified estimates	5; Representativeness unknown or data from a small number of sites and from shorter periods (Ciroth et al., 2016)	5; estimates refer to data more than 15 years old (Ciroth et al., 2016)	2; global average estimates are used	2; Data from processes and materials under study (i.e. identical technology) but from different enterprises	3,5,5,2,2	1.77

Year	Steel production process	Pedigree matrix data quality indicator					Data quality indicators	GSD²
		Reliability	Completeness	Temporal correlation	Geographical correlation	Further technological correlation		
	Iron Foundry	3; Non-verified data partly based on qualified estimates	5; Representativeness unknown or data from a small number of sites and from shorter periods (Ciroth et al., 2016)	5; estimates refer to data more than 15 years old (Ciroth et al., 2016)	2; global average estimates are used	4; Data on related processes or materials	3,5,5,2,4	2.49
	SM1/Puddling	3; Non-verified data partly based on qualified estimates	5; Representativeness unknown or data from a small number of sites and from shorter periods (Ciroth et al., 2016)	5; estimates refer to data more than 15 years old (Ciroth et al., 2016)	2; global average estimates are used	4; Data on related processes or materials	3,5,5,2,4	2.49
	SM2/OHF	3; Non-verified data partly based on qualified estimates	5; Representativeness unknown or data from a small number of sites and from shorter periods (Ciroth et al., 2016)	5; estimates refer to data more than 15 years old (Ciroth et al., 2016)	2; global average estimates are used	4;Data on related processes or materials	3,5,5,2,4	2.49
	SM3/B-T,C	3; Non-verified data partly based on qualified estimates	5; Representativeness unknown or data from a small number of sites and from shorter periods (Ciroth et al., 2016)	5; estimates refer to data more than 15 years old (Ciroth et al., 2016)	2; global average estimates are used	4; Data on related processes or materials	3,5,5,2,4	2.49
	SM4/BOF	3; Non-verified data partly based on qualified estimates	5; Representativeness unknown or data from a small number of sites and from shorter periods (Ciroth et al., 2016)	5; estimates refer to data more than 15 years old (Ciroth et al., 2016)	2; global average estimates are used	2; Data from processes and materials under study (i.e. identical technology) but from different enterprises	3,5,5,2,2	1.77
	SM5/Crucible	3; Non-verified data partly based on qualified estimates	5; Representativeness unknown or data from a small number of sites and from shorter periods (Ciroth et al., 2016)	5; estimates refer to data more than 15 years old (Ciroth et al., 2016)	2; global average estimates are used	4; Data on related processes or materials	3,5,5,2,4	2.49
	SM6/EAF	3; Non-verified data partly based on qualified estimates	5; Representativeness unknown or data from a small number of sites and from shorter periods (Ciroth et al., 2016)	5; estimates refer to data more than 15 years old (Ciroth et al., 2016)	2; global average estimates are used	2; Data from processes and materials under study (i.e. identical technology) but from different enterprises	3,5,5,2,2	1.77
	Continuous casting	3; Non-verified data partly based on qualified estimates	5; Representativeness unknown or data from a small number of sites and from shorter periods (Ciroth et al., 2016)	5; estimates refer to data more than 15 years old (Ciroth et al., 2016)	2; global average estimates are used	2; Data from processes and materials under study (i.e. identical technology) but from different enterprises	3,5,5,2,2	1.77
	Ingot casting	3; Non-verified data partly based on qualified estimates	5; Representativeness unknown or data from a small number of sites and from shorter periods (Ciroth et al., 2016)	5; estimates refer to data more than 15 years old (Ciroth et al., 2016)	2; global average estimates are used	2; Data from processes and materials under study (i.e. identical technology) but from different enterprises	3,5,5,2,2	1.77
	Section mill	3; Non-verified data partly based on qualified estimates	5; Representativeness unknown or data from a small number of sites and from shorter periods (Ciroth et al., 2016)	5; estimates refer to data more than 15 years old (Ciroth et al., 2016)	2; global average estimates are used	2; Data from processes and materials under study (i.e. identical technology) but from different enterprises	3,5,5,2,2	1.77

Year	Steel production process	Pedigree matrix data quality indicator					Data quality indicators	GSD²
		Reliability	Completeness	Temporal correlation	Geographical correlation	Further technological correlation		
	Rod/bar mill	3; Non-verified data partly based on qualified estimates	5; Representativeness unknown or data from a small number of sites and from shorter periods (Ciroth et al., 2016)	5; estimates refer to data more than 15 years old (Ciroth et al., 2016)	2; global average estimates are used	2; Data from processes and materials under study (i.e. identical technology) but from different enterprises	3,5,5,2,2	1.77
	Plate mill	3; Non-verified data partly based on qualified estimates	5; Representativeness unknown or data from a small number of sites and from shorter periods (Ciroth et al., 2016)	5; estimates refer to data more than 15 years old (Ciroth et al., 2016)	2; global average estimates are used	2; Data from processes and materials under study (i.e. identical technology) but from different enterprises	3,5,5,2,2	1.77
	Strip mill	3; Non-verified data partly based on qualified estimates	5; Representativeness unknown or data from a small number of sites and from shorter periods (Ciroth et al., 2016)	5; estimates refer to data more than 15 years old (Ciroth et al., 2016)	2; global average estimates are used	2; Data from processes and materials under study (i.e. identical technology) but from different enterprises	3,5,5,2,2	1.77
	Cold rolling	3; Non-verified data partly based on qualified estimates	5; Representativeness unknown or data from a small number of sites and from shorter periods (Ciroth et al., 2016)	5; estimates refer to data more than 15 years old (Ciroth et al., 2016)	2; global average estimates are used	2; Data from processes and materials under study (i.e. identical technology) but from different enterprises	3,5,5,2,2	1.77
1990-1999	Mining	3; Non-verified data partly based on qualified estimates	5; Representativeness unknown or data from a small number of sites and from shorter periods (Ciroth et al., 2016)	5; estimates refer to data more than 15 years old (Ciroth et al., 2016)	2; global average estimates are used	2; Data from processes and materials under study (i.e. identical technology) but from different enterprises	3,5,5,2,2	1.77
	Sintering	3; Non-verified data partly based on qualified estimates	5; Representativeness unknown or data from a small number of sites and from shorter periods (Ciroth et al., 2016)	5; estimates refer to data more than 15 years old (Ciroth et al., 2016)	2; global average estimates are used	2; Data from processes and materials under study (i.e. identical technology) but from different enterprises	3,5,5,2,2	1.77
	Pelletizing	3; Non-verified data partly based on qualified estimates	5; Representativeness unknown or data from a small number of sites and from shorter periods (Ciroth et al., 2016)	5; estimates refer to data more than 15 years old (Ciroth et al., 2016)	2; global average estimates are used	2; Data from processes and materials under study (i.e. identical technology) but from different enterprises	3,5,5,2,2	1.77
	DRI	3; Non-verified data partly based on qualified estimates	5; Representativeness unknown or data from a small number of sites and from shorter periods (Ciroth et al., 2016)	5; estimates refer to data more than 15 years old (Ciroth et al., 2016)	2; global average estimates are used	3; Data from processes and materials under study from different technology	3,5,5,2,3	2.10
	BF	3; Non-verified data partly based on qualified estimates	5; Representativeness unknown or data from a small number of sites and from shorter periods (Ciroth et al., 2016)	5; estimates refer to data more than 15 years old (Ciroth et al., 2016)	2; global average estimates are used	2; Data from processes and materials under study (i.e. identical technology) but from different enterprises	3,5,5,2,2	1.77
	Iron Foundry	3; Non-verified data partly based on qualified estimates	5; Representativeness unknown or data from a small number of sites and from shorter periods (Ciroth et al., 2016)	5; estimates refer to data more than 15 years old (Ciroth et al., 2016)	2; global average estimates are used	4; Data on related processes or materials	3,5,5,2,4	2.49

Year	Steel production process	Pedigree matrix data quality indicator					Data quality indicators	GSD[2]
		Reliability	Completeness	Temporal correlation	Geographical correlation	Further technological correlation		
	SM1/Puddling	3; Non-verified data partly based on qualified estimates	5; Representativeness unknown or data from a small number of sites and from shorter periods (Ciroth et al., 2016)	5; estimates refer to data more than 15 years old (Ciroth et al., 2016)	2; global average estimates are used	4; Data on related processes or materials	3,5,5,2,4	2.49
	SM2/OHF	3; Non-verified data partly based on qualified estimates	5; Representativeness unknown or data from a small number of sites and from shorter periods (Ciroth et al., 2016)	5; estimates refer to data more than 15 years old (Ciroth et al., 2016)	2; global average estimates are used	4;Data on related processes or materials	3,5,5,2,4	2.49
	SM3/B-T,C	3; Non-verified data partly based on qualified estimates	5; Representativeness unknown or data from a small number of sites and from shorter periods (Ciroth et al., 2016)	5; estimates refer to data more than 15 years old (Ciroth et al., 2016)	2; global average estimates are used	4; Data on related processes or materials	3,5,5,2,4	2.49
	SM4/BOF	3; Non-verified data partly based on qualified estimates	5; Representativeness unknown or data from a small number of sites and from shorter periods (Ciroth et al., 2016)	5; estimates refer to data more than 15 years old (Ciroth et al., 2016)	2; global average estimates are used	2; Data from processes and materials under study (i.e. identical technology) but from different enterprises	3,5,5,2,2	1.77
	SM5/Crucible	3; Non-verified data partly based on qualified estimates	5; Representativeness unknown or data from a small number of sites and from shorter periods (Ciroth et al., 2016)	5; estimates refer to data more than 15 years old (Ciroth et al., 2016)	2; global average estimates are used	4; Data on related processes or materials	3,5,5,2,4	2.49
	SM6/EAF	3; Non-verified data partly based on qualified estimates	5; Representativeness unknown or data from a small number of sites and from shorter periods (Ciroth et al., 2016)	5; estimates refer to data more than 15 years old (Ciroth et al., 2016)	2; global average estimates are used	2; Data from processes and materials under study (i.e. identical technology) but from different enterprises	3,5,5,2,2	1.77
	Continuous casting	3; Non-verified data partly based on qualified estimates	5; Representativeness unknown or data from a small number of sites and from shorter periods (Ciroth et al., 2016)	5; estimates refer to data more than 15 years old (Ciroth et al., 2016)	2; global average estimates are used	2; Data from processes and materials under study (i.e. identical technology) but from different enterprises	3,5,5,2,2	1.77
	Ingot casting	3; Non-verified data partly based on qualified estimates	5; Representativeness unknown or data from a small number of sites and from shorter periods (Ciroth et al., 2016)	5; estimates refer to data more than 15 years old (Ciroth et al., 2016)	2; global average estimates are used	2; Data from processes and materials under study (i.e. identical technology) but from different enterprises	3,5,5,2,2	1.77
	Section mill	3; Non-verified data partly based on qualified estimates	5; Representativeness unknown or data from a small number of sites and from shorter periods (Ciroth et al., 2016)	5; estimates refer to data more than 15 years old (Ciroth et al., 2016)	2; global average estimates are used	2; Data from processes and materials under study (i.e. identical technology) but from different enterprises	3,5,5,2,2	1.77
	Rod/bar mill	3; Non-verified data partly based on qualified estimates	5; Representativeness unknown or data from a small number of sites and from shorter periods (Ciroth et al., 2016)	5; estimates refer to data more than 15 years old (Ciroth et al., 2016)	2; global average estimates are used	2; Data from processes and materials under study (i.e. identical technology) but from different enterprises	3,5,5,2,2	1.77

Year	Steel production process	Pedigree matrix data quality indicator					Data quality indicators	GSD[2]
		Reliability	Completeness	Temporal correlation	Geographical correlation	Further technological correlation		
	Plate mill	3; Non-verified data partly based on qualified estimates	5; Representativeness unknown or data from a small number of sites and from shorter periods (Ciroth et al., 2016)	5; estimates refer to data more than 15 years old (Ciroth et al., 2016)	2; global average estimates are used	2; Data from processes and materials under study (i.e. identical technology) but from different enterprises	3,5,5,2,2	1.77
	Strip mill	3; Non-verified data partly based on qualified estimates	5; Representativeness unknown or data from a small number of sites and from shorter periods (Ciroth et al., 2016)	5; estimates refer to data more than 15 years old (Ciroth et al., 2016)	2; global average estimates are used	2; Data from processes and materials under study (i.e. identical technology) but from different enterprises	3,5,5,2,2	1.77
	Cold rolling	3; Non-verified data partly based on qualified estimates	5; Representativeness unknown or data from a small number of sites and from shorter periods (Ciroth et al., 2016)	5; estimates refer to data more than 15 years old (Ciroth et al., 2016)	2; global average estimates are used	2; Data from processes and materials under study (i.e. identical technology) but from different enterprises	3,5,5,2,2	1.77
2000-2009	Mining	2; Verified data partly based on assumptions or non-verified data based on measurements	5; Representativeness unknown or data from a small number of sites and from shorter periods (Ciroth et al., 2016)	4; Less than 15 years of difference to the time period of the dataset (Ciroth et al., 2016)	2; global average estimates are used	2; Data from processes and materials under study (i.e. identical technology) but from different enterprises	2,5,4,2,2	1.65
	Sintering	2; Verified data partly based on assumptions or non-verified data based on measurements	5; Representativeness unknown or data from a small number of sites and from shorter periods (Ciroth et al., 2016)	4; Less than 15 years of difference to the time period of the dataset (Ciroth et al., 2016)	2; global average estimates are used	2; Data from processes and materials under study (i.e. identical technology) but from different enterprises	2,5,4,2,2	1.65
	Pelletizing	2; Verified data partly based on assumptions or non-verified data based on measurements	5; Representativeness unknown or data from a small number of sites and from shorter periods (Ciroth et al., 2016)	4; Less than 15 years of difference to the time period of the dataset (Ciroth et al., 2016)	2; global average estimates are used	2; Data from processes and materials under study (i.e. identical technology) but from different enterprises	2,5,4,2,2	1.65
	DRI	2; Verified data partly based on assumptions or non-verified data based on measurements	5; Representativeness unknown or data from a small number of sites and from shorter periods (Ciroth et al., 2016)	4; Less than 15 years of difference to the time period of the dataset (Ciroth et al., 2016)	2; global average estimates are used	3; Data from processes and materials under study from different technology	2,5,4,2,3	1.99
	BF	2; Verified data partly based on assumptions or non-verified data based on measurements	5; Representativeness unknown or data from a small number of sites and from shorter periods (Ciroth et al., 2016)	4; Less than 15 years of difference to the time period of the dataset (Ciroth et al., 2016)	2; global average estimates are used	2; Data from processes and materials under study (i.e. identical technology) but from different enterprises	2,5,4,2,2	1.65
	Iron Foundry	2; Verified data partly based on assumptions or non-verified data based on measurements	5; Representativeness unknown or data from a small number of sites and from shorter periods (Ciroth et al., 2016)	4; Less than 15 years of difference to the time period of the dataset (Ciroth et al., 2016)	2; global average estimates are used	4; Data on related processes or materials	2,5,4,2,4	2.39

Year	Steel production process	Pedigree matrix data quality indicator					Data quality indicators	GSD[2]
		Reliability	Completeness	Temporal correlation	Geographical correlation	Further technological correlation		
	SM1/Puddling	2; Verified data partly based on assumptions or non-verified data based on measurements	5; Representativeness unknown or data from a small number of sites and from shorter periods (Ciroth et al., 2016)	4; Less than 15 years of difference to the time period of the dataset (Ciroth et al., 2016)	2; global average estimates are used	4; Data on related processes or materials	2,5,4,2,4	2.39
	SM2/OHF	2; Verified data partly based on assumptions or non-verified data based on measurements	5; Representativeness unknown or data from a small number of sites and from shorter periods (Ciroth et al., 2016)	4; Less than 15 years of difference to the time period of the dataset (Ciroth et al., 2016)	2; global average estimates are used	4;Data on related processes or materials	2,5,4,2,4	2.39
	SM3/B-T,C	2; Verified data partly based on assumptions or non-verified data based on measurements	5; Representativeness unknown or data from a small number of sites and from shorter periods (Ciroth et al., 2016)	4; Less than 15 years of difference to the time period of the dataset (Ciroth et al., 2016)	2; global average estimates are used	4; Data on related processes or materials	2,5,4,2,4	2.39
	SM4/BOF	2; Verified data partly based on assumptions or non-verified data based on measurements	5; Representativeness unknown or data from a small number of sites and from shorter periods (Ciroth et al., 2016)	4; Less than 15 years of difference to the time period of the dataset (Ciroth et al., 2016)	2; global average estimates are used	2; Data from processes and materials under study (i.e. identical technology) but from different enterprises	2,5,4,2,2	1.65
	SM5/Crucible	2; Verified data partly based on assumptions or non-verified data based on measurements	5; Representativeness unknown or data from a small number of sites and from shorter periods (Ciroth et al., 2016)	4; Less than 15 years of difference to the time period of the dataset (Ciroth et al., 2016)	2; global average estimates are used	4; Data on related processes or materials	2,5,4,2,4	2.39
	SM6/EAF	2; Verified data partly based on assumptions or non-verified data based on measurements	5; Representativeness unknown or data from a small number of sites and from shorter periods (Ciroth et al., 2016)	4; Less than 15 years of difference to the time period of the dataset (Ciroth et al., 2016)	2; global average estimates are used	2; Data from processes and materials under study (i.e. identical technology) but from different enterprises	2,5,4,2,2	1.65
	Continuous casting	2; Verified data partly based on assumptions or non-verified data based on measurements	5; Representativeness unknown or data from a small number of sites and from shorter periods (Ciroth et al., 2016)	4; Less than 15 years of difference to the time period of the dataset (Ciroth et al., 2016)	2; global average estimates are used	2; Data from processes and materials under study (i.e. identical technology) but from different enterprises	2,5,4,2,2	1.65
	Ingot casting	2; Verified data partly based on assumptions or non-verified data based on measurements	5; Representativeness unknown or data from a small number of sites and from shorter periods (Ciroth et al., 2016)	4; Less than 15 years of difference to the time period of the dataset (Ciroth et al., 2016)	2; global average estimates are used	2; Data from processes and materials under study (i.e. identical technology) but from different enterprises	2,5,4,2,2	1.65

Year	Steel production process	Pedigree matrix data quality indicator					Data quality indicators	GSD²
		Reliability	Completeness	Temporal correlation	Geographical correlation	Further technological correlation		
	Section mill	2; Verified data partly based on assumptions or non-verified data based on measurements	5; Representativeness unknown or data from a small number of sites and from shorter periods (Ciroth et al., 2016)	4; Less than 15 years of difference to the time period of the dataset (Ciroth et al., 2016)	2; global average estimates are used	2; Data from processes and materials under study (i.e. identical technology) but from different enterprises	2,5,4,2,2	1.65
	Rod/bar mill	2; Verified data partly based on assumptions or non-verified data based on measurements	5; Representativeness unknown or data from a small number of sites and from shorter periods (Ciroth et al., 2016)	4; Less than 15 years of difference to the time period of the dataset (Ciroth et al., 2016)	2; global average estimates are used	2; Data from processes and materials under study (i.e. identical technology) but from different enterprises	2,5,4,2,2	1.65
	Plate mill	2; Verified data partly based on assumptions or non-verified data based on measurements	5; Representativeness unknown or data from a small number of sites and from shorter periods (Ciroth et al., 2016)	4; Less than 15 years of difference to the time period of the dataset (Ciroth et al., 2016)	2; global average estimates are used	2; Data from processes and materials under study (i.e. identical technology) but from different enterprises	2,5,4,2,2	1.65
	Strip mill	2; Verified data partly based on assumptions or non-verified data based on measurements	5; Representativeness unknown or data from a small number of sites and from shorter periods (Ciroth et al., 2016)	4; Less than 15 years of difference to the time period of the dataset (Ciroth et al., 2016)	2; global average estimates are used	2; Data from processes and materials under study (i.e. identical technology) but from different enterprises	2,5,4,2,2	1.65
	Cold rolling	2; Verified data partly based on assumptions or non-verified data based on measurements	5; Representativeness unknown or data from a small number of sites and from shorter periods (Ciroth et al., 2016)	4; Less than 15 years of difference to the time period of the dataset (Ciroth et al., 2016)	2; global average estimates are used	2; Data from processes and materials under study (i.e. identical technology) but from different enterprises	2,5,4,2,2	1.65
2010-2015	Mining	2; Verified data partly based on assumptions or non-verified data based on measurements	5; Representativeness unknown or data from a small number of sites and from shorter periods (Ciroth et al., 2016)	2; Less than 6 years of difference of the time period of the dataset (Ciroth et al., 2016)	2; global average estimates are used	2; Data from processes and materials under study (i.e. identical technology) but from different enterprises	2,5,2,2,2	1.60
	Sintering	2; Verified data partly based on assumptions or non-verified data based on measurements	5; Representativeness unknown or data from a small number of sites and from shorter periods (Ciroth et al., 2016)	2; Less than 6 years of difference of the time period of the dataset (Ciroth et al., 2016)	2; global average estimates are used	2; Data from processes and materials under study (i.e. identical technology) but from different enterprises	2,5,2,2,2	1.60
	Pelletizing	2; Verified data partly based on assumptions or non-verified data based on measurements	5; Representativeness unknown or data from a small number of sites and from shorter periods (Ciroth et al., 2016)	2; Less than 6 years of difference of the time period of the dataset (Ciroth et al., 2016)	2; global average estimates are used	2; Data from processes and materials under study (i.e. identical technology) but from different enterprises	2,5,2,2,2	1.60

Year	Steel production process	Pedigree matrix data quality indicator					Data quality indicators	GSD²
		Reliability	Completeness	Temporal correlation	Geographical correlation	Further technological correlation		
	DRI	2; Verified data partly based on assumptions or non-verified data based on measurements	5; Representativeness unknown or data from a small number of sites and from shorter periods (Ciroth et al., 2016)	2; Less than 6 years of difference of the time period of the dataset (Ciroth et al., 2016)	2; global average estimates are used	3; Data from processes and materials under study from different technology	2,5,2,2,3	1.95
	BF	2; Verified data partly based on assumptions or non-verified data based on measurements	5; Representativeness unknown or data from a small number of sites and from shorter periods (Ciroth et al., 2016)	2; Less than 6 years of difference of the time period of the dataset (Ciroth et al., 2016)	2; global average estimates are used	2; Data from processes and materials under study (i.e. identical technology) but from different enterprises	2,5,2,2,2	1.60
	Iron Foundry	2; Verified data partly based on assumptions or non-verified data based on measurements	5; Representativeness unknown or data from a small number of sites and from shorter periods (Ciroth et al., 2016)	2; Less than 6 years of difference of the time period of the dataset (Ciroth et al., 2016)	2; global average estimates are used	4; Data on related processes or materials	2,5,2,2,4	2.35
	SM1/Puddling	2; Verified data partly based on assumptions or non-verified data based on measurements	5; Representativeness unknown or data from a small number of sites and from shorter periods (Ciroth et al., 2016)	2; Less than 6 years of difference of the time period of the dataset (Ciroth et al., 2016)	2; global average estimates are used	4; Data on related processes or materials	2,5,2,2,4	2.35
	SM2/OHF	2; Verified data partly based on assumptions or non-verified data based on measurements	5; Representativeness unknown or data from a small number of sites and from shorter periods (Ciroth et al., 2016)	2; Less than 6 years of difference of the time period of the dataset (Ciroth et al., 2016)	2; global average estimates are used	4;Data on related processes or materials	2,5,2,2,4	2.35
	SM3/B-T,C	2; Verified data partly based on assumptions or non-verified data based on measurements	5; Representativeness unknown or data from a small number of sites and from shorter periods (Ciroth et al., 2016)	2; Less than 6 years of difference of the time period of the dataset (Ciroth et al., 2016)	2; global average estimates are used	4; Data on related processes or materials	2,5,2,2,4	2.35
	SM4/BOF	2; Verified data partly based on assumptions or non-verified data based on measurements	5; Representativeness unknown or data from a small number of sites and from shorter periods (Ciroth et al., 2016)	2; Less than 6 years of difference of the time period of the dataset (Ciroth et al., 2016)	2; global average estimates are used	2; Data from processes and materials under study (i.e. identical technology) but from different enterprises	2,5,2,2,2	1.60
	SM5/Crucible	2; Verified data partly based on assumptions or non-verified data based on measurements	5; Representativeness unknown or data from a small number of sites and from shorter periods (Ciroth et al., 2016)	2; Less than 6 years of difference of the time period of the dataset (Ciroth et al., 2016)	2; global average estimates are used	4; Data on related processes or materials	2,5,2,2,4	2.35

Year	Steel production process	Pedigree matrix data quality indicator					Data quality indicators	GSD²
		Reliability	Completeness	Temporal correlation	Geographical correlation	Further technological correlation		
	SM6/EAF	2; Verified data partly based on assumptions or non-verified data based on measurements	5; Representativeness unknown or data from a small number of sites and from shorter periods (Ciroth et al., 2016)	2; Less than 6 years of difference of the time period of the dataset (Ciroth et al., 2016)	2; global average estimates are used	2; Data from processes and materials under study (i.e. identical technology) but from different enterprises	2,5,2,2,2	1.60
	Continuous casting	2; Verified data partly based on assumptions or non-verified data based on measurements	5; Representativeness unknown or data from a small number of sites and from shorter periods (Ciroth et al., 2016)	2; Less than 6 years of difference of the time period of the dataset (Ciroth et al., 2016)	2; global average estimates are used	2; Data from processes and materials under study (i.e. identical technology) but from different enterprises	2,5,2,2,2	1.60
	Ingot casting	2; Verified data partly based on assumptions or non-verified data based on measurements	5; Representativeness unknown or data from a small number of sites and from shorter periods (Ciroth et al., 2016)	2; Less than 6 years of difference of the time period of the dataset (Ciroth et al., 2016)	2; global average estimates are used	2; Data from processes and materials under study (i.e. identical technology) but from different enterprises	2,5,2,2,2	1.60
	Section mill	2; Verified data partly based on assumptions or non-verified data based on measurements	5; Representativeness unknown or data from a small number of sites and from shorter periods (Ciroth et al., 2016)	2; Less than 6 years of difference of the time period of the dataset (Ciroth et al., 2016)	2; global average estimates are used	2; Data from processes and materials under study (i.e. identical technology) but from different enterprises	2,5,2,2,2	1.60
	Rod/bar mill	2; Verified data partly based on assumptions or non-verified data based on measurements	5; Representativeness unknown or data from a small number of sites and from shorter periods (Ciroth et al., 2016)	2; Less than 6 years of difference of the time period of the dataset (Ciroth et al., 2016)	2; global average estimates are used	2; Data from processes and materials under study (i.e. identical technology) but from different enterprises	2,5,2,2,2	1.60
	Plate mill	2; Verified data partly based on assumptions or non-verified data based on measurements	5; Representativeness unknown or data from a small number of sites and from shorter periods (Ciroth et al., 2016)	2; Less than 6 years of difference of the time period of the dataset (Ciroth et al., 2016)	2; global average estimates are used	2; Data from processes and materials under study (i.e. identical technology) but from different enterprises	2,5,2,2,2	1.60
	Strip mill	2; Verified data partly based on assumptions or non-verified data based on measurements	5; Representativeness unknown or data from a small number of sites and from shorter periods (Ciroth et al., 2016)	2; Less than 6 years of difference of the time period of the dataset (Ciroth et al., 2016)	2; global average estimates are used	2; Data from processes and materials under study (i.e. identical technology) but from different enterprises	2,5,2,2,2	1.60
	Cold rolling	2; Verified data partly based on assumptions or non-verified data based on measurements	5; Representativeness unknown or data from a small number of sites and from shorter periods (Ciroth et al., 2016)	2; Less than 6 years of difference of the time period of the dataset (Ciroth et al., 2016)	2; global average estimates are used	2; Data from processes and materials under study (i.e. identical technology) but from different enterprises	2,5,2,2,2	1.60

S3. Results and their uncertainties

S3.1 Material Flow Results

In this section, the related activity data sets for emission calculation are presented in the first stage. Afterwards, other relevant data series (i.e. in-use stock trend, end-of-life scrap trend, and EoL (End-of-life) recycling rate, etc.) for our analysis are presented.

(1) Production activity data sets

The impact analysis requires the outflow data of 19 studied processes, which are obtained either from official statistical data sources or from our material flow model.

As shown in Fig.S3.1, there are 9 parameters obtained directly from official statistical data sources, including mining production from [13,30], DRI production from [19], EAF, OHF, and BOF production from [23,28,34], and Puddling, Bessemer, and Crucible from [23,34]. Given those that data are obtained from the official statistical data sources, the uncertainties of those trends are very low.

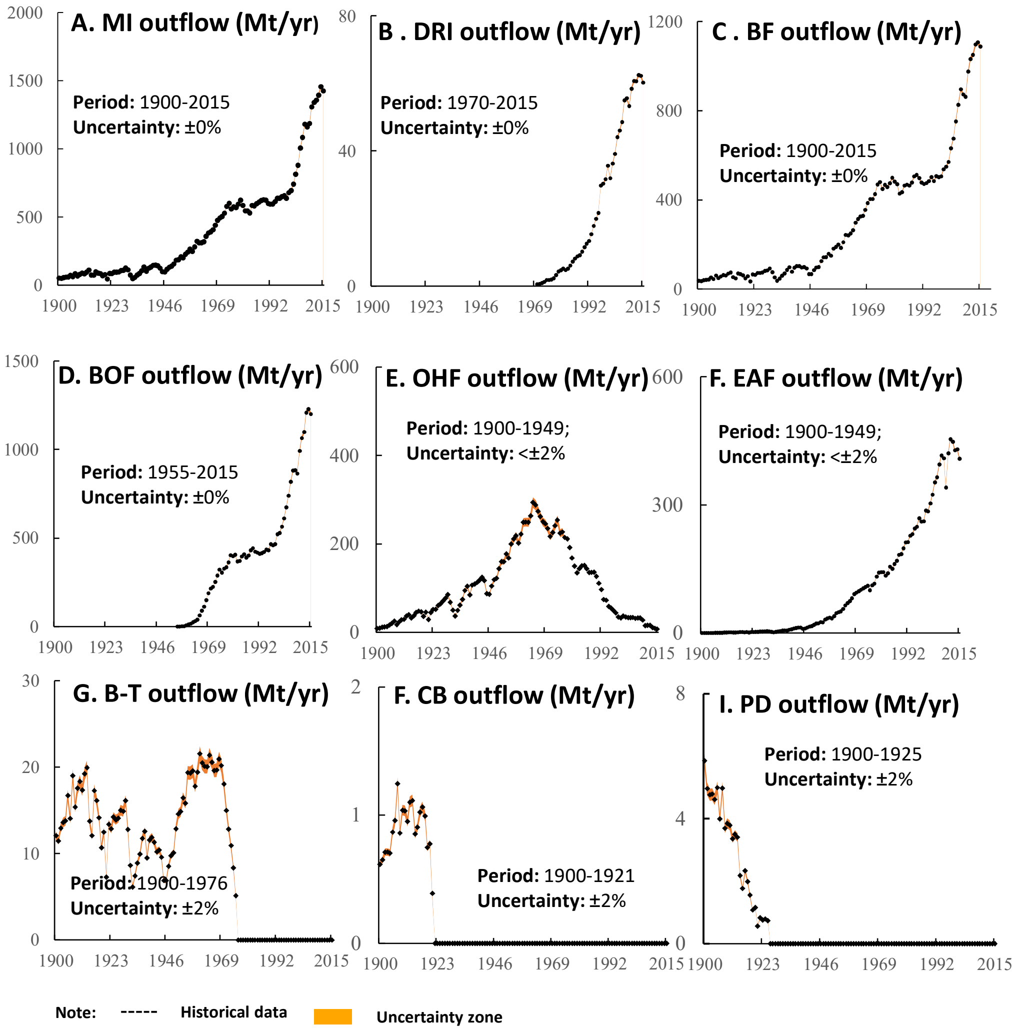

Fig. S3.1 Statistics-based outflow of studied processes. Data are presented as the deterministic results and the shaded areas indicate the 95% confidence interval of the estimates.

Meanwhile, for the rest 10 studied processes, their outflows are obtained based on the dynamic material flow analysis. Those method-based results and their uncertainties are shown in Fig. S3.2. Compared to those statistics-based results in Fig. S3.1, the uncertainty levels of those results are relatively higher. However, their impact on the total results is quite low, given their relatively small magnitude and low impact intensity. Consequently, the results from material flow analysis are acceptable for impact analysis, and a Monte-Carlo analysis will be conducted for the uncertainty analysis.

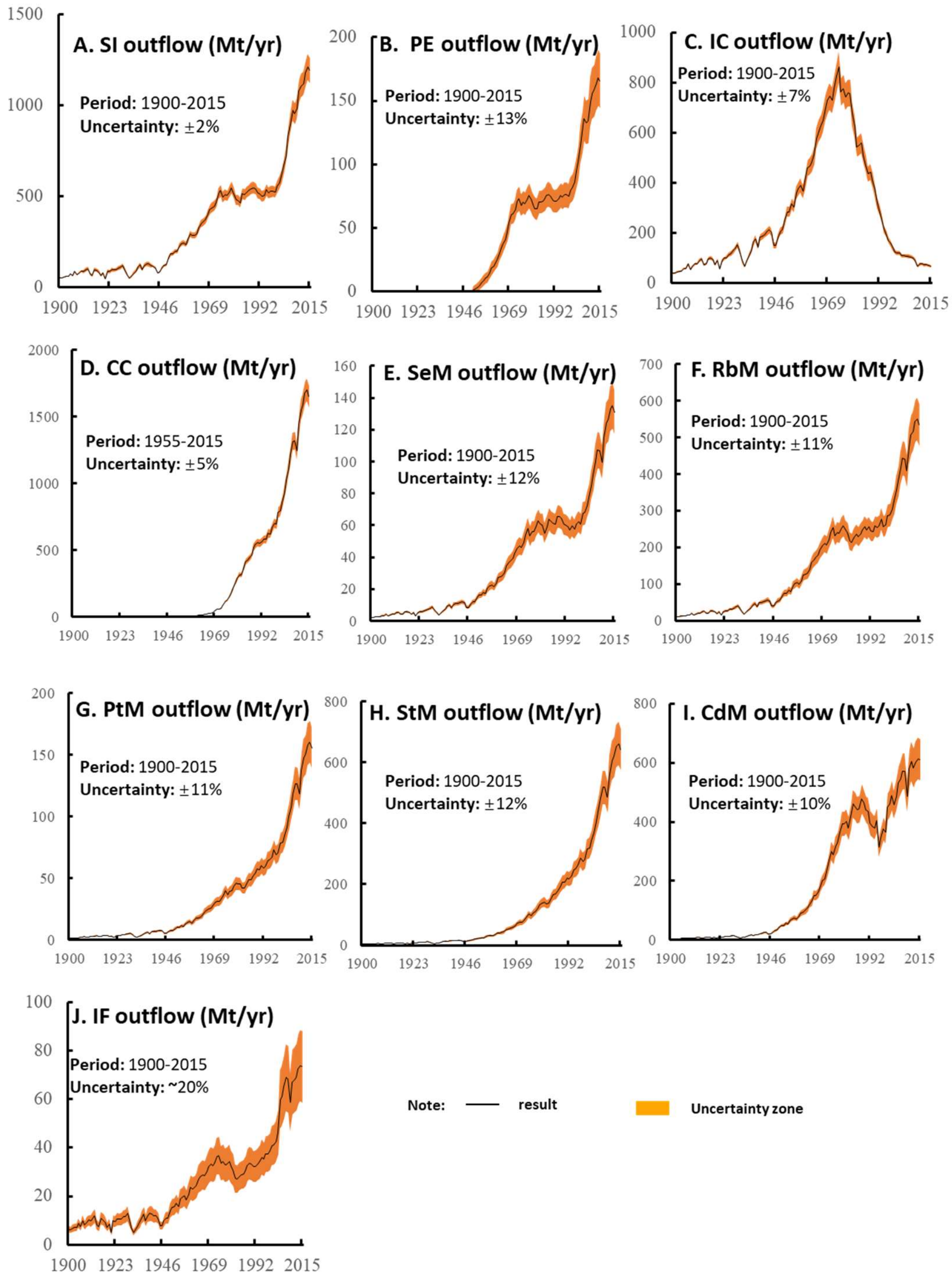

Fig. S3.2 The estimated outflow data of studied processes. Data are presented as the deterministic results and the shaded areas indicate the 95% confidence interval of the estimates.

(2) **Data sets for in-use stock, scrap, and recycling rates**

This study estimated the annual amount of steel flow and stocks in the fabrication, in-use and end-of-life stage. The major results regarding in-use stock, scrap amount and recycling rate is shown in Fig. S3.3.

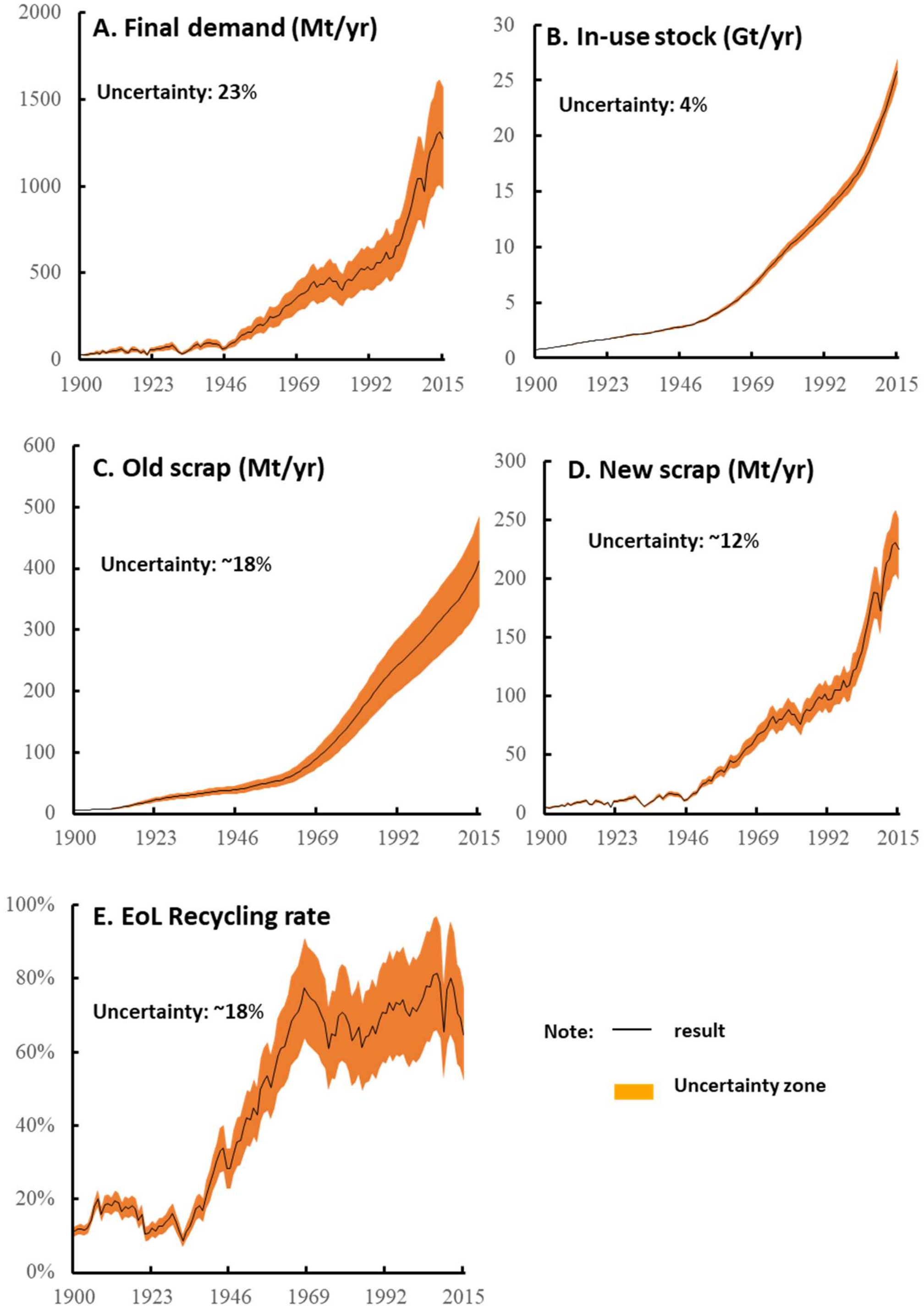

Fig. S3.3 Results for stocks, recycling flows and rates. Data are presented as the deterministic results and the shaded areas indicate the 95% confidence interval of the estimates.

S3.2 GHG Emission Results

The historical GHG emisison associated with global steel production is presented in Fig.S3.4, while the detailed tracjectory for each technology is shown in Fig.3.5 and Fig. S3.6.

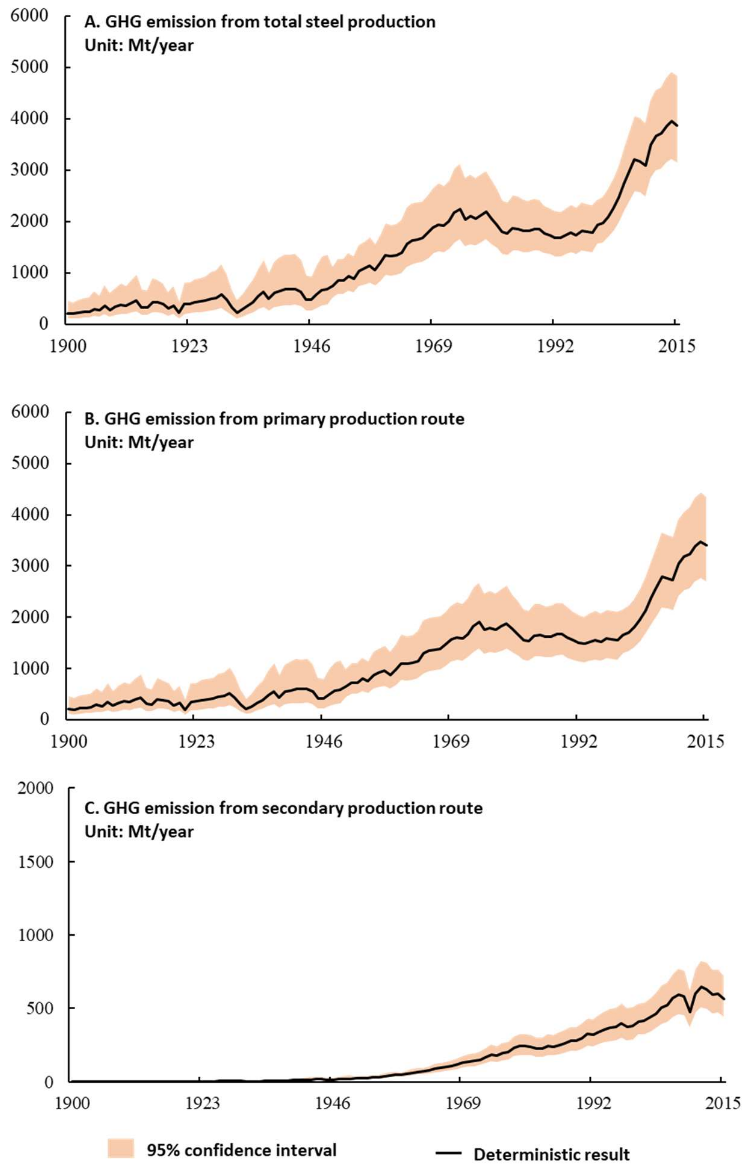

Fig. S3.4 GHG emission associated with global steel production from 1900 to 2015. Data are presented as the deterministic results and the shaded areas indicate the 95% confidence interval of the estimates.

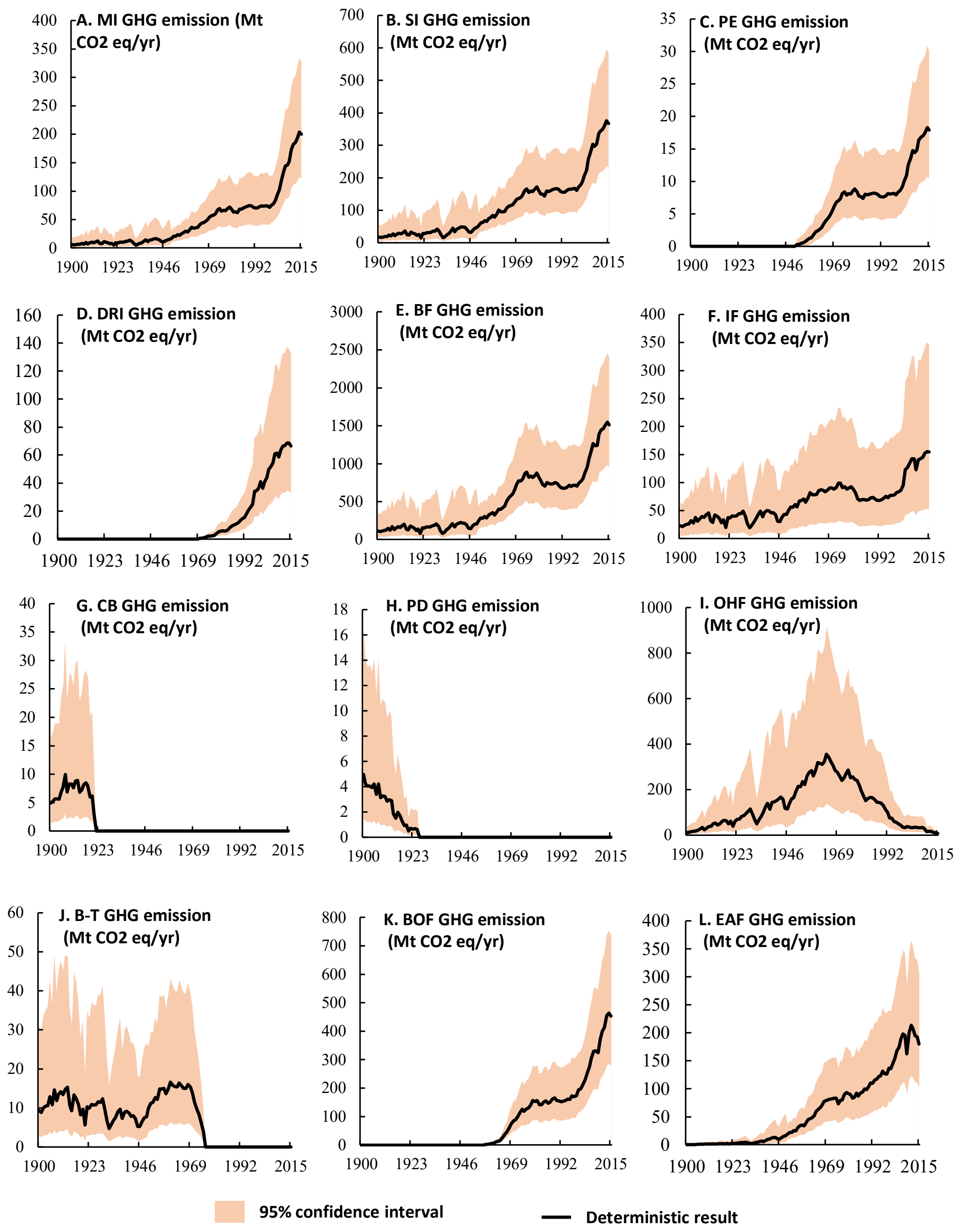

Fig. S3.5 GHG emission for ironmaking and steelmaking processes. Data are presented as the deterministic results and the shaded areas indicate the 95% confidence interval of the estimates.

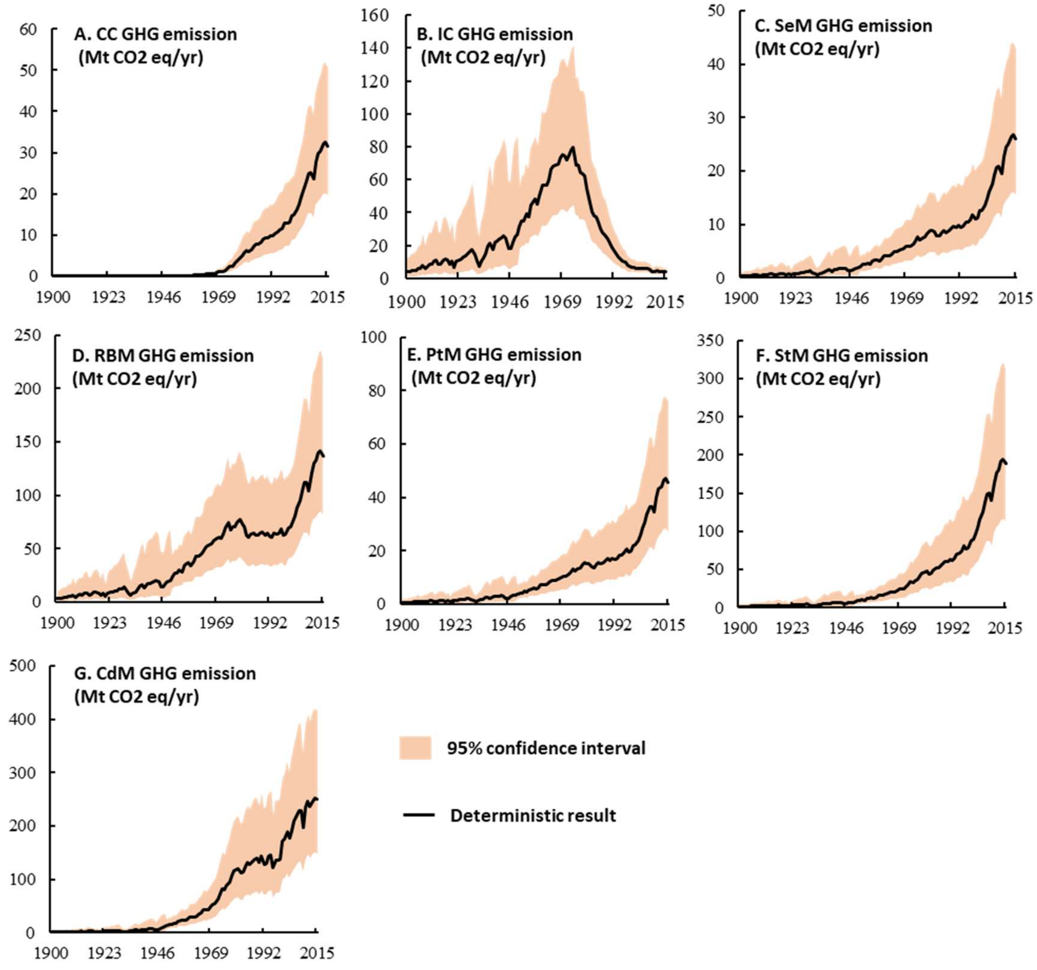

Fig. S3.6 GHG emission for casting and finishing processes. Data are presented as the deterministic results and the shaded areas indicate the 95% confidence interval of the estimates.

S3.3 Results evaluation

This sub-section compares our results with other studies. Notably, most of the material flow results (in Fig. S3.1-3.2) in the material production stage is directly obtained from official statistics or mass balance. Hence, the accuracy of those results can be guaranteed. Herein, the Pearson correlation is adapted for comparing the production activities data in Fig. S3.7 and this study compared the results in other life cycle stages (i.e. in-use stock, scrap, etc.) with other studies, as shown in Table S3.1. Furthermore, the greenhouse gas emission of our study is compared with other published ones in Fig. S3.8.

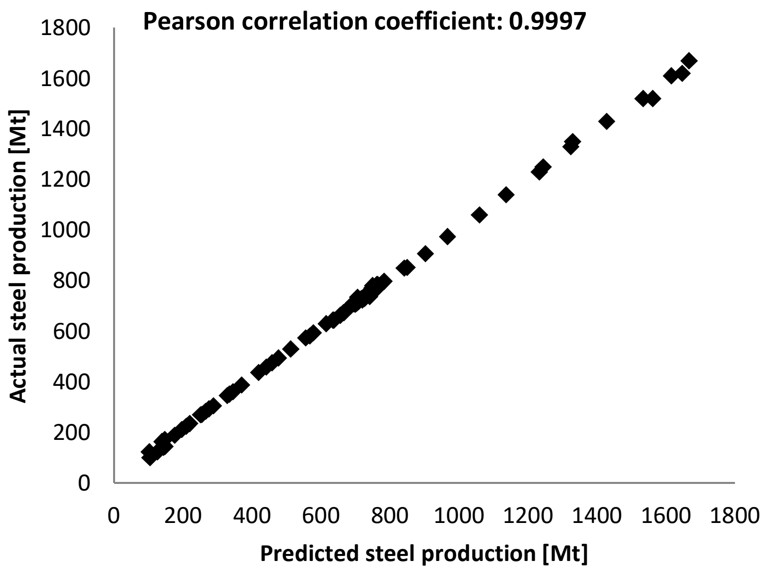

Fig. S3.7 Scatterplot showing correlation between predicted steel production amounts and actual production amounts based on USGS [13] between 1943 and 2015.

Table S3.1 Comparison of our results related to in-use stock and scrap with others

Content	Time	Value	Time	Value	Time	Value
In-use stock (Gt) - Other studies	2000	~20 [3]<br>14.9 [72]<br>10.8 [73]	2005	~21[3]<br>17.8 [74]<br>17.0 [72]	2008	~23 [3]<br>18.9 [72]
In-use stock (Gt)- this study	2000	15.8±0.7	2005	18.0±0.8	2008	20±1
Old scrap (Mt/yr)-other studies	1980	~170[73]<br>~150 [3]<br>100 [75]	2000	~305 [73]<br>~300 [3]<br>257 [4]	2008	~400 [3]<br>~370 [2]
Old scrap (Mt/yr)-this study	1980	159±30	2000	289±51	2008	342±60
EoL RR- other studies	1920	~22% [75]	2008	83% [76]<br>80% [2,29]<br>53.5% [72]	Average in the studied period	53% [72]<br>41%-74% [38]<br>~55% [75]<br>39% [77]
EoL RR- this study	1920	16%±3%	2008	79%±15%	Same	45.8%±8%

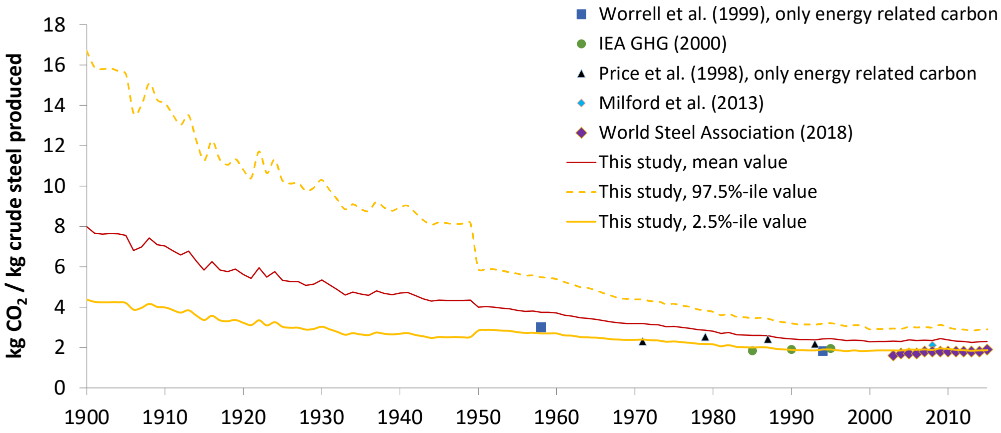

Fig. S3.8 Comparison of estimated GHG intensities in this study with other GHG intensities reported in literature [48,78–81]

S3.4 GHG intensity results

The GHG intensity of each production routes are presented in **Fig. S3.9**.

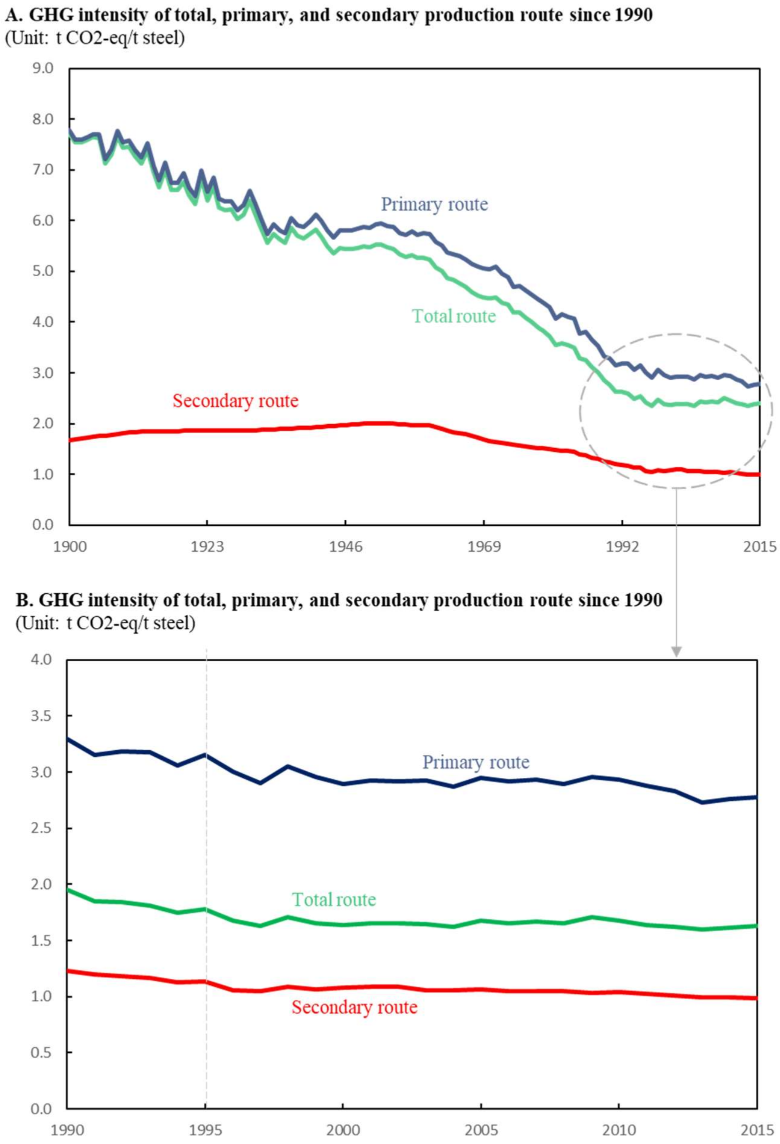

Fig. S3.9 GHG intensity of each production route during the period 1900 -2015(A), and 1990-2015 (B)

S4. Additional results and figures

S4.1 The share of GHG emission from steel production from 1990 to 2015

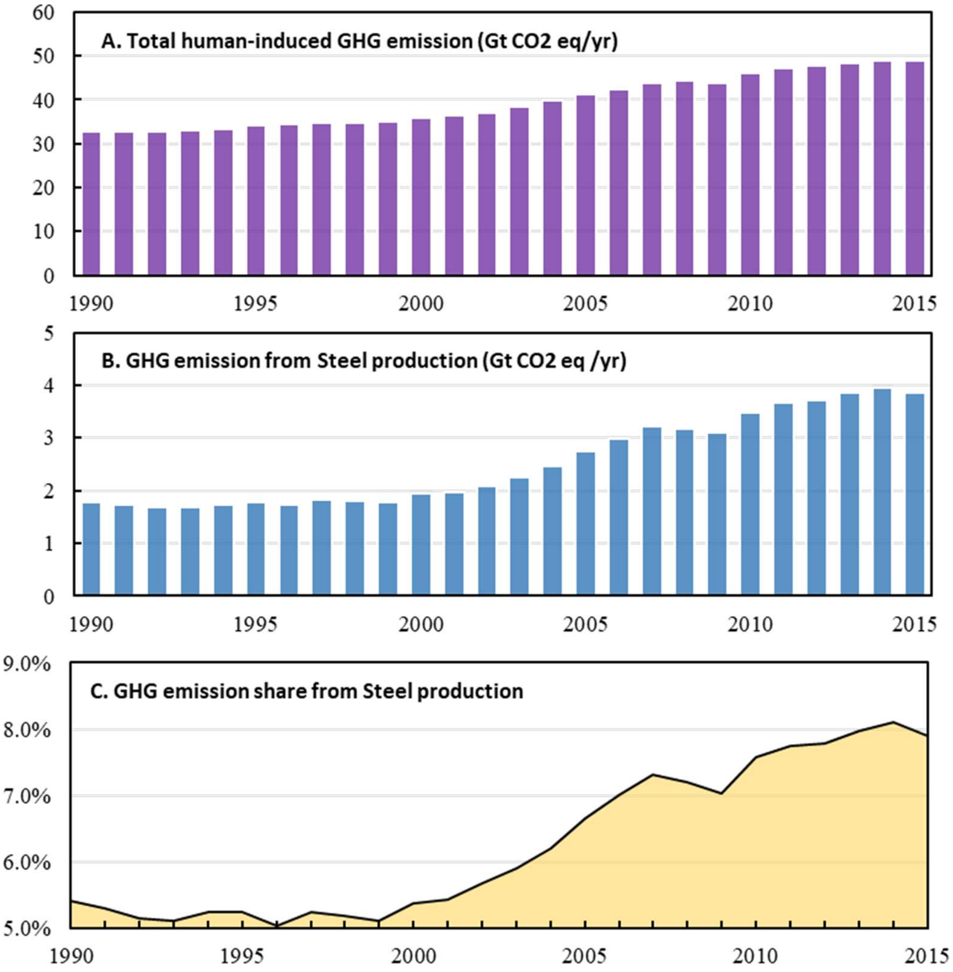

Fig. S4.1 The total global GHG emission and that from steel production and its share change

S4.2 Primary and secondary production and emission data

(1) The production and emission from primary and secondary production

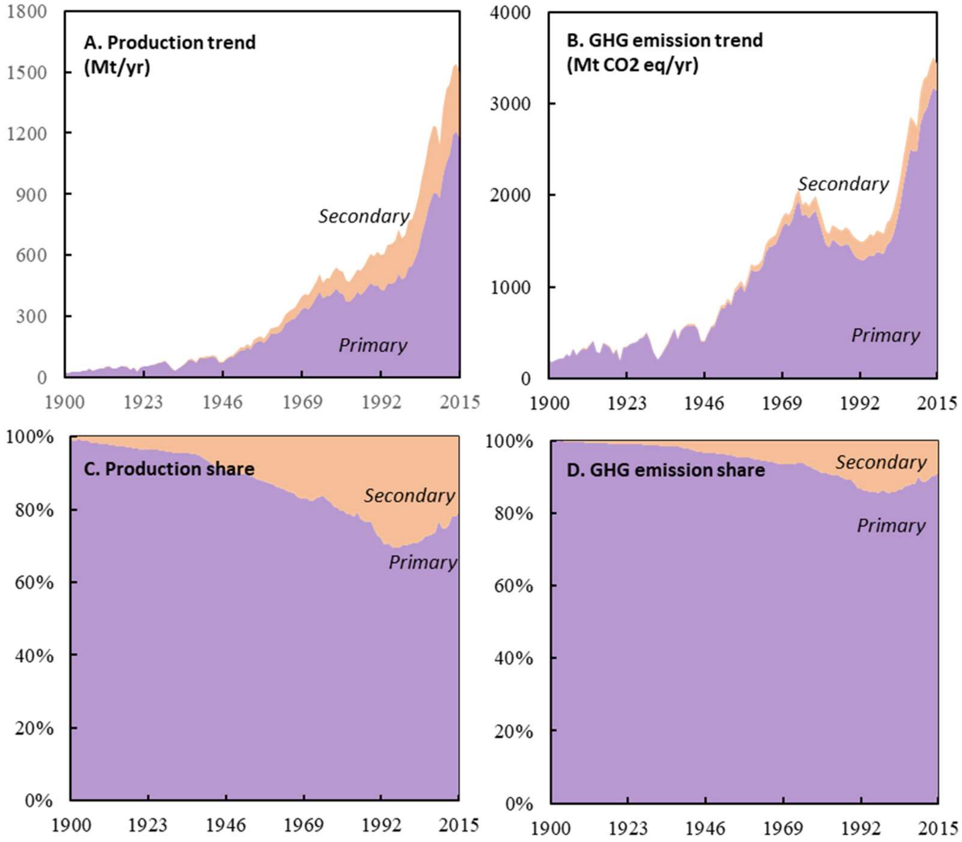

Fig. S4.2 Production and emission from primary and secondary production

(2) Decomposition analysis of driving factors in emission change

We firstly applied the LMDI decomposition method to clarify the contribution of volume and intensity on the absolute emission change, and the method is described in the following equations. The total emission (E) is equal to the volume (V) times intensity (I), and then the change of total emission from t-Δt to t can be decomposed into these two factors as follows:

$$E(t) = \sum V(t) \times \frac{E(t)}{V(t)} = \sum V(t) \times I(t) \qquad \text{(S6)}$$

$$\Delta E_{total} = E(t) - E(t - \Delta t\ ) = \Delta E_{volume} + \Delta E_{intensity} \qquad \text{(S7)}$$

Where the absolute change of those three factors are:

$$\Delta E_{total} = \sum w(t) \times \ln(\frac{E(t)}{E(t-\Delta t\ )}) \qquad \text{(S8)}$$

$$\Delta E_{volume} = \sum w(t) \times \ln(\frac{V(t)}{V(t-\Delta t\ )}) \qquad \text{(S9)}$$

$$\Delta E_{intensity} = \sum w(t) \times \ln(\frac{I(t)}{I(t-\Delta t\ )}) \qquad \text{(S10)}$$

$$w(t) = \frac{E(t) - E(t-\Delta t)}{\ln E(t) - \ln E(t-\Delta t)} \quad \text{(S11)}$$

To check whether the efficiency (or the intensity) improvement can lead to the absolute reduction of total emission, we further applied the relative index decomposition to trace the interaction of those factors (i.e. emission, volume, intensity, and the negligible interaction of volume and intensity) as follows:

$$\Delta E(t) = I(t) \times \Delta V(t) + V(t) \times \Delta I(t) + \Delta I(t) \times \Delta V(t) \quad \text{(S12)}$$

$$\left(\frac{\Delta E(t)}{E(t)}\right) = \left(\frac{\Delta V(t)}{V(t)}\right) + \left(\frac{\Delta I(t)}{I(t)}\right) + \left(\frac{\Delta V(t)}{V(t)} \times \frac{\Delta I(t)}{I(t)}\right) \quad \text{(S13)}$$

Where each term is the relative emission change (in percent) along the designed periods and such decomposition analysis can eliminate the scale factor and to reflect the contribution of each factor more straight compared to the pervious LMDI approach.

For a relatively substantial investigation, we applied the above decomposition analysis for every five years, when the figures are in a cumulative format in each period. The detailed results are shown in Table S4.1. We further investigated the correlation relationship for those three factors, and the results are shown in Fig.S4.3.

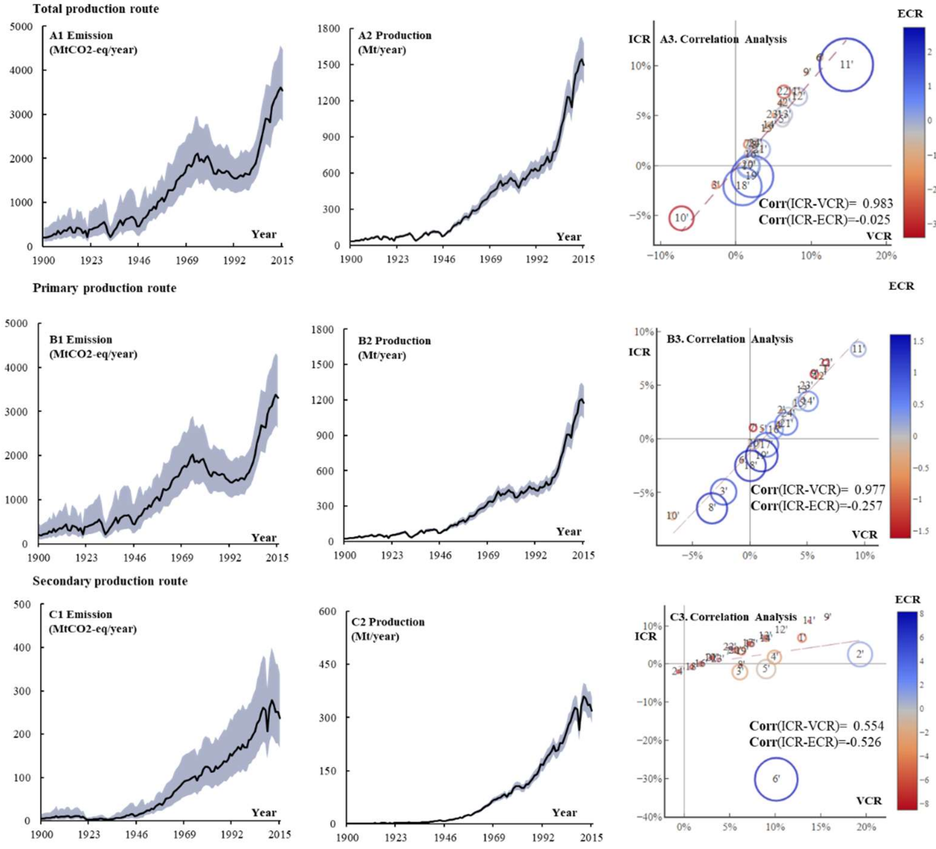

Fig. S4.3 Correlation between volume change (VCR) and efficiency change (ECR) to the Impact change (ICR) Data in A1, A2, B1, B2, C1, C2 shows arithmetic mean of estimates and the shaded areas indicate the 95% confidence interval of the estimates

Table S4.1 The decomposition analysis of GHG emission and its driving factors in steel production

	Period	Cumulative Amount (5 years)			Emission decomposition (Mt CO_2 eq/5 years)			Change rate decomposition (%)			
		Emission (Mt CO_2 eq)	Volume (Mt)	Intensity (t CO_2eq/t)	Emission change	Volume factor	Intensity factor	Emission change	Volume factor	Intensity factor	Cross factor
Total steel production	1900-05	1081.0	143.9	7.5							
	1906-10	1451.6	198.5	7.3	371	404	-33	29.3	31.9	-2.6	0.06
	1911-15	1679.9	239.3	7.0	228	292	-64	14.6	18.7	-4.1	0.03
	1916-20	1704.4	257.2	6.6	24	122	-97	1.4	7.2	-5.8	0.00
	1921-25	1654.4	257.1	6.4	-50	-1	-49	-3.0	0.0	-2.9	0.00
	1926-30	2227.1	363.7	6.1	573	668	-96	29.5	34.3	-5.0	0.13
	1931-35	1458.1	255.8	5.7	-769	-639	-130	-41.7	-34.8	-7.2	0.26
	1936-40	2560.9	453.9	5.6	1103	1123	-20	54.9	55.8	-1.0	0.08
	1941-45	2750.0	500.2	5.5	189	258	-69	7.1	9.7	-2.6	0.00
	1946-50	2787.1	518.1	5.4	37	97	-60	1.3	3.5	-2.2	0.00
	1951-55	4273.5	796.6	5.4	1486	1496	-10	42.1	42.4	-0.3	0.01
	1956-60	5429.1	1047.5	5.2	1156	1322	-166	23.8	27.2	-3.4	0.06
	1961-65	6747.7	1401.1	4.8	1319	1764	-445	21.7	28.9	-7.3	0.11
	1966-70	8336.5	1866.3	4.5	1589	2154	-565	21.1	28.5	-7.5	0.11
	1971-75	9612.0	2281.1	4.2	1275	1798	-522	14.2	20.0	-5.8	0.04
	1976-80	9616.5	2557.2	3.8	5	1099	-1094	0.0	11.4	-11.4	0.00
	1981-85	8278.1	2500.9	3.3	-1338	-199	-1139	-15.0	-2.2	-12.7	0.01
	1986-90	8082.5	2857.7	2.8	-196	1091	-1287	-2.4	13.3	-15.7	-0.01
	1991-95	7627.3	3135.2	2.4	-455	728	-1183	-5.8	9.3	-15.0	-0.02
	1996-00	8046.0	3551.5	2.3	419	977	-558	5.3	12.5	-7.1	0.01
	2001-05	10194.9	4526.7	2.3	2149	2203	-54	23.6	24.1	-0.6	0.01
	2006-10	14167.8	6080.8	2.3	3973	3563	409	32.6	29.3	3.4	-0.08
	2011-15	16936.4	7427.6	2.3	2769	3103	-334	17.8	19.9	-2.2	0.02
Primary steel production	1900-05	1078.4	142.4	7.6							
	1906-10	1445.1	194.8	7.4	367	393	-26	29.1	31.1	-2.1	0.05
	1911-15	1669.2	233.5	7.1	224	281	-57	14.4	18.0	-3.7	0.02
	1916-20	1690.1	249.5	6.8	21	111	-90	1.2	6.6	-5.4	0.00
	1921-25	1637.8	248.1	6.6	-52	-9	-44	-3.1	-0.5	-2.6	0.00
	1926-30	2200.7	349.5	6.3	563	652	-89	29.3	33.9	-4.7	0.12
	1931-35	1436.4	244.2	5.9	-764	-642	-122	-42.0	-35.4	-6.8	0.25
	1936-40	2514.5	429.6	5.9	1078	1087	-9	54.6	55.0	-0.5	0.04
	1941-45	2672.2	460.2	5.8	158	179	-21	6.1	6.9	-0.8	0.00
	1946-50	2687.5	467.9	5.7	15	44	-29	0.6	1.7	-1.1	0.00
	1951-55	4103.1	711.1	5.8	1416	1400	15	41.7	41.3	0.5	-0.02
	1956-60	5171.6	916.6	5.6	1069	1172	-103	23.0	25.2	-2.2	0.03
	1961-65	6371.4	1198.6	5.3	1200	1543	-343	20.8	26.7	-6.0	0.08

	Period	Cumulative Amount (5 years)			Emission decomposition (Mt CO_2 eq/5 years)			Change rate decomposition (%)			
		Emission (Mt CO_2 eq)	Volume (Mt)	Intensity (t CO_2eq/t)	Emission change	Volume factor	Intensity factor	Emission change	Volume factor	Intensity factor	Cross factor
	1966-70	7805.4	1556.6	5.0	1434	1846	-412	20.2	26.0	-5.8	0.08
	1971-75	8995.1	1895.6	4.7	1190	1652	-462	14.2	19.6	-5.5	0.04
	1976-80	8879.9	2071.7	4.3	-115	794	-909	-1.3	8.9	-10.2	0.00
	1981-85	7512.6	1971.9	3.8	-1367	-404	-963	-16.7	-4.9	-11.8	0.02
	1986-90	7212.5	2185.4	3.3	-300	757	-1057	-4.1	10.3	-14.3	-0.02
	1991-95	6582.3	2234.6	2.9	-630	154	-784	-9.1	2.2	-11.4	-0.01
	1996-00	6906.9	2485.0	2.8	325	716	-392	4.8	10.6	-5.8	0.01
	2001-05	8807.3	3230.5	2.7	1900	2051	-151	24.2	26.1	-1.9	0.03
	2006-10	12535.7	4519.0	2.8	3728	3545	183	34.9	33.3	1.7	-0.05
	2011-15	15224.8	5721.3	2.7	2689	3264	-575	19.4	23.5	-4.2	0.05
Secondary steel production	1900-05	2.6	1.5	1.7							
	1906-10	6.5	3.6	1.8	3.9	3.7	0.2	85.8	82.1	4.5	-0.79
	1911-15	10.7	5.8	1.8	4.2	4.0	0.2	48.4	46.6	2.0	-0.11
	1916-20	14.3	7.7	1.9	3.6	3.5	0.1	28.9	28.2	0.7	-0.01
	1921-25	16.6	8.9	1.9	2.3	2.3	0.1	15.1	14.7	0.4	0.00
	1926-30	26.5	14.2	1.9	9.9	9.8	0.0	45.8	45.6	0.2	-0.01
	1931-35	21.7	11.5	1.9	-4.8	-5.1	0.3	-20.0	-21.0	1.1	-0.01
	1936-40	46.4	24.3	1.9	24.7	24.2	0.5	72.6	71.3	1.5	-0.19
	1941-45	77.7	40.0	1.9	31.4	30.2	1.1	50.6	48.8	1.8	-0.11
	1946-50	99.7	50.1	2.0	22.0	20.0	2.0	24.8	22.5	2.2	-0.03
	1951-55	170.4	85.5	2.0	70.7	70.4	0.4	52.4	52.1	0.3	-0.02
	1956-60	257.5	130.9	2.0	87.1	90.0	-2.9	40.7	42.0	-1.4	0.06
	1961-65	376.3	202.6	1.9	118.8	136.7	-17.9	37.5	43.0	-5.7	0.23
	1966-70	531.1	309.7	1.7	154.7	190.7	-36.0	34.1	41.8	-8.0	0.29
	1971-75	616.9	385.5	1.6	85.8	125.4	-39.6	14.9	21.8	-6.9	0.06
	1976-80	736.6	485.5	1.5	119.7	155.7	-36.0	17.7	23.0	-5.3	0.05
	1981-85	765.5	529.1	1.4	28.9	64.5	-35.6	3.8	8.6	-4.7	0.00
	1986-90	870.0	672.3	1.3	104.5	195.7	-91.2	12.8	23.9	-11.2	0.09
	1991-95	1045.0	900.6	1.2	175.0	279.1	-104.1	18.3	29.0	-10.9	0.14
	1996-00	1139.1	1066.4	1.1	94.2	184.5	-90.3	8.6	16.9	-8.3	0.03
	2001-05	1387.6	1296.1	1.1	248.5	245.6	2.9	19.7	19.4	0.2	0.00
	2006-10	1632.0	1561.9	1.0	244.4	281.0	-36.6	16.2	18.6	-2.4	0.02
	2011-15	1711.6	1706.3	1.0	79.6	147.8	-68.2	4.8	8.8	-4.1	0.00

S4.3 Regional material and environmental performance analysis

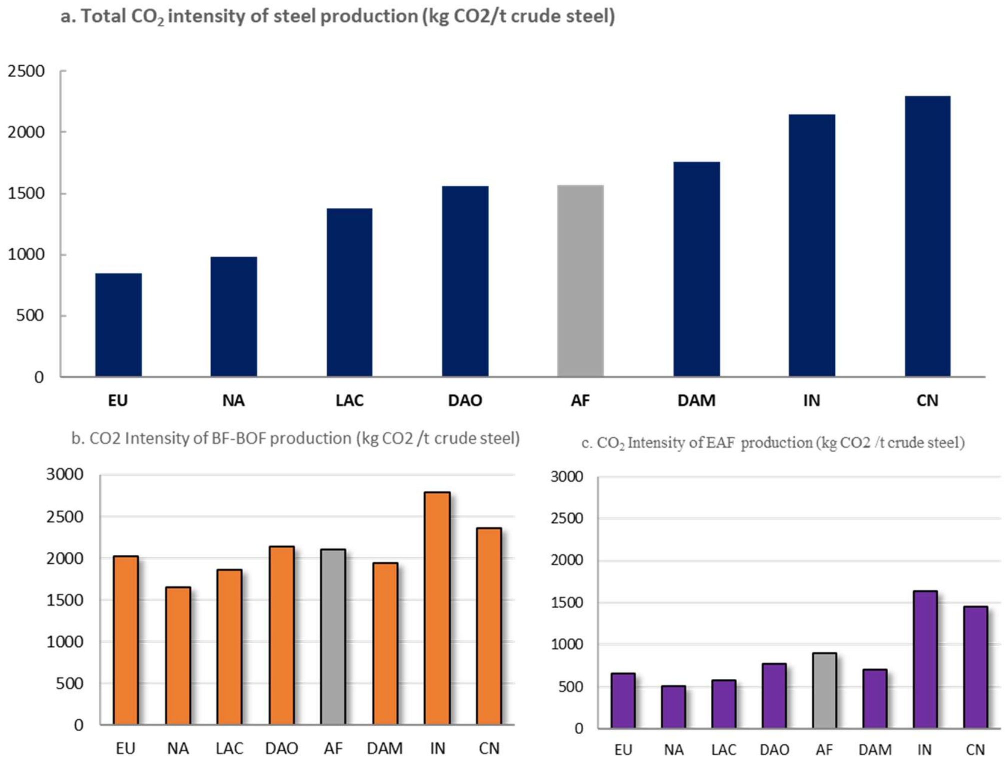

Fig. S4.4 The carbon intensity of different production route in different regions in 2016 (National results are from Global Efficiency Intelligence's benchmark study [82], and we use the average of the representative nations to represent the indicative level for each region, i.e., EU (Europe)- Spain, Italy, Poland, Germany, and French; NA(North America)-USA, Canada; LAC (Latin America and Caribbean)-Brazil and Mexico; DAO(Developed Asia and Oceania)- Japan and South Korea; DAM (Developing Asia and Middle East)- Turkey; IN-India; CN-China; AF-Africa (No national investigation, presented by global average level))

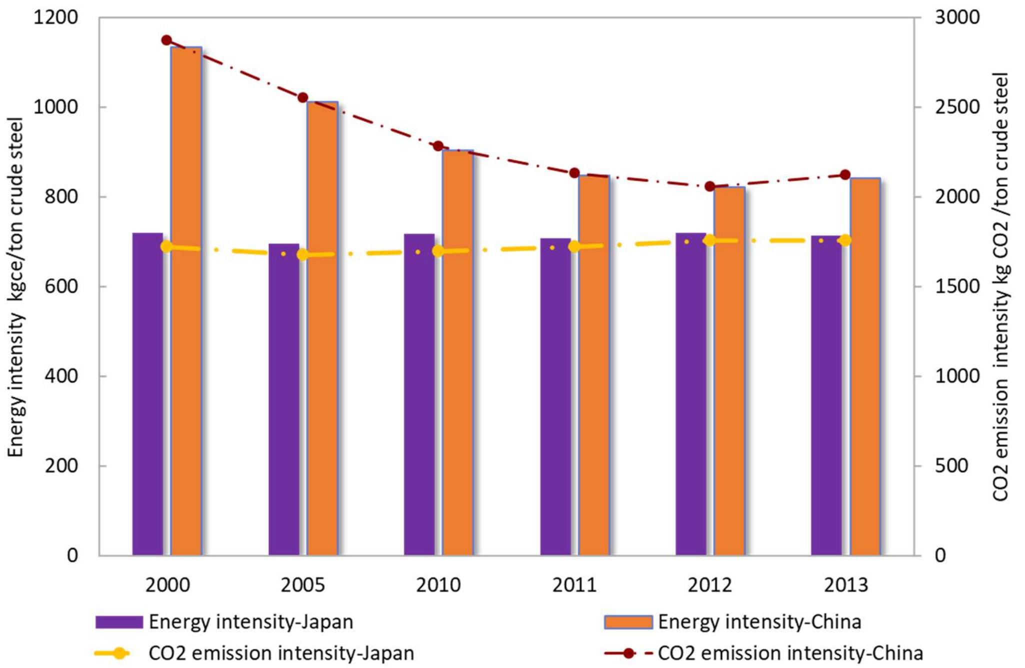

Fig. S4.5 Historical CO_2 intensity trend of China and Japan (Data from [83])

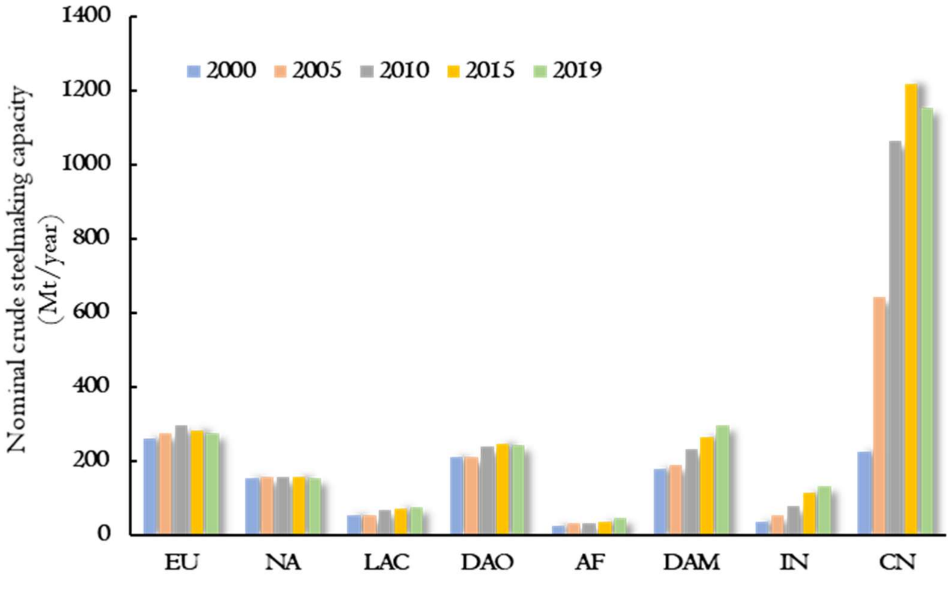

Fig. S4.6 The nominated regional crude steel production capacity (Data from [84], EU-Europe; NA-North America; LAC-Latin America and Caribbean; DAO-Developed Asia and Oceania; DAM -Developing Asia and Middle East; IN-India; CN-China; AF-Africa)

S4.4 Settings of future scenarios

We propose six scenario sets to indicate the future pathways of required technical and material efficiency improvement to follow the 1.5DS pathway, the details of which are described in Table S4.2 and Fig. S4.6.

Table S4.2 Settings and Results of proposed future scenarios

Scenario	Settings	Implications
1. Efficiency stagnation scenario	• Production trend from IEA Stated Policy Scenario (71 Gt during 2019-50)<br>• Efficiency trend keeps stagnating	The remaining carbon budget will be extracted by year ~2033
2. Technical efficiency improvement	• Production trend from IEA Stated Policy Scenario (71 Gt during 2019-50)<br>• Emission efficiency follows the annual rate of IEA Sustainable Development Scenario (0.71 CO_2-eq/t by 2050)	The remaining carbon budget will be extracted by year ~2037
3. Material efficiency improvement	• Production trend from IEA Sustainable Development Scenario (64 Gt during 2019-50)<br>• Emission efficiency follows the annual rate of IEA Sustainable Development Scenario (0.71 CO_2-eq/t by 2050)	The remaining carbon budget will be extracted by year ~2038
4. Budget-constrained technical efficiency scenario	• Production trend from IEA Stated Policy Scenario (71 Gt during 2019-50)<br>• Not extract the 1.5DS budget by 2050	Radical GHG intensity decrease to zero by 2046 at an average rate of 0.85 t CO_2-eq/t steel per decade
5. Budget-constrained material efficiency scenario	• Emission efficiency follows the annual rate of IEA Sustainable Development Scenario (0.71 CO_2-eq/t by 2050)<br>• Not extract the 1.5DS budget by 2050	If GHG intensity decrease to 0.71 t CO_2-eq/t by 2050 (similar to scenario 3), and the total demand should be cut by additional 34% (43 Gt during 2019-50) compared to IEA Sustainable Development Scenario

As for future demand trends in Fig.S4.6a, IEA [85] has published their estimates of steel production from 2011 to 2050 with two scenarios – stated policy scenario and sustainable development scenario. The stated policy scenario describe future steel demand under the business as usual. With the implementation of material efficiency strategies, the future steel demand can be reduced while maintaining the same service, and the projection is presented in sustainable development scenario.

As for the carbon budget estimation under IEA 1.5DS, we obtained this level based on the

efficiency trend from IEA 1.5DS (i.e. decreasing from 2.38 t CO_2-eq/t in 2010 to 0.32 t CO_2-eq/t in 2050 in Fig. 4.7b). Under this efficiency condition, the highest demand scenario (i.e. stated policy scenario) in Fig.S4.7a is chosen to obtain the high variance of such a carbon budget, totaling 106 Gt CO_2-eq from 2010 to 2050. One recent study [86] has reviewed the existing framework and amounts of the carbon budget for all human activities after 2018 to hold warming to 1.5 °C above pre-industrial levels (50% chance), which stays 420–580 Gt CO_2-eq. Given the steel sector only accounts for 7-9% of global GHG emission, the carbon budget of 106 Gt CO_2-eq should be halved according to this allocation (18%-28%), indicating a more stringent carbon constraint on future steel production. The GHG emission pathways under our proposed scenarios are shown in Fig. S4.7c.

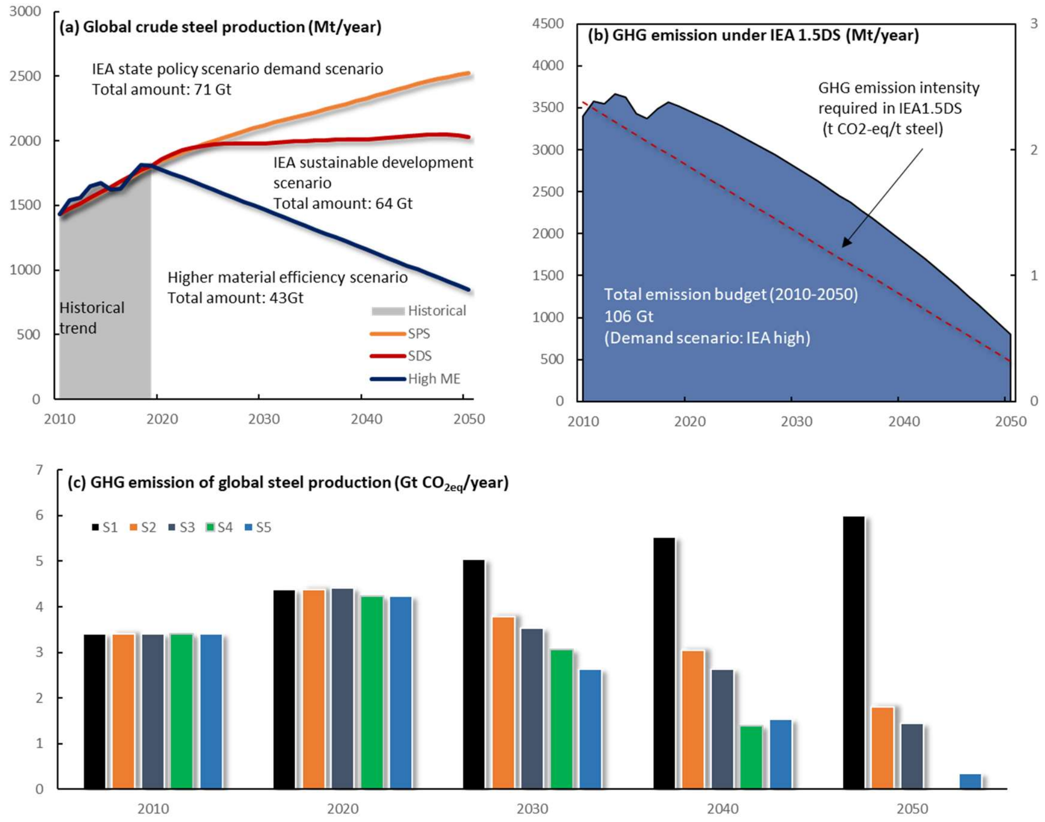

Fig. S4.7 Potential trend of future efficiency and emission trend under proposed scenarios

S4.5 Historical measures to improve steel production efficiency

This subsection summarized the national policies in decarbonizing steel and other energy-intensive industries from the IEA policy and measure databases in Table S4.3, and most international emissions reduction policies were linked to efficiency, energy use, and carbon within production sites. There is little apparent attention from those policies focusing on the role of material life cycle on the carbon reduction in production stage.

Table S4.3 The current national policies related to decarbonization of steel and other EIIs

Measures	Nation	Time	Focus	Life cycle stage	Details
Carbon Pricing Mechanism	Australia	2012-14	Carbon pricing	Production	Operating facilities with annual emissions >25kt GHG emissions
Energy Efficiency Opportunities	Australia	2006-14	Energy efficiency	Production	Businesses with annual energy use > 0.5PJ.
Clean Technology Program	Australia	2012-14	Technologies promotion	Production	Businesses in all sectors to invest in clean energy and reduce emissions, especially energy efficient and low emissions technologies.
Energy Efficiency Standards and Labelling	Canada	1995-	Energy efficiency	Production	Energy Efficiency Regulations for Minimum Energy Performance Standards
Industry Program for Energy Conservation	Canada	1975-	Energy efficiency	Production	Industrial energy efficiency and reduce GHG emissions from energy use in the industrial sector.
ISO 50001 implementation Support	Canada	2012-	Energy audit	Production	Economic assistance to industrial companies to perform ISO 50001 implementation pilots and energy related assessments
Accelerated capital cost allowances	Canada	1994-2020	Technologies promotion	Production	Deductions on capital expenditures on the cost of the asset over the asset's useful life
Integrated Pollution Prevention and Control	Finland	2000-	Cleaner production	Production	Appropriate controls for industry to control levels on energy use and CO_2 emissions
Energy Efficiency Agreements	Finland	1997-2016	Energy efficiency	Production	Encourage industry to improving their energy efficiency, implement an Energy Efficiency System, implement the measures necessary to reach their targets, and report annually
Energy Audit Program	Finland	1992-	Energy audit	Production	Voluntary program supported by a 40% to 50% subsidy for energy saving
Energy-saving target	Germany	2013-22	Energy efficiency	Production	Voluntary agreement with German industry to reduce industry's greenhouse gas emissions
Tax incentives	Germany	1999-2022	Technologies promotion	Production	Rebate on energy and electricity tax for energy intensive companies if they fulfil their targets under the voluntary agreements.
Keidanren Voluntary Action Plan	Japan	1997-2012	Efficiency	Production	It allowed industry groups to choose among four types of indicators on which to base their target: energy consumption, energy intensity, CO_2 absolute emission, or CO_2 intensity
Mandatory GHG reporting	Japan	2005-	GHG reporting	Production	To calculate their GHG emissions and report the results to the Government
Mandatory energy efficiency benchmarking in industry	Japan	1978-	Energy benchmarking	Production	Rational Use of Energy
Mandatory Energy Audits for Large Power Consumers	Korea	2007	Energy audit	Production	Mandatory energy audit program for energy-intensive companies
Long-term Agreement on Energy Efficiency for EU ETS enterprises	Netherlands	2009-2020	Energy efficiency	Production	To promote energy savings in industry specifically for enterprises that participate in the EU Emissions Trading System

Measures	Nation	Time	Focus	Life cycle stage	Details
Energy Investment Allowance	Netherlands	1997	Technologies promotion	Production	A direct financial advantage to companies that invest in energy-saving equipment and sustainable energy.
Technology Procurement	Sweden	1990-	Technologies promotion	Production	To stimulate and accelerate the development of new technologies
Energy Audit Financial support	Sweden	2010-14	Technologies promotion	Production	Financial support for energy audits
Improving Energy Efficiency in Energy Intensive Industries	Sweden	2005-17	Energy efficiency	Production	To increase energy efficiency in energy-intensive industries
Environmental Tax on Fuels	Sweden	1991-2005	Carbon pricing	Production	Environmental taxes are levied on fuels based on their content of carbon
Support scheme for EE in industry	Turkey	2008	Technologies promotion	Production	To support up to 30% of the total costs of energy efficiency projects
Climate Change Agreement	United Kingdom	2001-23	Energy efficiency	Production	To achieve energy savings and energy efficiency improvements energy-intensive industry while protecting their competitiveness
Enhanced Capital Allowance Scheme	United Kingdom	2001-	Technologies promotion	Production	To encourage businesses to invest in low carbon, energy-saving equipment.
Greenhouse Gas (GHG) reporting	United States	2010	GHG reporting	Production	To requires facilities that emit 25,000 tons of CO_2e or more per year from on-site combustion to annually report their emissions to the Environmental Protection Agency.
Clean Air Act	United States	2001	Cleaner production	Production	Large industrial installations are required to obtain preconstruction permits for GHG emissions as well as for other air pollutants
Better Buildings Better Plants	United States	2011	Energy efficiency	Production	To reduce the energy intensity of industrial operations by 25% or more in 10 years.
Superior Energy Performance	United States	2013	Energy efficiency	Production	To provide industrial facilities with a roadmap for achieving continual improvement in energy efficiency while maintaining competitiveness.
Industrial Energy Performance Standards	China	2008	Energy efficiency	Production	Industrial energy performance standards set minimum allowable energy efficiency values for existing plants and newly constructed plants
Energy Efficiency Appraisals for New Large Industrial Projects	China	2010-15	Energy efficiency	Production	Energy Efficiency Appraisals for New Large Industrial Projects
Small Plant Closures and Phasing Out of Outdated Capacity	China	2007-15	Outdated Capacity Phasing Out	Production	To accelerate the closing of small plants and phasing out of outdated capacity in 14 high energy-consumption industries.
EE Financing Regulations and Instruments	China	2007-15	Energy efficiency	Production	To support the achievement of the national energy intensity reduction targets
Financial Rewards for Energy-Saving Technical Retrofits	China	2007-15	Technologies promotion	Production	To reward enterprises for energy savings achieved through technical renovation.
National Energy Conservation Awards	India	1991-	Energy efficiency	Production	To promote energy efficiency and the adoption of clean and innovative technologies in industrial and other sectors
Mandatory Energy Audits and Energy Managers	India	2001-	Energy audit	Production	It is mandatory for all the designated energy consumers to have energy audits.
Financing Scheme IREDA	India	1987	Energy efficiency	Production	To promote, develop and extend financial assistance for renewable energy and energy efficiency/conservation projects
Federal law on energy conservation and energy efficiency	Russia	2009-20	Cleaner production	Production	To reduce the intensity of electricity, heat, water and gas consumption
Tax reforms for Energy Efficiency improvements	Russia	2001	Technologies promotion	Production	Promotion of investments in R&D and energy efficient equipment
National Energy Efficiency Leadership Network	South Africa	2005-15	Energy efficiency	Production	An improvement in energy intensity of 1% per annum for the iron and steel Industry.
Tax Incentives	Thailand	2006	Technologies promotion	Production	Tax incentives for energy efficiency projects.

S4.6 Breakthrough low-carbon steel production technologies

Table S4.4 summarizes the very low or zero carbon breakthrough steel production technologies. The searching of the category of low-carbon production technologies is on the basis of IEA's ETP Clean Energy Technology Guide [87], publications [88], and expert's consultation, and the details of each technology is investigated and updated based on their official website and technical reports, which are cited in Table S4.4. Besides, each technology is marked according to their technology readiness levels using NASA's commonly used Technology Readiness Level (TRL) scale method (TRL 1 – Basic principles observed; TRL 2 – Technology concept formulated; TRL 3 – Experimental proof of concept; TRL 4 – Technology validated in lab; TRL 5 – Technology validated in relevant environment; TRL 6 – Technology demonstrated in relevant environment; TRL 7 – System prototype demonstration in operational environment; TRL 8 – System complete and qualified; TRL 9 – Actual system proven in operational environment).

Table S4.4 Summary of breakthrough low-CO_2 steel production technologies

No.	Project	Type	Company	Implementation Region	Principle	Principle-details	Stage	Potential	Implementation time	Source
**Hydrogen-based pathway**										
1	HYBRIT	Carbon-free primary production technology	SSAB/LKAB/Vattenfall	Sweden	Hydrogen-based pathway<br>(with hydrogen from renewables)	Using hydrogen (instead of coal) for the direct reduction of iron oxide/ore (H-DR), combined with an electric arc furnace (EAF).<br>Reducing agent used-the main source of hydrogen is the electrolysis of water to produce hydrogen.<br>The electricity used in electrolysis of water comes from clean energy power stations such as water power and wind power.	TRL=5-7	99% or zero GHG emission	In pilot phase at present<br>Construction of demonstration plant and trial operation; to have a carbon-free iron smelting by 2035 solution.<br>2045 available.	[89]

No.	Project	Type	Company	Implementation Region	Principle	Principle-details	Stage	Potential	Implementation time	Source
2	SALCOS	Carbon-free primary production technology	Salzgitter AG /Fraunhofer Institute/Tenova/Dillingen and Saarsteel	Germany	Hydrogen-based pathway (with hydrogen from renewables)	Hydrogen-based DRI-EAF steelmaking linked to the GrInHy project for production of green industrial hydrogen.	TRL=6-7	26-95% (-26% CO_2 reduction; -82% CO_2 if operated with 55% H2; -95% CO_2 if operated with 100% H_2)	In February 2019, the GrInHy1.0 project was completed. Further development dependent on government policy, and the political framework conditions and economic efficiency criteria	[90,91]
3	Carbon2Chem project	Carbon-free primary production technology	ThyssenKrupp	Germany	Hydrogen-based pathway (within BF and then DRI)	Replace coal with H_2 as a reducing agent in BF and then DRI to reduce CO_2 emissions from steel production. Based on utilization of industrial waste gases, aiming to use smelting gases for chemicals production (e.g. methanol)	TRL=3-6	max-50% reduction	2025-2030 available	[92]
4	Renewable-based hydrogen steel making factory	Carbon-free primary production technology	HBIS Group /Tenova	China	Hydrogen-based pathway (with hydrogen from renewable)	To develop a new type of steel metallurgical production process with hydrogen energy as the core, and use renewable green energy to build a 1.2 million tons of hydrogen metallurgical demonstration project production line. Aim to be the first and the largest real-life plant using hydrogen-based technology at an industrial production scale.	TRL=5~6	~100% CO_2 reduction	N.A. Memorandum of understanding signed at the end of 2019	[93]
5	Coal-based hydrogen metallurgy pilot plant	Carbon-reduction primary production technology	Jianlong Group	China	Hydrogen-based pathway (with hydrogen from coke oven gas)	Smelting reduction plant which uses a mixture of hydrogen and coal.	TRL=7	N.A.	First production in October 2020	[94]

No.	Project	Type	Company	Implementation Region	Principle	Principle-details	Stage	Potential	Implementation time	Source
6	Natural gas-based hydrogen metallurgy pilot plant	Carbon-reduction primary production technology	Rizhao Steel	China	Hydrogen-based pathway (with hydrogen from coke oven gas)	The project will produce 0.5 Mt/a DRI using hydrogen, which is extracted from the co-products of a natural gas-based process to make vinyl acetate.	TRL=7	N.A.	Launched in early May 2020, Production data unknown	[94]
7	Coal-based hydrogen metallurgy pilot plant	Carbon-reduction primary production technology	Jiu Steel Group	China	Hydrogen-based pathway (with hydrogen from coal)	Build the world's first coal-based hydrogen metallurgy pilot plant and supporting dry mill and dry selection test plant.	TRL=3-5	50% CO_2 reduction	N.A. Established the Hydrogen Energy Research Institute in September 2019.	[95]
8	COOLSTAR	Carbon-free primary production technology	Ministry of Trade, Industry and Energy	Korea	Hydrogen-based pathway (with hydrogen from by-product gas)	COOLSTAR (CO_2 Low Emission Technology of STeelmaking and Hydrogen Reduction), the traditional blast furnace that uses coal as energy is used as the basis to make full use of "ash hydrogen". This type of hydrogen is mainly by upgrading and refining the by-product gas produced by iron and steel plants	TRL=2-5	The ultimate goal is to reduce CO_2 15%	Commercialization will be realized around 2050	[96]
9	MIDREX H2	Carbon-free primary production technology	MIDREX	USA	Hydrogen-based pathway (with hydrogen from renewables)	Similar to the standard MIDREX Process except that the H2 input gas is generated external to the process. Thus, there is no reformer and a gas heater is employed to heat the gas to the required temperature.	TRL=2-5	Compared with the blast furnace process, this process can reduce CO_2 emissions by about 80%	N.A.	[97]

No.	Project	Type	Company	Implementation Region	Principle	Principle-details	Stage	Potential	Implementation time	Source
10	H2Future	Carbon-free primary production technology	Voestalpine/Siemens/Verbund/Austria Grid (APG)/Austria K1-MET Center Group	Europe	Hydrogen-based pathway (with hydrogen from renewables)	Hydrogen-based direct reduction technology uses hydrogen instead of carbon in basic steelmaking, and requires the use of green hydrogen (hydrogen generated from renewable energy power generation through water electrolysis)	TRL=5-6	The ultimate goal is to reduce CO_2 emissions by 80% by 2050.	On 2019, the planned 6 MW electrolysis hydrogen production unit of the Austrian Linz voestalpine steel plant was put into operation, and the era of hydrogen energy metallurgy officially began.	[98]
11	SuSteel	Carbon-free primary production technology	K1-MET GmbH, voestalpine, Montanuniversität Leoben	Europe	Hydrogen-based pathway (with hydrogen from renewables)	The technology is based on the usage of hydrogen plasma. Thereby, hydrogen is used as the reduction agent for the iron ore while its plasma state offers the thermal energy for melting the metallurgical iron. The utilisation of hydrogen as the reduction agent inheres the advantage that only gaseous water remains as by-product.	TRL=3-4	the usual emissions of CO_2 can be fully avoided.	First lab scale process with a capability of around 100g melt was established and operated at Montanuniversity Leoben.	[99]
12	Nuclear hydrogen steelmaking (NHS)	Carbon-free primary production technology	13 domestic companies including KAERI. /POSCO	Korea	Hydrogen-based pathway (with hydrogen from nuclear power)	Hydrogen reduction ironmaking as a national core industrial technology for development. As early as 2009, the Korea Atomic Energy Research Institute and POSCO and other 13 domestic companies and agencies in South Korea signed a nuclear hydrogen cooperation agreement to carry out nuclear hydrogen production information exchange and technology research and development.	TRL=2-5	~100% reduction	2025 test<br>2040: 12 plants application	[100]

No.	Project	Type	Company	Implementation Region	Principle	Principle-details	Stage	Potential	Implementation time	Source
13	Nuclear energy-hydrogen production - metallurgical coupling	Carbon-free primary production technology	Baowu Group/China National Nuclear Corporation /Tsinghua University	China	Hydrogen-based pathway (with hydrogen from nuclear power)	Carry out the research and development of ultra-high temperature gas-cooled reactor nuclear energy hydrogen production, and coupled with steel smelting and coal chemical processes to achieve ultra-low CO_2 emissions and green manufacturing.	TRL=1-3	100% CO_2 reduction	N.A. Strategic Cooperation Framework Agreement Signed the on January 15, 2019.	94
14	Novel Flash Technology	Carbon-reduction primary production technology	American Iron & Steel Institute (AISI)	USA	Hydrogen-based pathway	This technology reduces iron ore concentrate in a flash reactor with a suitable reductant gas such as hydrogen or natural gas, and possibly bio/coal gas or a combination thereof. It is the first flash ironmaking process. This technology is suitable for an industrial operation that converts iron ore concentrate (less than 100 microns) to metal without further treatment.	TRL=2-5	emit a lower amount of CO_2, depending on the source of hydrogen.	N.A.	101
**Electrolysis-based pathway**										
15	IDERWIN (or ULCOWIN)	Carbon-free primary production technology	ArcelorMittal	Europe	Electrolysis-based pathway	This process, based on the ULCOWIN technology developed since 2004, produces steel by electrolysis without direct CO_2 emissions.	TRL=5-6	99% with very low or zero GHG electricity	2030-2035 available	102
16	Molten Oxide Electrolysis (MOE)	Carbon-free primary production technology	Boston Metal	USA	Electrolysis-based pathway	MOE can produce emissions-free steel with the publication of laboratory results using a cost-effective inert anode, and operations moved into offices in Woburn, Massachusetts in 2012. The first semi-industrial MOE cell was commissioned in 2014.	TRL=5-6	99% with zero GHG electricity	2030-2035 available	103
**CCUS with direct/smelting reduction pathway**										

No.	Project	Type	Company	Implementation Region	Principle	Principle-details	Stage	Potential	Implementation time	Source
17	DRI with CCS	CCS-assisted primary production technology	Al Reyadah/Emirates Steel	United Arab Emirates (Middle East)	CCS pathway with direct reduction (DR)	Capturing carbon dioxide from the flue gas of an Emirates Process of Direct Reduced Iron production facility and injecting the CO_2 for enhanced oil recovery (EOR) in the Abu Dhabi National Oil Company's nearby oil fields.	TRL=9	N.A.	Available now for suitable condition (First commercial CCUS project for steel production)	[104]
18	HIsarna with 80-90% CCS	CCS-assisted primary production technology	Tata	Netherlands (small pilot complete)/India (commercially piloted by Tata)	CCS pathway with Coal-based smelting reduction (SR)	Hisarna employs an upgraded smelt reduction process that processes iron ore in a single step, eliminating coke ovens and agglomeration. It is more efficient & produces a concentrated CO_2 stream	TRL=7-8	Up to 80% CO_2 reduction with CCS	2025 available<br>Greenfield commercial plants could be available within 10 years of the completion of the current demonstration project	[105]
19	FINEX with CCS	CCS-assisted primary production technology	POSCO/voestalpine	Korea	CCS pathway with SR ironmaking	The FINEX process, to separate the reduction and melting of iron ore, which can reduce the respective smelting load, and the load borne by the melting part only accounts for about 30% of the blast furnace. The FINEX process also integrates a CO_2 separation system to facilitate the future adoption of carbon capture and storage technology (CCS).	TRL=7-8	3% CO_2 reduction without CCS,<br>Up to 45% CO_2 reduction with CCS	Annual capacity of 2.0 million tons of hot metal commenced operation in January 2014	[106]
20	ULCORED	CCS-assisted primary production technology	LKAB, Voest-alpine and MEFOS	Europe	CCS pathway with DR	ULCORED is a direct reduction (DR) process, which produces DRI (direct reduced iron) in a shaft furnace, either from natural gas (NG) or from reducing gas obtained by gasification of coal. This process combined with carbon capture and storage technology can minimize carbon dioxide emissions while minimizing energy consumption.	TRL=5-6	The combination of ULCORED process and CCS technology can reduce carbon dioxide emissions from blast furnaces by about 70%.	Its viability has been demonstrated at pilot and then demonstrator scales, which would take around 10-15 years or more	[107]

No.	Project	Type	Company	Implementation Region	Principle	Principle-details	Stage	Potential	Implementation time	Source
21	COREX	Carbon-reduction primary production technology	Voestalpine	Europe	SR without CO_2 benefits	COREX is a smelting reduction ironmaking process developed by voestalpine that uses lump ore or pellets as raw materials and non-coking coal as reductant and fuel.	TRL=7	Comparing to conventional blast furnace iron-making system, direct CO_2 emissions of COREX is higher.	Five operating plants	[108]
22	IGAR project	Carbon-circulation primary production technology	ArcelorMittal	Dunkirk, France	CCU pathway	capture waste CO_2 from the blast furnace and convert it into a synthetic gas (syngas) that can be reinjected into the blast furnace in place of fossil fuels to reduce iron ore	TRL=3-6	N.A.	ArcelorMittal is running project, supported by the French ADEME, to construct a plasma torch.	[109]
23	Carbalyst project	Carbon-circulation primary production technology	ArcelorMittal /LanzaTech	Europe	CCU pathway	The waste gases that result from iron and steelmaking are composed of the same molecular building blocks – carbon and hydrogen – used to produce the vast range of chemical products our society needs	TRL=5-6	CO_2 reduction of up to 87% compared with fossil transport fuels	Once completed in 2020, the facility will capture around 15% of the available waste gases at the plant and convert them into 80 million litres of ethanol per year	[109]
24	HYL-Energiron	Carbon-reduction primary production technology	Tenova HYL	Mexico	DR with external catalytic reformer	developed to allow reduction of iron ores in a shaft reactor without external gas reforming equipment. This process scheme has the ability to produce high carbon DRI, which allows producers to obtain maximum benefits of carbon in the steel making process while producing a product of higher stability	TRL=7-9	90% reduction of total CO_2	N.A.	[110]

No.	Project	Type	Company	Implementation Region	Principle	Principle-details	Stage	Potential	Implementation time	Source
25	Industrial emissions to sustainable ethanol	Carbon-circulation primary production technology	LanzaTech, Shougang Group and TangMing;	China	CCU pathway	First commercial plant began operation in 2018 in China, by produced 30 million litres of ethanol for sale in first year of operation	TRL=9	N.A. Capacity of 46,000 tons (16 million gallons) of ethanol per year, this facility will reduce carbon dioxide,	First commercial plant began operation in 2018	[111]
26	Carbon4PUR	Carbon-circulation primary production technology	Covestro, ArcelorMitta, Dechema	Europe	CCU pathway	transforming steel mill gas streams of the energy-intensive industry into higher value intermediates for market-oriented consumer products	TRL=6-8	20-60% emissions reduction	N.A. 2020	[112]
27	FReSMe	Carbon-circulation primary production technology	TataSteel, SSAB	Europe	CCU pathway	captures CO_2 from steel production for production of methanol fuel to be utilised in the ship transportation sector.	TRL=7-9	N.A.	early 2021	[113]
28	Steelanol	Carbon-circulation primary production technology	ArcelorMittal Primetals Technologies, Lanzatech, E4tech	Europe	CCU pathway	making industrial waste gases into liquid fuels, through biotech solutions for transformation of carbon monoxide to ethanol	TRL=7-9	Reduced direct emissions and 65% secondary reduction	N.A. early 2021	[114]
**Carbon-free EAF pathway**										
29	Rocky Mountain Steel Plant	Carbon-free secondary production technology	Evraz North America	USA	Carbon-free electricity pathway (solar-powered EAF)	A solar power generation facility will be jointly developed in Colorado, USA, to power the Rocky Mountain Steel Plant under Evraz, which will make the plant the first 100% solar-powered steel plant in the United States.	TRL 6-7	~55% CO_2 reduction	~ 2021 available	[115]

No.	Project	Type	Company	Implementation Region	Principle	Principle-details	Stage	Potential	Implementation time	Source
30	Nucor plant in Sedalia, Missouri	Carbon-free secondary production technology	Nucor Corporation	USA	Carbon-free electricity pathway (wind-powered EAF)	The Nucor plant will use energy produced by Evergy, including from a new wind farm, to power electric arc furnaces that will melt scrapped steel and turn it into new, recycled steels which set to be the first U.S. steel plant to run on wind energy.	TRL 6-7	100% CO_2 reduction if the mill's electricity supply were offset	N.A.	[115]
31	PEM (Primary Energy Melter)	Carbon-reduction secondary production technology	SMS Group, ArcelorMittal	Europe	Scrap treatment pathway	Enables melting of low-quality scrap with metallurgy/natural gas (pre-melting in shaft vessel, subsequent superheating process)	TRL=7	Potential CO_2 savings of 1 ton CO_2 per ton melted scrap	N.A. (Part of the Technology has been tested for decades. PEM installation expected 2019, integration in 2021)	[116]
**Biomass-based pathway**										
32	Replace coke with charcoal	Carbon-reduction primary production technology	Brazil	Brazil	Biomass-based pathway	Charcoal ironmaking and partial replacement of pulverized coal injection by pulverized charcoal or biofuel in coke blast furnace.	TRL=7	N.A. Perhaps higher than conventional process from life cycle perspective [117]	30% of 35.0 million t/year of pig iron produced in Brazil comes from charcoal "Mini Blast Furnace"	[118]
33	Torrefied biomass injection	Carbon-reduction primary production technology	ArcelorMittal	Belgium	Biomass-based pathway	producing bioethanol from a wood waste feedstock, fully integrated in a large-scale, industrially functional steel mill:	TRL=6-8	N.A.	the largescale demonstration is expected to be operational by end of 2020	[119]
**Blast Furnace-improvement pathway**										
33	Oxygen blast furnace (OBF) with CCS	CCS-assisted primary production technology	Ruukki Metals Ltd.	Finland	Blast Furnace-improvement pathway	The oxygen blast furnace (OBF) process is a unique process which uses pure oxygen instead of hot blast for ironmaking	TRL=2-5	Emissions can be reduced by 1.2 Mt/a without storing the separated CO_2	Model simulation phase	[120]

No.	Project	Type	Company	Implementation Region	Principle	Principle-details	Stage	Potential	Implementation time	Source
34	Top gas recycling blast furnace TGRBF/CCS	Carbon-circulation primary production technology	LKAB	Sweden	Blast Furnace-improvement pathway	lowering the use of fossil carbon (coke) via reuse of the reducing agents (CO and H2) after the removal of the CO_2 from the top gas, leading to lower energy requirements	TRL=7-9	carbon saving of 25%	Although the tests at the EBF are considered successful, the industrialization of the ULCOS-BF technology requires an additional scale- up step.	[121]
35	ROGESA pilot	Carbon-reduction primary production technology	Dillinger and Saarstahl	Germany	Blast Furnace-improvement pathway	Construct an innovative system to introduce a portion of the hydrogen-rich coke gas produced inside the integrated steel plant into the blast furnace.	TRL=5-7	N.A.<br>a significant reduction in carbon emissions.	implementation in two blast furnaces expected as early as 2020	[122]
**Low-carbon rolling technology**										
36	Hydrogen heating project	Carbon-reduction rolling technology	Linde Gas AB and Ovako	Sweden	Hydrogen in rolling preheating	Steel was heated using hydrogen instead of LPG (liquefied petroleum gas) before rolling at the mill in Hofors. The trial was successful and testing of the steel produced showed that heating with hydrogen does not affect the quality	TRL=7-9	N.A.<br>Save 20,000 tonnes of carbon dioxide each year	N.A.<br>Perform this trial in such a way that it can be reproduced at full scale in Hofors	[123]V
37	StaVari research project	Carbon-reduction rolling technology	EDAG Group	Germany	Reducing forming losses and light-weighting	Reducing yield losses in manufacturing (e.g. sheet metal in the automotive industry) would reduce material demand and in turn emissions from material production. Additive manufacturing, by its nature leads to minimal material losses compared to processes that cut an object from larger pieces of material.	TRL=4-6	N.A.	N.A.<br>There are plans to test the demonstrator in a real test as the project progresses.	[124]

S4.7 Summary of material efficiency strategies related to steel use

Material efficiency [125] refers to make more out of less material use, and the detailed measures have been widely examined for steel (around six types of strategies, i.e., Less Material Same Service, More Intensive Use, Lifespan Extension, Fabrication Scrap Diversion, Reuse of End-of-Life Scrap, and Yield Improvement) [48,125–127] that are considered as essential decarbonization strategy according to various assessments [128]. Here, we collected the detailed implementation strategies and some representative cases for the main end-use of steel in Table S4.5.

Table S4.5 Summary of material efficiency strategies on major end-use of steel

Strategies		Construction	Transportation	Machinery	Consumer goods
**1. Less Material Same Service**	Principle	• Using higher-strength rebar and optimize the sizing and placement of forcing systems to reduce steel usage.<br>• Use computer-aided tools to design the smallest quality components.<br>• Select components which are designed by DFM (design for manufacturability) techniques.<br>• Reduce the use of steel in consumer products through lightweight design.			
	Cases	Qube Design rationalizes the selection of reinforcing steel, etc.	Jaguar Land Rover's aim to reduce the weight of car doors by 30% within 5 years, Bombardier developed FLEXX Compact bogie.<br>Replacing steel with aluminum can reduce life cycle emissions due to weight reduction and subsequent fuel savings in the use phase, etc.	Assemble different components precisely instead of high-labor-cost welding, 3D printing, etc.	Aggressive light weighting of beverage can result in a 35% reduction in material requirement, etc.
**2. More Intensive Use**	Principle	• Make the building publicly accessible and improve the housing leasing system to reduce the demand for housing.<br>• Reduce the demand for private transportation through reasonable scheduling of public transportation and the availability of transportation.<br>• By renting production equipment instead of self-purchasing equipment, the idle rate of machines can be reduced. At the same time, enterprises can avoid the risk of technological backwardness and improve the efficiency and safety of construction.<br>• Using public goods to replace personal low-frequency goods can increase product utilization and reduce the mass production of the product.			

Strategies		Construction	Transportation	Machinery	Consumer goods
	Cases	Airbnb, Rental housing, Couch surfer, etc.	Increase the utilization rate of trains, Car-sharing, Ride-sharing, etc.	Construction machinery rental service, etc.	Shared power bank. Shared bicycle, etc.
**3. Lifespan Extension**	Principle	• Making components more durable, cascading products between users with different requirements or upgrading products to extend the useful life of their embedded materials<br>• Regularly check the operating conditions of vehicles and mechanical equipment to extend the service life.<br>• Use personal consumer products in a suitable environment to keep them in good condition.			
	Cases	Clear the roof after snowstorms, etc.	New metal is added to the rail in-situ in thin layers, and the rail can be restored with high strength, etc.	Regularly clean up mechanical equipment to extend the service life of mechanical equipment, etc.	Use personal consumer products in a suitable environment, avoid bumps, and avoid using them in rust-prone environments, etc.
**4. Fabrication Scrap Diversion**	Principle	Building materials will be surplus due to over-ordering. These extra materials can be made into other equipment.	The unused part of the vehicle manufacturing process can be collected and processed into other equipment.	Steel waste that does not become a product in the production process (such as trimming, trimming, etc.)	Consumer products are mass-produced, which require pre-ordered materials. The order quantity of raw materials is usually more than the actual quantity required.
	Cases	Extra pre-ordered steel bars can be made into a pedal net, etc.	Abbey Steel in Kettering purchased blanking skeletons and other trim (such as the window cut-outs in door panels) from car manufacturers, then cut regular shapes, supply them as blanks to firms making small parts, etc.	The roof trusses of the main stadium in 2012 London Olympics: are made from over-ordered oil and gas pipeline, etc.	The leftover material from making white goods can be used to make tables and chairs, etc.
	Principle	• Making components more durable, cascading products between users with different requirements or upgrading products to extend the useful life of their embedded materials<br>• Regularly check the operating conditions of vehicles and mechanical equipment to extend the service life.<br>• Use personal consumer products in a suitable environment to keep them in good condition.			
	Cases	Steel-framed buildings may be changed due to planning policies, and steel from	Shipbreakers will dismantle scrapped ships and obtain large amounts of steel and other metals	Recycling of mechanical processing equipment.	Reprocessing and use of consumer goods

Strategies		Construction	Transportation	Machinery	Consumer goods
		which can be used again. The British Construction Steelwork Association's new headquarters building was constructed from used steel			
**5. Reuse of End-of-Life Scrap**	Principle	Building materials will be surplus due to over-ordering. These extra materials can be made into other equipment.	The unused part of the vehicle manufacturing process can be collected and processed into other equipment.	Steel waste that does not become a product in the production process (such as trimming, trimming, etc.)	Consumer products are mass-produced, which require pre-ordered materials. The order quantity of raw materials is usually more than the actual quantity required.
	Cases	Extra pre-ordered steel bars can be made into a pedal net, etc.	Abbey Steel in Kettering purchased blanking skeletons and other trim (such as the window cut-outs in door panels) from car manufacturers, then cut regular shapes, supply them as blanks to firms making small parts, etc.	The roof trusses of the main stadium in 2012 London Olympics: are made from over-ordered oil and gas pipeline, etc.	The leftover material from making white goods can be used to make tables and chairs, etc.
**6. Yield Improvement**	Principle	• Reasonable planning can reduce product waste during construction.<br>• Using high-grade steel can improve performance while avoiding the use of large amounts of low-quality steel.<br>• Manage production materials well to avoid corrosion in their storage.			
	Cases	Qube Design rationalizes the layout of reinforcing steel.	High-strength steel for automobiles can subdivide component size and structural weight, save steel consumption, and is higher than ordinary-strength steel in terms of overall stability.	A novel technology for rolling create beams using one-third less metal than standard I-beams, Lean manufacturing, etc.	Optimize supply chain control links and maintain a reasonable inventory

Supplementary References

1. World Steel Association. *Energy use in the steel industry*. (2018).

2. Cullen, J. M., Allwood, J. M. & Bambach, M. D. Mapping the global flow of steel: from steelmaking to end-use good. *Environ. Sci. Technol.* **46**, 13048–55 (2012).

3. Pauliuk, S., Milford, R. L., Müller, D. B. & Allwood, J. M. The Steel Scrap Age. *Environ. Sci. Technol.* **47**, 3448–3454 (2013).

4. Wang, T., Müller, D. B. & Graedel, T. E. Forging the anthropogenic iron cycle. *Environ. Sci. Technol.* **41**, 5120–5129 (2007).

5. Eisele, T. C. & Kawatra, S. K. A review of binders in iron ore pelletization. *Miner. Process. Extr. Metall. Rev.* **24**, (2003).

6. Satyendra. Iron Ore Agglomeration Processes and their Historical Development. (2015).

7. Satyendra. Evolution of Blast Furnace Iron Making. (2016).

8. Smil, V. *Transforming the twentieth century: technical innovations and their consequences*. **2**, (Oxford University Press on Demand, 2006).

9. Pardo, N., AntonioMoya, J. & Vatopoulos, K. *Prospective Scenarios on Energy Efficiency and CO2 Emissions in the EU Iron & Steel Industry*. (2012).

10. Beer, J. De, Worrell, E. & Blok, K. Future Technologies for Energy-Efficient Iron and Steel Making. *Annu. Rev. Energy Environ.* **23**, 123–205 (1998).

11. Smil, V. *Energy Transitions: Global and National Perspectives*. (Praeger, 2016).

12. Naito, M., Takeda, K. & Matsui, Y. Ironmaking Technology for the Last 100 Years: From Adopted Technologies to a Position of Leadership in Advanced and Next-Generation Technology. *Tetsu-to-Hagane* **100**, 2–30 (2014).

13. U.S. Geological Survey. Historical statistics for mineral and material commodities in the United States: Iron ore (through 2015). (2017). Available at: https://minerals.usgs.gov/minerals/pubs/historical-statistics/#format. (Accessed: 26th September 2017)

14. Gutowski, T. G. Energy and Carbon Use in Manufacturing. in *40th CIRP International Manufacturing Systems Seminar at Liverpool University 30 May to 1 June in 2007* (2007).

15. Orth, A., Anastasijevic, N. & Eichberger, H. Low CO2emission technologies for iron and steelmaking as well as titania slag production. *Miner. Eng.* **20**, 854–861 (2007).

16. Emi, T. Steelmaking Technology for the Last 100 Years: Toward Highly Efficient Mass Production Systems for High Quality Steels. *ISIJ Int.* **55**, 36–66 (2015).

17. Naito, M., Takeda, K. & Matsui, Y. Ironmaking Technology for the Last 100 Years: Deployment to Advanced Technologies from Introduction of Technological Know-how, and Evolution to Next-generation Process. *ISIJ Int.* **55**, 7–35 (2015).

18. Hasanbeigi, A., Arens, M. & Price, L. Alternative emerging ironmaking technologies for energy-efficiency and carbon dioxide emissions reduction: A technical review. *Renew. Sustain. Energy Rev.* **33**, 645–658 (2014).

19. Midrex. *World Direct Reduction Statistics 2016*. (2017).

20. Föll, H. Making Steel after 1870. *Iron, Steel and Swords* (2012).

21. Piaskowski, J. & Piaskowska, H. Technometry as a methodology of the history of technology. *Hist. Technol. an Int. J.* **2**, 295–308 (1986).

22. Fruehan, R. J. *The Making, Shaping, and Treating of Steel.* (AISE, 1985).

23. Grübler, A. *Technology and Global Change.* (Cambridge University Press, 2003). doi:10.1017/CBO9781316036471

24. Rosenberg, N. Energy efficient technologies: past and future perspectives. in *Innovation, resources and economic growth* (eds. Curzio, A. Q., Fortis, M. & Zoboli, R.) 63–82 (Springer, 1994).

25. Fruehan, R. Overview of steelmaking processes and their development. in *The Making, Shaping and Treating of Steel* (AISE, 1998).

26. Maddala, G. S. & Knight, P. T. International Diffusion of Technical Change-A Case Study of the Oxygen Steel Making Process. *Econ. J.* **77**, 531–558 (1967).

27. Birat, J. P. Steel industry: culture and futures. in *8th European Continuous Casting Conference* 23–25 (2014).

28. World Steel Association. *World Steel Yearbook*. (2017).

29. Pauliuk, S., Wang, T. & Müller, D. B. Steel all over the world: Estimating in-use stocks of iron for 200 countries. *Resour. Conserv. Recycl.* **71**, 22–30 (2013).

30. Mohr, S., Giurco, D., Yellishetty, M., Ward, J. & Mudd, G. Projection of Iron Ore Production. *Nat. Resour. Res.* **24**, 317–327 (2015).

31. Cheeley, R. Gasification and the Midrex Direct Reduction Process. in *1999 Gasification Technologies Conference* (1999).

32. Tenova. Energiron: DRI technology by Tenova and Danieli. 52 (2014). Available at: https://www.energiron.com/. (Accessed: 15th January 2021)

33. Dey, N. R., Prasad, A. K. & Singh, S. K. Energy survey of the coal based sponge iron industry. *Case Stud. Therm. Eng.* **6**, 1–15 (2015).

34. USS. *The Making, Shaping and Treating of Steel (1st-11st edition).* (United States Steel Corporation, 1919).

35. Zhou, D. G., Wang, L. G. & Zhou, S. Z. Measurement of heat loss and calculation of thermal efficiency of a huge open hearth furnace in Chongsteel. *Iron Steel(in Chinese)* 27–30 (1966).

36. Cullen, J. M. & Allwood, J. M. Mapping the global flow of aluminium: from liquid aluminium to End-Use goods. *Environ. Sci. Technol.* **47**, 3057–3064 (2013).

37. Camp, J. M. & Francis, C. B. *The making, shaping and treating of steel(6th Edition).* (Carnegie Steel

Company, 1951).

38. Michaelis, P. & Jackson, T. Material and energy flow through the UK iron and steel sector. Part 1: 1954-1994. *Resour. Conserv. Recycl.* **29**, 131–156 (2000).

39. OTA. *Industrial Energy Efficiency*. (U.S. Congress, Office of Technology Assessment(OTA), 1993).

40. Stubbles, J. *Energy use in the US steel industry: an historical perspective and future opportunities*. (EERE Publication and Product Library, 2000).

41. Mudd, G. M. The limits to growth and 'finite' mineral resources: re-visiting the assumptions and drinking from that half-capacity glass. *Int. J. Sustain. Dev.* **16**, 204 (2013).

42. U.S. Geological Survey. *Global iron ore production data*. (2017).

43. United States Census Bureau. Statistical Abstract of the United States. (1969).

44. Wang, P., Li, W. & Kara, S. Dynamic life cycle quantification of metallic elements and their circularity, efficiency, and leakages. *J. Clean. Prod.* **174**, 1492–1502 (2018).

45. Daigo, I., Iwata, K., Ohkata, I. & Goto, Y. Macroscopic Evidence for the Hibernating Behavior of Materials Stock. *Environ. Sci. Technol.* **49**, 8691–8696 (2015).

46. Graedel, T. E. *et al.* What do we know about metal recycling rates? *J. Ind. Ecol.* **15**, 355–366 (2011).

47. Classen, M. *et al.* Life Cycle Inventories of Metals Data v2.1 (2009). *ecoinvent v2.1 Rep. No. 10* 1–926 (2009). doi:10.1065/lca2004.11.181.5

48. Milford, R. L., Pauliuk, S., Allwood, J. M. & Müller, D. B. The Roles of Energy and Material Efficiency in Meeting Steel Industry CO2 Targets. *Environ. Sci. Technol.* **47**, 3455–3462 (2013).

49. Worrell, E., Price, L., Neelis, M., Galitsky, C. & Zhou, N. *World Best Practice Energy Intensity Values for Selected Industrial Sectors*. (2007).

50. Arens, M., Worrell, E. & Schleich, J. Energy intensity development of the German iron and steel industry between 1991 and 2007. *Energy* **45**, 786–797 (2012).

51. Meunier, M. Y., de Bruyn Kops, O., Meunier, M. & Y.de Bruyn Kops, O. *Energy efficiency in the steel industry with emphasis on developing countries*. (1984).

52. Jones, A. J. The industrial ecology of the iron casting industry. (Massachusetts Institute of Technology, 2007).

53. World Steel Association. Sustainability Indicators 2003-2016. *Sustainability indicators* (2017). Available at: https://www.worldsteel.org/steel-by-topic/sustainability/sustainability-indicators0.html. (Accessed: 30th October 2017)

54. International Energy Agency. Tracking Clean Energy Progress: Iron and steel. (2018). Available at: https://www.iea.org/reports/iron-and-steel. (Accessed: 23rd July 2018)

55. International Energy Agency. Energy Sankey World Balance (1973-2015) with steel production data. *2018*

56. He, K., Wang, L., Zhu, H. & Ding, Y. Energy-saving potential of China's steel industry according to its development plan. *Energies* (2018). doi:10.3390/en11040948

57. Margolis, N. & Sousa, L. Energy and environmental profile of the U.S. iron and steel industry. in *Proceedings ACEEE Summer Study on Energy Efficiency in Industry* (1997).

58. Cavaliere, P. *Ironmaking and steelmaking processes: Greenhouse emissions, control, and reduction. Ironmaking and Steelmaking Processes: Greenhouse Emissions, Control, and Reduction* (2016). doi:10.1007/978-3-319-39529-6

59. International Steel Statistics Bureau. *Key Statistics 2015*. (2015).

60. Liu, S. & Liu, Y. An analysis on the Energy Consumption of Masteel's 300 m2 sinter and energy saving approaches. *Anhui Yejin* **2**, 42–45 (2011).

61. Zeng, C. ., Liu, M. . & Li, J. . Production Practice of Reducing Pellet Process Consumption for Hansteel Phase Two Pelletizing Project. *Sinter. Pelletizing* **37**, 53–57 (2012).

62. Stepanov, V. S. *Analysis of energy efficiency of industrial processes*. (Springer Science & Business Media, 2012).

63. Hocking, M. B. B. *Handbook of chemical technology and pollution control*. (Elsevier, 2016).

64. Thollander, P., Karlsson, M., Söderström, M. & Creutz, D. Reducing industrial energy costs through energy-efficiency measures in a liberalized European electricity market: case study of a Swedish iron foundry. *Appl. Energy* **81**, 115–126 (2005).

65. Stubbles, J. R. *The US Steel Industry: Energy Consumption and CO2 Emissions*. (2001).

66. Myhre, G. *et al.* Anthropogenic and Natural Radiative Forcing. in *Climate Change 2013: The Physical Science Basis. Contribution of Working Group I to the Fifth Assessment Report of the Intergovernmental Panel on Climate Change* (eds. Stocker, T. F. et al.) 659–740 (Cambridge University Press, 2013).

67. World Bank. Electricity production from hydroelectric sources (% of total). (2014). Available at: https://data.worldbank.org/indicator/EG.ELC.HYRO.ZS. (Accessed: 28th November 2017)

68. International Energy Agency. World. Electricity and heat. Available at: https://www.iea.org/statistics/statisticssearch/report/?country=WORLD&product=electricityandheat&year=2015. (Accessed: 28th November 2017)

69. World Bank. World Development Indicators. *Electricity production from different sources* (2014).

70. Fouquet, R. The slow search for solutions: Lessons from historical energy transitions by sector and service. *Energy Policy* **38**, 6586–6596 (2010).

71. Bhattacharjee, S. & Vasudevan, N. Energy efficiency considerations in the Indian foundry industry. in *Energy Conversion Engineering Conference, 1996. IECEC 96., Proceedings of the 31st Intersociety* **4**, 2263–2268 (IEEE, 1996).

72. Oda, J., Akimoto, K. & Tomoda, T. Long-term global availability of steel scrap. *Resour. Conserv. Recycl.* **81**, 81–91 (2013).

73. Hatayama, H., Daigo, I., Matsuno, Y. & Adachi, Y. Outlook of the world steel cycle based on the stock and flow dynamics. *Environ. Sci. Technol.* **44**, 6457–6463 (2010).

74. Müller, D. B., Wang, T. & Duval, B. Patterns of iron use in societal evolution. *Environ. Sci. Technol.* **45**, 182–188 (2011).

75. Neelis, M. & Patel, M. *Long-term production, energy consumption and CO2 emission scenarios for the worldwide iron and steel industry.* (Copernicus Institute for Sustainable Development and Innovation, 2006).

76. World Steel Association. *The three Rs of sustainable steel. World Steel Association* (2010).

77. Kozawa, S. & Tsukihashi, F. Analysis of global demand for iron source by estimation of in-use steel stock. *ISIJ Int.* **51**, 320–329 (2011).

78. World Steel Association. *SUSTAINABILITY INDICATORS 2003 - 2016*. (World Steel Association, 2018).

79. Worrell, E., Price, L. & Martin, N. *Energy Efficiency and Carbon Dioxide Emissions Reduction Opportunities in the U.S. Iron and Steel Sector.* (Ernest Orlando Lawrence Berkeley National Laboratory, 1999). doi:10.1016/S0360-5442(01)00017-2

80. IEA GHG. *Greenhous Gas Emissions from Major Industrial Sources – III IRON AND STEEL PRODUCTION Report Number PH3/30.* (IEA Greenhouse Gas R&D programme, 2000). doi:PH3/30

81. Price, L., Michaelis, L., Worrell, E. & Krhushch, M. Sectoral trends and driving forces of global energy use and greenhous gas emissions. *Mitig. Adapt. Strateg. Glob. Chang.* **3**, 263–319 (1998).

82. Hasanbeigi, A. & Springer, C. *How Clean Is the U.S. Steel Industry? An International Benchmarking of Energy and CO2 Intensities.* (2019).

83. Chen, Q., Gu, Y., Tang, Z., Wei, W. & Sun, Y. Assessment of low-carbon iron and steel production with CO2 recycling and utilization technologies: A case study in China. *Appl. Energy* **220**, 192–207 (2018).

84. OECD. OECD Steelmaking Capacity Database, 2000-2019. (2020).

85. International Energy Agency. *Iron and Steel Technology: Towards more sustainable steelmaking.* (2020).

86. Rogelj, J., Forster, P. M., Kriegler, E., Smith, C. J. & Séférian, R. Estimating and tracking the remaining carbon budget for stringent climate targets. *Nature* **571**, 335–342 (2019).

87. International Energy Agency. ETP Clean Energy Technology Guide. (2020). Available at: https://www.iea.org/articles/etp-clean-energy-technology-guide. (Accessed: 15th February 2021)

88. Bataille, C. G. F. Physical and policy pathways to net-zero emissions industry. *Wiley Interdiscip. Rev. Clim. Chang.* **11**, 1–20 (2020).

89. Pei, M., Petäjäniemi, M., Regnell, A. & Wijk, O. Toward a fossil free future with hybrit: Development of iron and steelmaking technology in Sweden and Finland. *Metals (Basel).* **10**, 1–11 (2020).

90. Bataille, C. *Low and zero emissions in the steel and cement industries Barriers, technologies and policies*. *Organisation*

for Economic Co-operation and Development OECD (2019).

91. Salzgitter AG. *Special edition on Hydrogen and SALCOS*. (2019).

92. Thyssenkrupp Steel. Thyssenkrupp Steel's climate strategy for sustainable steel production. (2020).

93. HBIS Group. HBIS & Tenova Upgrading Tradtional Steel Making Technology With Hydrogen. (2019).

94. Asian Steel. China Baowu signs cooperation agreement with CNNC and Tsinghua University. (2019).

95. Zhong, F. How hydrogen is gaining momentum in the Chinese steel industry. *Worldsteel news* (2020).

96. Komera. KOMERA Website. (2020).

97. Ripke, J. & Kopfle, J. MIDREX H2: Ultimate Low CO2 Ironmaking and its place in the new Hydrogen Economy. (2017).

98. EU. *H2FUTURE: A European Flagship Project for Generation and Use of Green Hydrogen*. (2019).

99. K1-MET GmbH. Project Susteel:Sustainable steel production utilising hydrogen. (2020).

100. Inagaki, Y., Kasahara, S. & Ogawa, M. Merit assessment of nuclear hydrogen steelmaking with very high temperature reactor. *ISIJ Int.* **52**, 1420–1426 (2012).

101. Diczfalusy, Bo Wisniewski, J. Energy Consumption and CO2 Emissions in Ironmaking and Development of a Novel Flash Technology. *Metals (Basel).* **10**, 1–22 (2020).

102. Lavelaine, H. *ΣIDERWIN project: electrification of primary steel production for direct CO2 emission avoidance*. (2019).

103. Boston Metal. Metal Oxide Electrolysis. (2020).

104. Zahra, M. A. *Perspective from Emirates Steel Project/UAE*. (2015).

105. Tata Steel. *HISARNA: Building a sustainable steel industry*. (2020). doi:www.tatasteeleurope.com/en/innovation/hisarna

106. POSCO. *The Finex process - economical and environmentally at lower costs and emissions*. (2020).

107. Steel360. ULCORED Process. (2019).

108. Primetals. *COREX: Efficient and Environmentally Friendly Smelting Reduction*. (2020).

109. ArcelorMittal. *ArcelorMittal strategy towards low-emissions steelmaking*. (2020).

110. Duarte, P. E., Tavano, A. & Zendejas, E. Achieving Carbon Free Emissions via the ENERGIRON DR Process. in *AISTech 2010, proceedings of the iron and steel technology conference,* 1–12 (2010).

111. ACLIMAADMIN. World's First Commercial Waste Gas to Ethanol Plant Now in Operation. (2018).

112. Carbon4PUR. *Carbon4PUR - Turning industrial waste gases (mixed CO/CO2 streams) into intermediates for polyurethane plastics for rigid foams/building insulation and coatings*. (2018).

113. Bonalumi, D. *et al.* From Residual Steel gases to Methanol: The FReSMe project. in *14th International*

Conference on Greenhouse Gas Control Technologies (2018).

114. Steelanol. *Steelanol: Fuel a Sustianable Future.* (2020).

115. Douglas, J. First US steel plants powered by wind, solar energy are coming for industry with big carbon footprint. (2019).

116. Thiede, C. *et al.* Energy efficient steel production with the Primary Energy Melter(PEM). in *7th INTERNATIONAL IRON & STEEL SYMPOSIUM* 88–91 (2017).

117. Sonter, L. J., Barrett, D. J., Moran, C. J. & Soares-Filho, B. S. Carbon emissions due to deforestation for the production of charcoal used in Brazil's steel industry. *Nat. Clim. Chang.* **5**, 359–363 (2015).

118. Scarpinella, C. A., Takano, C., Solon, Y., Mourao, M. B. & Guilherme, F. B. Charcoal ironmaking: a contribution for CO2 mitigation. *Met. Mater. Process. a Clean Environ.* **2**, 109–121 (2011).

119. Arcelormittal. TORrefying wood with Ethanol as a Renewable Output: large-scale demonstration. (2017).

120. Arasto, A., Tsupari, E., Kärki, J., Lilja, J. & Sihvonen, M. International Journal of Greenhouse Gas Control Oxygen blast furnace with CO 2 capture and storage at an integrated steel mill — Part I : Technical concept analysis. *Int. J. Greenh. Gas Control* **30**, 140–147 (2014).

121. Van Der Stel, J. *et al.* Top gas recycling blast furnace developments for 'green' and sustainable ironmaking. *Ironmak. Steelmak.* **40**, 483–489 (2013).

122. Dillinger. Sustainable steel production at the Saar. (2019).

123. Linde. Linde & Ovako Heat Steel With Hydrogen In World First. (2020).

124. Hema. Results of the StaVari research project at IAA 2019. (2019).

125. Allwood, J. M., Ashby, M. F., Gutowski, T. G. & Worrell, E. Material efficiency: A white paper. *Resour. Conserv. Recycl.* **55**, 362–381 (2011).

126. Hertwich, E. G. *et al.* Material efficiency strategies to reducing greenhouse gas emissions associated with buildings, vehicles, and electronics — a review. *Environ. Res. Lett.* **14**, 043004 (2019).

127. Pauliuk, S. *et al.* Linking service provision to material cycles: A new framework for studying the resource efficiency–climate change (RECC) nexus. *J. Ind. Ecol.* jiec.13023 (2020). doi:10.1111/jiec.13023

128. Allwood, J. M., Cullen, J. M. & Milford, R. L. Options for achieving a 50% cut in industrial carbon emissions by 2050. *Environ. Sci. Technol.* **44**, 1888–1894 (2010).
