# Supplementary material for: Efficiency stagnation in global steel production urges joint supply- and demand-side mitigation efforts
Source: Nat Commun. 2021 Apr 6;12:2066. doi: 10.1038/s41467-021-22245-6 (PMC8024266; doi:10.1038/s41467-021-22245-6)
Supplement: Supplementary file 3 — Description of Additional Supplementary Files [file 41467_2021_22245_MOESM3_ESM.pdf]

Description of Additional Supplementary Files

File Name: Supplementary Data 1

Description: Overview of lifecycle inventory (LCI) used for estimating greenhouse gas emissions associated with the steel production technologies covered in this study and used for quantifying greenhouse gas emissions from global steel production from 1900 to 2015.
