# Supplementary material for: Efficiency stagnation in global steel production urges joint supply- and demand-side mitigation efforts
Source: Nat Commun. 2021 Apr 6;12:2066. doi: 10.1038/s41467-021-22245-6 (PMC8024266; doi:10.1038/s41467-021-22245-6)
Supplement: Supplementary file 5 — Reporting Summary [file 41467_2021_22245_MOESM5_ESM.pdf]

nature research

Corresponding author(s): Morten Ryberg
Sami Kara
Wei-Qiang Chen

Last updated by author(s): Feb 12, 2021

Reporting Summary

Nature Research wishes to improve the reproducibility of the work that we publish. This form provides structure for consistency and transparency in reporting. For further information on Nature Research policies, see our Editorial Policies and the Editorial Policy Checklist.

Statistics

For all statistical analyses, confirm that the following items are present in the figure legend, table legend, main text, or Methods section.

n/a	Confirmed	
x		The exact sample size (*n*) for each experimental group/condition, given as a discrete number and unit of measurement
x		A statement on whether measurements were taken from distinct samples or whether the same sample was measured repeatedly
x		The statistical test(s) used AND whether they are one- or two-sided<br>*Only common tests should be described solely by name; describe more complex techniques in the Methods section.*
x		A description of all covariates tested
x		A description of any assumptions or corrections, such as tests of normality and adjustment for multiple comparisons
	x	A full description of the statistical parameters including central tendency (e.g. means) or other basic estimates (e.g. regression coefficient) AND variation (e.g. standard deviation) or associated estimates of uncertainty (e.g. confidence intervals)
x		For null hypothesis testing, the test statistic (e.g. *F*, *t*, *r*) with confidence intervals, effect sizes, degrees of freedom and *P* value noted<br>*Give P values as exact values whenever suitable.*
x		For Bayesian analysis, information on the choice of priors and Markov chain Monte Carlo settings
x		For hierarchical and complex designs, identification of the appropriate level for tests and full reporting of outcomes
x		Estimates of effect sizes (e.g. Cohen's *d*, Pearson's *r*), indicating how they were calculated

Our web collection on statistics for biologists contains articles on many of the points above.

Software and code

Policy information about availability of computer code

Data collection	The present study does not involve the collection of new raw data.
Data analysis	SimaPro 9.0.0.35;<br>Matlab R2015b;<br>Microsoft Excel 2016;<br>Adobe Illustrator 25.2

For manuscripts utilizing custom algorithms or software that are central to the research but not yet described in published literature, software must be made available to editors and reviewers. We strongly encourage code deposition in a community repository (e.g. GitHub). See the Nature Research guidelines for submitting code & software for further information.

Data

Policy information about availability of data

All manuscripts must include a data availability statement. This statement should provide the following information, where applicable:

- Accession codes, unique identifiers, or web links for publicly available datasets
- A list of figures that have associated raw data
- A description of any restrictions on data availability

The source data supporting the findings of this study are available within the paper, and its supplementary information files.
Data used for the Material Flow Analysis is described in Supplementary Information 1 Section S2.1 and Table S2.1. Data for constructing life-cycle inventory of greenhouse gas emissions from each technology was based on ecoinvent v.3.1 as modelled in SimaPro 9.0.0.35 coupled with technology specific literature as described in given in supplementary information 1. The main results for our material flow analysis and greenhouse gas emission estimates are given in Section S3-S4 of supplementary information 1. Detailed information about the data used for the life-cycle inventory for each technology is given in Supplementary Data 1 in MS

Excel format. Source data for figures are provided as supplementary source files. Correspondence and requests for other information and/or materials should be addressed to Morten Ryberg.

Field-specific reporting

Please select the one below that is the best fit for your research. If you are not sure, read the appropriate sections before making your selection.

- [ ] Life sciences
- [ ] Behavioural & social sciences
- [x] Ecological, evolutionary & environmental sciences

For a reference copy of the document with all sections, see nature.com/documents/nr-reporting-summary-flat.pdf

Ecological, evolutionary & environmental sciences study design

All studies must disclose on these points even when the disclosure is negative.

Study description	We integrated dynamic material flow analysis (MFA) with life-cycle assessment (LCA) to estimate annual production, efficiency, and emissions of global steel production based on 19 dominant technologies during 1900-2015. Material flow analysis is conducted to quantify the material inflows, outflows, and stocks along global steel life cycle under the principle of mass balance, which can generate the production flows from studied19 dominant technologies. For each steel production technology, we quantified both direct and indirect GHG emissions per mass unit output on an annual basis. The GHG emissions inventory we compiled draws primarily on unit process data from the Ecoinvent v.3.0. We then applied the Logarithmic Mean Divisia Index (LMDI) decomposition method to quantify the influence of changes in production volume and GHG emission intensity for the two main production routes on the absolute emission change.<br>The present study does not involve the collection of new raw data and data sampling.
Research sample	The present study does not involve the collection of new raw data and data sampling..<br>The data sources for the material flow analysis and life-cycle assessment, and the details can be found in supplementary information 1 and 2.
Sampling strategy	The present study does not involve the collection of new raw data and data sampling. Our research is not based on experiments, so this report item does not apply to our research.
Data collection	The present study does not involve the collection of new raw data. Collection of data for the study was based on a wide range of existing data sources as described in Methods section and Supplementary Information 1
Timing and spatial scale	The temporal scale of our analysis is 1900-2015. The spatial scale is global. The temporal scale was based on data availability.
Data exclusions	The present study does not involve the collection of new raw data and data sampling. Thus, no data exclusion was used
Reproducibility	Our research is not based on experiments, so this report item does not apply to our research.
Randomization	The present study does not involve allocation of samples and randomization control.
Blinding	The present study does not involve data acquisition and blinding.

Did the study involve field work? [ ] Yes [x] No

Reporting for specific materials, systems and methods

We require information from authors about some types of materials, experimental systems and methods used in many studies. Here, indicate whether each material, system or method listed is relevant to your study. If you are not sure if a list item applies to your research, read the appropriate section before selecting a response.

Materials & experimental systems

n/a	Involved in the study	
[x]	[ ]	Antibodies
[x]	[ ]	Eukaryotic cell lines
[x]	[ ]	Palaeontology and archaeology
[x]	[ ]	Animals and other organisms
[x]	[ ]	Human research participants
[x]	[ ]	Clinical data
[x]	[ ]	Dual use research of concern

Methods

n/a	Involved in the study	
[x]	[ ]	ChIP-seq
[x]	[ ]	Flow cytometry
[x]	[ ]	MRI-based neuroimaging
